# Metal-dependent Structure, Solution Properties, and Redox-Mediated Anticancer Activity of Rhodium(III) and Iridium(III) Half-Sandwich Phenanthroline–Sulfonamide Complexes

**DOI:** 10.1021/acs.inorgchem.6c04078

**Published:** 2026-10-02

**Authors:** Natalia Żukowska, Mateusz Kowalik, Natalia Maciejewska, Jan Alfuth, Dawid Dębowski, Katarzyna Kazimierczuk, Mariusz Makowski

**Affiliations:** † Faculty of Chemistry, 49646 University of Gdańsk, Wita Stwosza 63, 80-308 Gdańsk, Poland; ‡ Faculty of Chemistry, 431446 Gdańsk University of Technology, Gabriela Narutowicza 11/12, 80-233 Gdańsk, Poland

## Abstract

A phenanthroline-bearing sulfonamide ligand (**L**) and its half-sandwich rhodium­(III) and iridium­(III) complexes, [(η^5^-Cp*)­RhCl­(L)]­PF_6_ (**1**) and [(η^5^-Cp*)­IrCl­(L)]­PF_6_ (**2**), were synthesized to determine how metal coordination and metal identity influence structure, solution properties, and anticancer responses. Single-crystal X-ray diffraction established closely related piano-stool geometries with bidentate N,N-coordination of **L**, while spectroscopic analyses confirmed the complexes in solution. All compounds were spectroscopically stable for 72 h under the tested conditions. Coordination shifted the lipophilicity of **L** (log *P* = 0.78) to hydrophilic values for **1** and **2** (−0.65 and −0.91, respectively). The complexes weakly inhibited carbonic anhydrase II and displayed low-affinity binding to human serum albumin; the CT-DNA UV–vis experiment was inconclusive because of substantial spectral overlap and was not used to assign a DNA-binding mode. **L** showed submicromolar-to-low-micromolar cytotoxicity across four cancer cell lines and pronounced time-dependent mitochondrial depolarization in NALM-6 cells. Complex **2** retained micromolar potency but showed weak overall cancer/nonmalignant discrimination, whereas **1** showed a marked preference for NALM-6 cells (IC_50_ = 4.41 μM; HEK-293 IC_50_ > 100 μM). In NALM-6 cells, **1** produced a pronounced ROS increase at 6 h that preceded prominent γH2AX signaling, caspase-3/7 activation, and apoptosis at 24–48 h, while mitochondrial depolarization remained comparatively limited. This temporal ordering is consistent with a redox-associated cellular response but does not by itself establish ROS causality or exclude mitochondrial involvement. These matched analogs demonstrate that Rh­(III) versus Ir­(III) coordination can reprogram physicochemical and cellular response profiles within the same ligand scaffold and identify **1** as a candidate for further mechanism-guided optimization.

## Graphical abstract

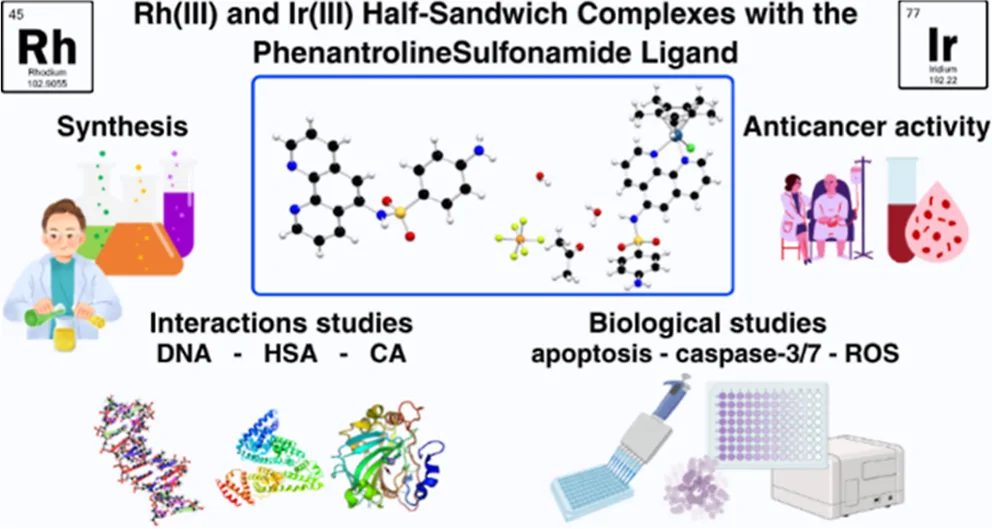

## Introduction

Cancer remains a major global health challenge, with the annual number of new cases projected to increase from 19.3 million in 2020 to over 30 million by 2040.^1^ Despite significant advances in cancer therapy, drug resistance and off-target toxicity continue to limit the effectiveness of current chemotherapeutic strategies, highlighting the need for novel, selective, and multifunctional agents capable of acting through complementary or synergistic mechanisms. Among the molecular scaffolds attracting renewed interest in anticancer drug discovery are sulfonamides, particularly arylsulfonamides.^2^ Historically, sulfonamides were introduced as potent antimicrobial agents, but their clinical utility has been compromised by increasing bacterial resistance and safety concerns.^3^ However, the growing interest in drug repurposing has revealed the broader pharmacological potential of these compounds, including anti-inflammatory, antioxidant, antiviral, antidiabetic, and anticancer activities.^3,4^ The sulfonamide group is considered a valuable pharmacophore in medicinal chemistry due to its ability to participate in hydrogen-bonding interactions, mimic carboxylic acid functionalities, and improve metabolic stability and membrane permeability. Moreover, structural modification of the attached groups enables tuning of pharmacokinetic properties, potency, and selectivity.^5,6^ The sulfonamides present diverse anticancer mechanisms of action, including inhibition of tyrosine kinases, aromatase, carbonic anhydrases, matrix metalloproteinases, and histone deacetylases, affecting key pathways involved in cancer progression.^2,5,7^ Additionally, the sulfonamide moiety can function as a pharmacologically relevant scaffold and as a linking unit, connecting diverse chemical structures within a single molecule. This well-established medicinal chemistry strategy enables the design of complex sulfonamide-based molecules with novel biological activities and optimized pharmacokinetic properties.^6,8–10^ Such hybrid compounds can be engineered to incorporate chemical motifs that modulate membrane permeability, enhance interactions with biological targets such as enzymes or DNA, and ultimately strengthen therapeutic effects while reducing toxicity. One such modification involves complexation with metal ions.^11^ Metal-centered compounds, with their inherent features, complex structures, and geometries, offer access to chemical reactivity and mechanisms of action that are generally unavailable to purely organic compounds such as sulfonamides.^12,13^ The well-known metal-based drugs, cisplatin and its derivatives, carboplatin and oxaliplatin, remain widely used anticancer agents that act primarily through DNA cross-linking. However, their clinical application is limited by poor selectivity, severe side effects, and the development of drug resistance.^14–16^ A typical treatment strategy for platinum-resistant cancers relies on nonplatinum-based chemotherapy, administered alone or in combination with other agents, such as targeted delivery systems.^17^ These limitations have triggered the search for alternative metal-based anticancer agents with distinct mechanisms of action and improved therapeutic profiles.^18,19^ Among these, organometallic complexes containing transition metals such as Rh­(III) and Ir­(III) have attracted considerable attention as potential alternatives to platinum-based drugs due to their diverse reactivity, tunable coordination chemistry, and potential to overcome limitations associated with conventional platinum-based therapies.^20–23^ Iridium­(III) complexes, particularly half-sandwich structures, exhibit promising anticancer activity through mechanisms involving ROS generation, mitochondrial dysfunction, and apoptosis, whereas rhodium­(III) complexes provide unique structural and catalytic properties that may be exploited for the development of selective anticancer agents.^24–27^ Overall, research on compounds containing Ir­(III) and Rh­(III) is rapidly expanding, with particular focus on designing complexes with greater potency and selectivity for cancer cells than traditional platinum-based agents. A central strategy in this field involves fine-tuning metal-complex properties, including the metal center, oxidation state, and coordinating ligand environment, to modulate stability, reactivity, and biological mode of action.^28^ Among the various structural motifs explored, organometallic half-sandwich complexes, with the general formula [(η^5^-Cp*)­MCl­(XY)] (where *M* = Rh­(III) or Ir­(III); Cp* = pentamethylcyclopentadienyl; XY = chelating ligand), have emerged as especially promising scaffolds. The choice of bidentate (XY) and Cp* ligands can significantly influence both reactivity and biological activity.^29^ An example of such a ligand is 1,10-phenanthroline-5-amine, which has been shown to possess a wide range of activities, including anticancer and antibacterial properties, and which can then be used as a basis for a new derivative.^30–35^ Metal-sulfonamide complexes are promising anticancer scaffolds, exhibiting enhanced cytotoxicity against cancer cells and reduced toxicity toward nonmalignant cells compared with free ligands or drugs such as cisplatin.^28^ They can inhibit cancer-associated enzymes (such as carbonic anhydrase IX and XII), induce cell-cycle arrest, DNA damage, ROS accumulation, disrupt tumor-related proteins, and trigger mitochondrial membrane depolarization.^36–41^

In our recent studies^41,42^ we demonstrated that sulfonamide-based ligands can be incorporated into Rh­(III) and Ir­(III) half-sandwich complexes to generate stable chelating systems with tunable physicochemical and biological properties. In particular, a matched Rh­(III)/Ir­(III) pair bearing a bipyridylsulfonamide ligand showed that changing only the metal center can markedly alter biomolecular interactions and antiproliferative behavior.^42^ Metal-dependent divergence has also been reported for other isostructural Rh­(III)/Ir­(III) half-sandwich complexes bearing identical N,N-chelating ligands.^29^ These observations establish metal identity as a potentially active determinant of biological behavior rather than a passive structural substitution.

Despite these precedents, a direct structure– property– activity comparison of matched Rh­(III) and Ir­(III) half-sandwich complexes containing the same phenanthroline–sulfonamide chelate remains insufficiently defined. This distinction matters because replacing a bipyridyl donor with a more rigid, extended phenanthroline framework can alter solution properties, biomolecular interactions, and cellular behavior even when the overall piano-stool coordination motif is retained. Moreover, although ROS generation, mitochondrial dysfunction, DNA-damage signaling, and apoptosis have individually been described for organometallic anticancer agents, their temporal relationship has been less systematically examined for Rh­(III) sulfonamide systems.^24–29,36–42^ A matched **L**/**1**/**2** series therefore provides a controlled platform to separate the effects of ligand coordination from those of Rh­(III)-to-Ir­(III) metal exchange and to compare the timing of cellular stress responses without assuming a priori that one pathway is causative.

The aim of this study was to synthesize and comprehensively characterize 4-amino-N-(1,10-phenanthrolin-5-yl)­benzenesulfonamide (**L**) and its matched Rh­(III) and Ir­(III) half-sandwich complexes, [(η^5^-Cp*)­RhCl­(L)]­PF_6_ (**1**) and [(η^5^-Cp*)­IrCl­(L)]­PF_6_ (**2**), and to determine how coordination and metal identity influence their physicochemical and biological profiles. The compounds were characterized by CHNS elemental analysis, ESI-MS, FT-IR, NMR, TG, UV–vis spectroscopy, and single-crystal X-ray diffraction, and their solution stability and partition coefficients were evaluated. Their biological assessment included carbonic anhydrase inhibition, HSA interactions, an exploratory CT-DNA UV–vis experiment, antiproliferative activity across cancer and nonmalignant cell lines, and time-resolved analysis of cell-cycle distribution, apoptosis, caspase-3/7 activation, mitochondrial membrane potential, γH2AX signaling, and ROS generation in NALM-6 cells. This design was intended to identify metal-dependent differences in activity and cellular response while explicitly distinguishing temporal association from mechanistic causality.

## Experimental Section

### Materials and Physicochemical Measurements

*N*-Acetylsulfanilyl chloride (98%) (CAS: 121-60-8), carbonic anhydrase from bovine erythrocytes (CAS: 9001-03-0, C2624, ≥3.500 W-A units/mg protein), DNA solution from calf thymus (CAS: 73049-39-5), cisplatin, albumin from human serum (HSA, lyophilized powder, ≥97%) (CAS: 70024–90–7), NH_4_PF_6_ (99%) (CAS: 16941-11-0), acetazolamide (CAS: 59-66-5), 4-nitrophenyl acetate (CAS: 830-03-5), HEPES (≥99.5%) (CAS: 7365-45-9) were purchased from Sigma-Aldrich. 1,10-Phenanthrolin-5-amine (99%) (CAS: 54258-41-2), [CpIr­(μ-Cl)­Cl]_2_ (98%) (CAS: 12354-84-6) were purchased from AmBeed. [CpRh­(μ-Cl)­Cl]_2_ (98%) (CAS: 12354-85-7) was purchased from Angene. Anhydrous pyridine (CAS: 110-86-1) was purchased from Chempur. HCl 35–38% (CAS: 7647-01-0), CH_3_OH (CAS: 67-56-1), NaHCO_3_ (CAS: 144-55-8), acetone (CAS: 67-64-1), NaCl (CAS: 7647-14-5) were purchased from Stanlab. CH_2_Cl_2_ (CAS: 75-09-2), C_2_H_5_OH (CAS: 64-17-5), CH_3_CN (CAS: 75-05-8), N,N-dimethylformamide (DMF) (CAS: 68-12-2) were purchased from POCH-Basic. DMSO (CAS: 67-68-5) was purchased from Eurochem. 4-(2-Hydroxyethyl)-1-piperazinemethanesulfonic acid (10 mM HEPES, pH 7.2) buffer was prepared using Milli-Q water.

Elemental analysis (CHNS) was performed on an elementar vario micro cube analyzer. FT-IR spectra were recorded on a perkinElmer spectrum two spectrometer in the spectral range of 4000–400 cm^–1^. Mass spectra (MS) were recorded using a Bruker Daltonics (HCT Ultra) instrument. Analysis was performed on aqueous-methanolic solutions of the obtained compounds. ^1^H NMR and ^13^C NMR spectra were recorded at 25 °C in DMSO solutions using a Bruker AVANCE III spectrometer (^1^H 500 MHz, ^13^C 126 MHz). UV–vis spectroscopy measurements were performed on an evolution 300 instrument (Thermo Scientific, USA) in the spectral range of 200–800 nm. One cm thick quartz cuvettes were used. Thermogravimetric analysis (TG) was performed using a NETZSCH Jupiter STA 449 F3 thermogravimetric analyzer in the temperature range of 28–1000 °C, with a heating rate of 10 °C/min, in corundum crucibles and a nitrogen atmosphere. Analysis of the interaction with CA was performed using the VICTOR nivo multimode microplate reader. The results obtained from the plate reader were processed in GraphPad Prism 11. The results obtained using the above-mentioned equipment (spectra) were graphically processed using Origin software.

### Crystal Structure Determination

Single-crystal X-ray diffraction data for **L** were collected at 120.0(2) K on an IPDS 2T dual-beam diffractometer equipped with a GeniX 3D Mo HighFlux microfocus X-ray source (Mo Kα radiation, λ = 0.71069 Å, 50 kV, 1.0 mA). The crystal was cooled in a stream of dry nitrogen using a CryoStream-800 device. Data collection and reduction were performed using the X-Area 1.75 software package.^43^ The structure was solved by intrinsic phasing with SHELXT^44^ and refined by full-matrix least-squares on *F* ^2^ using SHELXL-2015 ^45^ within the Olex2 graphical interface.^46^ Single-crystal X-ray diffraction data for **1** and **2** were collected at 120 K on a STOE STADIVARI diffractometer equipped with a DECTRIS EIGER2 R 1 M CdTe detector and an Anton Paar Mo microfocus X-ray source (Mo Kα radiation, λ = 0.71073 Å). Data collection, integration, and scaling were carried out using the X-Area software package.^43^ An empirical absorption correction based on integration was applied during data reduction. The structures were solved by intrinsic phasing using SHELXT^44^ and refined by full-matrix least-squares on *F* ^2^ using SHELXL-2025/1 ^45^ implemented in Olex2 version 1.5.^46^ Molecular graphics were prepared with Diamond.^47^ For all structures, non-hydrogen atoms were refined anisotropically unless stated otherwise. Hydrogen atoms bonded to carbon and nitrogen atoms were placed in calculated positions and refined using a riding model with isotropic displacement parameters fixed at 1.2U_eq of the parent atom (1.5U_eq for methyl groups). In structure **1**, the hexafluorophosphate anion was refined as disordered over two orientations using a split-atom model with appropriate geometric and displacement-parameter restraints. Residual diffuse solvent electron density was treated using the solvent mask procedure implemented in Olex2.^46^ The refinement converged satisfactorily, and the final structural model is considered chemically and crystallographically reasonable. In the structure of **2**, a partially occupied solvent water molecule (O4) was refined isotropically with a fixed site occupancy factor of 0.50. Hydrogen atoms associated with this water molecule could not be located reliably in the difference Fourier map and were therefore omitted from the refinement. Inspection of the diffraction images indicated an additional scattering contribution that could not be satisfactorily modeled. No satisfactory twinning model could be established. Residual electron density near the iridium atom and adjacent carbon atoms is attributed to Fourier truncation effects associated with the heavy Ir atom and the additional unresolved scattering contribution. Despite these limitations, the refinement converged satisfactorily, and the final structural model is considered chemically and crystallographically reasonable.

Crystallographic data have been deposited with the cambridge crystallographic data centre (CCDC) under deposition numbers CCDC 2558558 (**L**), CCDC 2572068 (**1**), and CCDC 2572069 (**2**). These data are available free of charge via the CCDC web site: https://www.ccdc.cam.ac.uk/structures.

### Stability in Solution

The stability of the compounds in aqueous solution containing 2% DMSO was tested using UV–vis spectroscopy in the range of 200–800 nm at 0, 24, 48, and 72 h. Additionally, the stability of the tested compounds was evaluated in various acetonitrile/water ratios at 0 and 24 h.

### Determination of the n-Octanol/water Partition Coefficient (log *P*)

Calibration curves were prepared for all compounds tested. For compound **L**, octanol solutions with concentrations ranging from 5 to 30 μM were used to construct the calibration curves. For complexes **1** and **2**, only aqueous solutions with concentrations ranging from 5 to 35 μM were used to create calibration curves. The decision to prepare a calibration curve for **L** in octanol only and for complexes **1** and **2** in water was due to solubility issues. The UV spectra were recorded in the 200–400 nm range for **L** and 200–500 nm for complexes **1** and **2**. For each compound, the wavelength corresponding to the absorption maximum (λ_max_) was selected. For ligand **L**, this was 268 nm in octanol, whereas both complexes were determined in aqueous solution at 257 nm (**1**) and 260 nm (**2**). Based on the absorbance values recorded at specific wavelengths, calibration curves were constructed, and the corresponding molar absorption coefficients were determined. The calibration curves were subsequently used to determine the partition coefficient by the shake-flask method.^48^ For ligand **L**, 10 mL of its solution in octanol at a concentration of 25 μM was mixed with 10 mL of water in a pear-shaped separatory funnel. For complexes **1** and **2**, aqueous solutions of the compounds at the same concentration (25 μM) were prepared, and 10 mL of octanol was subsequently added. Intensive manual shaking was carried out for 30 min, with occasional degassing. The separator was then left on a stand overnight until phase separation was complete. The UV–vis spectra of both fractions were recorded in the 200–400 nm range. The data are presented as the mean ± standard deviation (SD) from three replicates. The concentrations of the compounds in each phase were calculated using the previously determined molar absorption coefficients and the Lambert–Beer law. Concentrations were calculated from the differences, subtracting the observed absorbance from the initial absorbance to determine the concentration of the second phase. The following formula was used to calculate the log *P* value (eq 1)­

1 $$\log P=\log\frac{c_{octanol}}{c_{water}}$$

where *c*_octanol_ is the concentration of the tested compound in the octanol layer, and *c*_water_ is the concentration of the compound in the water layer.

### Carbonic Anhydrase Inhibition Studies

Absorbance was measured at 400 nm at 1 min intervals for 30 min. Each experiment was performed twice, with three technical replicates in each run. Bovine erythrocyte carbonic anhydrase was used as the enzyme source. This commercially available product consists mainly of the CA II isoform, with a minor presence of CA I and CA IV. The enzymatic activity was evaluated using 4-nitrophenyl acetate as the substrate, which is hydrolyzed to form p-nitrophenolate. Before addition of substrate, the enzyme-compound mixtures were preincubated at 37 °C for 15 min. The enzyme concentration was 0.45 μM, calculated based on the weighed amount of lyophilized enzyme rather than determined by spectrophotometric measurement. The substrate solution was added immediately before starting the kinetic measurements. To determine the *K*_m_ value for 4-nitrophenyl acetate, substrate solutions were prepared in EtOH at 8 different concentrations. The final concentrations were: 13.11, 6.56, 3.28, 1.64, 0.82, 0.41, 0.20, and 0.10 mM. Higher substrate concentrations were not tested due to precipitation observed under the assay conditions, which could interfere with absorbance measurements. For each substrate concentration, the initial reaction velocity was determined from the linear increase in absorbance over time (Δ*A*/min). The initial velocities were fitted to the Michaelis–Menten eq 2 using nonlinear regression

2 $$V=V_{\max}\cdot \frac{\left[S\right]}{\left[S\right]+K_{M}}$$

where *V* is the reaction rate, [*S*] is the substrate concentration, *V*_max_ is the maximum reaction rate, and *K*_m_ is the Michaelis constant, which determines the enzyme’s affinity for the substrate.

To determine the IC_50_ values, measurements were conducted using solutions of complexes **1** and **2** in DMSO. The final DMSO content at the time of measurement was 6.67% by volume. The remainder of the mixture consisted of HEPES buffer (pH 7.2) with 40 mM NaCl and EtOH. Twelve concentration variants were prepared for each complex. For complex **1**: 780, 720, 660, 600, 540, 480, 420, 360, 300, 240, 180, 120 μM and for complex **2**: 840, 780, 720, 660, 600, 540, 480, 420, 360, 300, 240, and 180 μM. DMSO was necessary because the compounds were not soluble in water. **L** was also preliminarily tested to determine a potential IC_50_ at a later stage, using the following concentrations: 180, 240, 300, 360, 420, 480, 540, and 600 μM. The final substrate concentration was 546 μM. The IC_50_ values were determined using nonlinear regression with a variable-slope inhibition model. An attempt was made to determine the inhibition mechanism of the complexes under study.

### Interaction with CT-DNA

A solution of DNA sodium salt from calf thymus (CT-DNA) was prepared in HEPES buffer (pH 7.2) with 40 mM NaCl. Its concentration was determined based on the absorbance at a wavelength of 260 nm, using a molar absorptivity of 6600 M^–1^ cm^–1^. Solutions of the ligand and complexes were prepared at a concentration of 20 μM in a 1% DMSO/HEPES buffer mixture. The concentration used was constant. Various concentrations of CT-DNA (0–25 μM) were used in HEPES buffer. The spectra were recorded after 72 h of equilibration at room temperature.^49^

### Interaction with HSA

An HSA solution was prepared in HEPES buffer (pH 7.2) with 40 mM NaCl. The concentration was calculated from absorbance at 280 nm using a molar absorptivity of 36600 M^-1^ cm^-1^. The HSA concentration was kept constant at 8 μM.^50^ Various concentrations of ligand and complexes were used (0–10 μM) in a 1% DMSO/HEPES buffer mixture. Spectra were recorded after 72 h of equilibration at room temperature. The equilibrium constant of the bond (*K*_b_) was determined using eq 3 by monitoring changes in absorbance^42^

3 $$\frac{1}{A_{obs}-A_{0}}=\frac{1}{A_{c}-A_{0}}+\frac{1}{K_{b}\left(A_{c}-A_{0}\right)\left[C\right]}$$

where *A*_obs_ is the observed absorbance of solutions containing different concentrations of the compounds being analyzed [C], and *A*_0_ and *A*_c_ are the absorbances of the HSA solution and the HSA solution containing the compound being analyzed at 280 nm.

### Cell Culture

Five human cell lines were employed to assess the antiproliferative effects and selectivity profile of the tested compounds. The panel included four cancer-derived lines of distinct histological origin: MCF-7 (breast adenocarcinoma, ATCC HTB-22), A-549 (nonsmall cell lung carcinoma, ATCC CCL-185), HCT116 (colorectal carcinoma, ATCC CCL-247), and NALM-6 (acute lymphoblastic leukemia, DSMZ ACC 128). To evaluate off-target cytotoxicity, HEK-293 cells (human embryonic kidney, ATCC CRL-1573) were used as a model of nonmalignant, proliferating cells. MCF-7, A-549, and NALM-6 cells were maintained in RPMI-1640 medium, HCT116 cells in McCoy’s 5A medium, and HEK-293 cells in dulbecco’s modified eagle medium (DMEM). All media were supplemented with 10% fetal bovine serum (FBS), 2 mM l-glutamine, 100 U/mL penicillin, and 100 μg/mL streptomycin. Cells were cultured at 37 °C in a humidified atmosphere with 5% CO_2_, except HEK-293 cells, which were maintained under 10% CO_2_ to ensure optimal growth. Subculturing was carried out at 70–80% confluence using standard trypsinization protocols (or gentle centrifugation for suspension cells). All cultures were routinely tested for Mycoplasma contamination using PCR-based detection methods.

### Cell Viability Assay

Cell viability was evaluated using the MTT assay (3-(4,5-dimethylthiazol-2-yl)-2,5-diphenyltetrazolium bromide; Sigma-Aldrich), a colorimetric method based on the enzymatic reduction of MTT to insoluble formazan by metabolically active cells. Cells were seeded into 96-well plates at appropriate densities and exposed to a range of tested compound concentrations for 72 h. DMSO-treated cells served as a negative control, with the final solvent concentration not exceeding 0.5%. At the end of the treatment period, add MTT solution to each well to a final concentration of 0.4 mg/mL, then incubate the plates for an additional 2–3 h at 37 °C. The resulting formazan crystals were solubilized in 100 μL of DMSO per well, and absorbance was recorded at 450 nm using an ASYS UVM340 microplate reader. IC_50_ values (the concentration required to inhibit 50% of cell viability) were derived from nonlinear regression analysis of dose–response curves using GraphPad Prism 9 software. All treatments were performed in technical triplicate, and the entire experiment was independently repeated at least three times. Data are presented as mean ± standard deviation (SD) from three biological replicates.

### Cell Cycle Analysis

NALM-6 cells were seeded into culture plates and allowed to recover under standard conditions (37 °C, 5% CO_2_). Cells were then treated for 24 or 48 h with the tested compounds at concentrations corresponding to their respective IC_90_ values, determined previously via viability assays. Following treatment, cells were fixed in 75% ice-cold ethanol and stored at −20 °C overnight. Prior to analysis, samples were washed twice with phosphate-buffered saline (PBS), centrifuged, and stained in the dark for 30 min at room temperature with a solution containing propidium iodide (20 μg/mL) and RNase A (50 μg/mL; both from Sigma-Aldrich). DNA content was assessed using a Guava easyCyte 8 flow cytometer (Merck Millipore), and cell-cycle distribution was quantified using FlowJo v10 software (BD Life Sciences).

### Apoptosis Assessment

To evaluate apoptosis, NALM-6 cells were seeded into tissue culture plates and incubated overnight under standard conditions (37 °C, 5% CO_2_) to allow recovery. The following day, cells were treated with the test compounds at IC_90_ concentrations for either 24 or 48 h. After treatment, cells were collected by centrifugation, washed twice with phosphate-buffered saline (PBS), and stained according to the manufacturer’s protocols. Phosphatidylserine externalization—a hallmark of early apoptosis—was detected using an Annexin V-FITC conjugate (thermo fisher scientific, #A13199). Cells were resuspended in Annexin V binding buffer, and Annexin V-FITC was added at 5 μL per 100 μL of cell suspension. Samples were incubated for 15 min at room temperature in the dark, then 7-AAD was added to a final concentration of 1 μg/mL.

Caspase-3/7 activation was assessed using the CellEvent Caspase-3/7 green flow cytometry assay Kit (thermo fisher scientific, #C10427), which employs a fluorogenic substrate that becomes fluorescent upon cleavage by active caspase-3/7. CellEvent Caspase-3/7 green detection reagent was added to a final concentration of 500 nM, and samples were incubated for 30 min at 37 °C in the dark. 7-AAD was used at a final concentration of 1 μg/mL to exclude cells with compromised plasma membranes. Stained cells were analyzed on a Guava easyCyte 8 flow cytometer (Merck Millipore), and data were processed using FlowJo v10 software (BD Life Sciences). Fluorescence compensation was established using unstained and single-stained controls and applied consistently to all samples acquired using the same instrument settings.

### Evaluation of DNA Double-Strand Breaks (γH2AX Assay)

To evaluate DNA double-strand break (DSB) induction, NALM-6 cells were seeded into tissue culture plates and allowed to recover overnight under standard conditions. Cells were then treated with the test compounds at IC_90_ concentrations for 24 or 48 h. Following treatment, cells were harvested by centrifugation, fixed in 75% ice-cold ethanol, and stored at −20 °C until analysis. For staining, cells were rehydrated on ice in PBS for 5 min, then permeabilized with 0.2% Triton X-100 in PBS for 15 min at room temperature. Intracellular γH2AX levels—a marker of DSBs—were detected using an Alexa Fluor 488-conjugated antiphospho-Histone H2AX (Ser139) antibody (#613406, BioLegend) diluted 1:200 in PBS. Cells were incubated at 37 °C for 90 min in the dark. After antibody staining, cells were washed with PBS and counterstained with propidium iodide (20 μg/mL) and RNase A (50 μg/mL) for 30 min. Fluorescence signals were acquired using a Guava easyCyte 8 flow cytometer (Merck Millipore), and data were analyzed using FlowJo v10 software (BD Life Sciences). Fluorescence compensation was established using unstained and single-stained controls and applied consistently to all samples acquired using the same instrument settings.

### Assessment of Mitochondrial Membrane Potential

To evaluate mitochondrial membrane potential (ΔΨ*m*), NALM-6 cells were treated with the tested compounds at their respective IC_90_ concentrations for 6 or 24 h. Following treatment, the culture medium was replaced with fresh medium supplemented with JC-1 dye (5 μg/mL; Sigma-Aldrich), and cells were incubated in the dark at 37 °C for 20 min. After staining, cells were washed twice with phosphate-buffered saline (PBS) and analyzed by flow cytometry using a Guava easyCyte 8 cytometer (Merck Millipore). Data were processed with FlowJo v10 software (BD Life Sciences), and the degree of mitochondrial depolarization was quantified based on JC-1 monomer fluorescence. FCCP (carbonyl cyanide 4-(trifluoromethoxy)­phenylhydrazone), a potent mitochondrial uncoupler, was used at 100 μM as a positive control to induce maximal loss of ΔΨ*m*. Fluorescence compensation was established using unstained and single-stained controls and applied consistently to all samples acquired using the same instrument settings.

### Assessment of Intracellular Reactive Oxygen Species

To quantify reactive oxygen species (ROS) generation, NALM-6 cells were treated with the tested compounds at their respective IC_90_ concentrations for 1, 3, 6, or 24 h. Thirty minutes before the end of each treatment period, 1 μM CM-H_2_DCFDA (Thermo Fisher Scientific, #C6827), a cell-permeable ROS-sensitive fluorescent probe, was added to the culture medium. Hydrogen peroxide (H_2_O_2_, 250 μM) was used as a reference control to confirm probe responsiveness. After completion of treatment, cells were collected by centrifugation, washed with PBS, and stained with 7-aminoactinomycin D (7-AAD; Sigma-Aldrich) to exclude dead cells from analysis. ROS-associated fluorescence was measured by flow cytometry using a Guava easyCyte 8 cytometer (Merck Millipore), and data were analyzed using FlowJo v10 software (BD Life Sciences). The percentage of ROS-positive cells was quantified relative to vehicle-treated controls. Fluorescence compensation was established using unstained and single-stained controls and applied consistently to all samples acquired using the same instrument settings.

### Synthesis of the Ligand and Complexes

#### Preparation of 4-Amino-*N*-(1,10-phenanthrolin-5-yl)­benzenesulfonamide (**L**)

A Bunsen-burner-dried 250 mL round-bottom flask was charged with 1,10-phenanthrolin-5-amine (3.00 g, 15.37 mmol, 1 equiv) and dissolved in 105 mL of anhydrous pyridine at 100 °C. The resulting yellow solution was cooled down to 40 °C, and then 4-acetamidobenzene-1-sulfonyl chloride (3.95 g, 16.90 mmol, 1.1 equiv), freshly recrystallized from CHCl_3_, was added in one portion, and the red solution was stirred at room temperature overnight. Next, the pyridine was removed by rotatory evaporation (25 mbar/50 °C for 2 h) to obtain approximately 5.50 g of an orange-red hygroscopic powder, which was used in the next step without purification. In the second step, 38 mL of MeOH, 6.25 mL of concentrated HCl, and 6.25 mL of water were added to a 250 mL round-bottom flask containing the compound from the previous step (approximately 5.50 g). The resulting deep-red solution was refluxed for 1 h, then cooled to room temperature. Most of the MeOH was removed using a rotary evaporator, then the residue was diluted with 100 mL of distilled water and neutralized to pH 7 with saturated aqueous NaHCO_3_ solution. The resulting yellow solid was filtered off, washed with distilled water, and dried in air. The obtained crude product (4.09 g) was purified by quickly dissolving it in 41 mL of hot MeOH (1 mL per 100 mg of product) and letting the solution cool down to room temperature. The resulting light-yellow to brown crystals were filtered off, washed with a small amount of ice-cold MeOH, and dried at 50 °C to remove MeOH. The pure product was obtained with 40% yield over two steps (2.14 g, 6.11 mmol). The yield can be improved by evaporating the filtrate and recrystallizing the resulting solid. Anal. Calcd (%) for C_18_H_14_N_4_O_2_S: C, 61.70; H, 4.03; N, 15.99; S, 9.15. Found: C, 61.51; H, 4.05; N, 16.00; S, 9.29. ESI-MS *m*/*z*: exp./calcd (ion): 348.90/349.39 ([L – H]^−^); 351.10/351.39 ([L + H]^+^); 723.10/723.77 ([2L + Na]^+^). FT-IR (ATR, cm^–1^): 3453 (m), 3367 (m), 3335 (m) (−NH_2_), 3225 (m) (NH), 1644 (s), 1592 (s) (C=C ring), 1504 (m), 1419 (m) (C=N ring), 1307 (m), 1145 (m) (SO_2_). ^1^H NMR (500 MHz, DMSO-*d*_6_, δ ppm): 10.11 (s, 1H, N**H**_7_); 9.05 (dd, *J* = 4.3, 1.6 Hz, 1H, C**H**_10_); 9.03 (dd, *J* = 4.3, 1.7 Hz, C**H**_11_); 8.57 (dd, *J* = 8.4, 1.7 Hz, 1H, C**H**_8_); 8.39 (dd, *J* = 8.1, 1.7 Hz, C**H**_13_); 7.72 (dd, *J* = 5.2, 4.3 Hz, 1H, C**H**_9_); 7.70 (dd, *J* = 5.5, 4.2 Hz, 1H, C**H**_12_); 7.65 (s, 1H, C**H**_14_); 7.38–7.32 (m, 2H, C**H**_5/6_); 6.50–6.44 (m, 2H, C**H**_3/4_); 5.96 (bs, 2H, N**H**_1/2_). ^13^C NMR (126 MHz, DMSO-d_6_, δ ppm): 124.09 (C_1_); 112.97 (C_2/3_); 129.29 (C_4/5_); 153.38 (C_6_); 132.09 (C_7_); 126.09 (C_8_); 132.54 (C_9_); 124.68 (C_10_); 150.37 (C_11_); 146.37 (C_12_); 144.45 (C_13_); 150.07 (C_14_); 123.19 (C_15_); 136.39 (C_16_); 128.25 (C_17_); 121.56 (C_18_).

#### Preparation of [(η^5^-Cp*)­RhCl­(L)]­PF_6_ (**1**)

The substrate [Cp*Rh­(μ-Cl)­Cl]_2_ (0.1 mmol, 0.0638 g) was dissolved in an EtOH/acetone mixture of 18 mL:9 mL. The ligand (**L**) (0.2 mmol, 0.0698 g), dissolved in an EtOH/acetone mixture (12 mL:6 mL), was added dropwise. The solution was stirred for an hour at 70 °C. Then, NH_4_PF_6_ (0.2 mmol, 0.0333 g), dissolved in 5 mL EtOH, was added dropwise. The solution was stirred and heated to reflux for 8 h. It was then filtered and left to crystallize at room temperature. After 1 day, an orange precipitate was formed, which was filtered off and dried in a vacuum chamber. The product was obtained in 84% yield. Anal. Calcd (%) for C_28_H_29_N_4_O_2_SClPF_6_Rh: C, 43.74; H, 3.80; N, 7.29; S, 4.17. Found: C, 42.80; H, 4.04; N, 7.13; S, 3.99. ESI-MS *m*/*z*: exp./calcd (ion): 144.7/144.96 ([PF_6_]^−^); 349.0/349.39 ([L – H]^−^); 623.1/623.98 ([(η^5^-Cp*)­RhCl­(**L**)]^+^); 767.0/767.94 {([(η^5^-Cp*)­RhCl­(**L**)]­PF_6_)^−^}. FT-IR (ATR, cm^–1^): 3488 (m), 3373 (m) (−NH_2_), 3229 (m) (NH), 1631 (s), 1595 (s) (C=C ring), 1468 (m), 1428 (m) (C=N ring), 1320 (m), 1151 (m) (SO_2_), 832 (vs) (PF_6_^–^). ^1^H NMR (500 MHz, DMSO-*d*_6_, δ ppm): 10.70 (s, 1H, N**H**_7_), 9.38 (dd, *J* = 5.2, 1.2 Hz, 1H, C**H**_10_), 9.27 (dd, *J* = 5.1, 1.3 Hz, 1H, C**H**_11_), 9.02 (dd, *J* = 8.4, 1.2 Hz, 1H, C**H**_8_), 8.87 (dd, *J* = 8.3, 1.3 Hz, 1H, C**H**_13_), 8.19 (dd, *J* = 8.5, 5.1 Hz, 1H, C**H**_9_), 8.12 (dd, *J* = 8.3, 5.1 Hz, 1H, C**H**_12_), 7.90 (s, 1H, C**H**_14_), 7.53–7.46 (m, 2H, C**H**_5/6_), 6.56–6.49 (m, 2H, C**H**_3/4_), 6.06 (s, 2H, N**H**_1/2_), 1.72 (s, 15H, C**H**_15/29_). ^13^C NMR (126 MHz, DMSO-*d*_6_, δ ppm): 124.13 (C_1_); 113.11 (C_2/3_); 129.52 (C_4/5_); 153.70 (C_6_); 134.45 (C_7_); 127.36 (C_8_); 135.73 (C_9_); 126.89 (C_10_); 153.11 (C_11_); 145.70 (C_12_); 142.96 (C_13_); 151.86 (C_14_); 127.60 (C_15_); 138.85 (C_16_); 129.99 (C_17_); 118.57 (C_18_); 97.46 (C_19/23_); 8.99 (C_24/28_).

#### Preparation of [(η^5^-Cp*)­IrCl­(L)]­PF_6_ (**2**)

Complex (**2**) was prepared as described for (**1**). In this case, the precursor [Cp*Ir­(μ-Cl)­Cl]_2_ (0.1 mmol, 0.0799 g), ligand (0.2 mmol, 0.0723 g), and NH_4_PF_6_ (0.2 mmol, 0.0341 g) were used. The product was obtained as a yellow solid with 90% yield. Anal. Calcd (%) for C_28_H_29_N_4_O_2_SClPF_6_Ir: C, 39.18; H, 3.41; N, 6.53; S, 3.74. Found: C, 38.73; H, 3.21; N, 6.61; S, 3.22. ESI-MS *m*/*z*: exp./calcd (ion): 144.8/144.96 ([PF_6_]^−^); 713.2/713.29 ([(η^5^-Cp*)­IrCl­(**L**)]^+^); 855.8/857.25 {([(η^5^-Cp*)­IrCl­(**L**)]­PF_6_)^−^}. FT-IR (ATR, cm^–1^): 3488 (m), 3374 (m) (−NH_2_), 3230 (m) (NH), 1633 (s), 1595 (s) (C=C ring), 1470 (m), 1428 (m) (C=N ring), 1321 (m), 1152 (m) (SO_2_), 834 (vs) (PF_6_^–^). ^1^H NMR (500 MHz, DMSO-d_6_, δ ppm): 10.73 (s, 1H, N**H**_7_), 9.36 (dd, *J* = 5.3, 1.1 Hz, 1H, C**H**_10_), 9.25 (d, *J* = 5.0 Hz, 1H, C**H**_11_), 9.02 (dd, *J* = 8.5, 1.1 Hz, 1H, C**H**_8_), 8.88 (d, *J* = 8.2 Hz, 1H, C**H**_13_), 8.18 (dd, *J* = 8.5, 5.2 Hz, 1H, C**H**_9_), 8.10 (dd, *J* = 8.1, 5.1 Hz, 1H, C**H**_12_), 7.96 (d, *J* = 2.2 Hz, 1H, C**H**_14_), 7.55–7.48 (m, 2H, C**H**_5/6_), 6.56–6.50 (m, 2H, C**H**_3/4_), 6.06 (s, 2H, N**H**_1/2_), 1.70 (s, 15H, C**H**_15/29_). ^13^C NMR (126 MHz, DMSO-*d*_6_, δ ppm): 124.13 (C_1_); 113.12 (C_2/3_); 129.55 (C_4/5_); 153.72 (C_6_); 134.92 (C_7_); 127.63 (C_8_); 135.81 (C_9_); 127.36 (C_10_); 152.83 (C_11_); 147.15 (C_12_); 144.22 (C_13_); 151.40 (C_14_); 128.02 (C_15_); 138.92 (C_16_); 130.31 (C_17_); 118.03 (C_18_); 89.62 (C_19/23_); 8.67 (C_24/28_).

## Results and Discussion

### Synthesis

As a result of a two-step reaction, 4-amino-*N*-(1,10-phenanthrolin-5-yl)­benzenesulfonamide (**L**) was obtained. In the first step, 4-acetamidobenzenesulfonyl chloride condensed with 1,10-phenanthrolin-5-amine. In the second step, the acetyl protecting group was hydrolyzed (Scheme 1). The product was recrystallized from methanol, yielding pale yellow monocrystals. It was generally highly soluble in common solvents, apart from water. The coordination compounds were prepared using rhodium­(III) and iridium­(III) precursors, dimers of pentamethylcyclopentadienyl chlorides, a previously synthesized sulfonamide ligand, and ammonium hexafluorophosphate, as shown in Scheme 1. The reaction yielded both complexes as precipitates. They were then recrystallized from a water/acetone mixture, yielding yellow monocrystals suitable for SC-XRD studies. Both complexes were generally highly soluble in alcohols, water, DMSO, DMF, acetone, and CH_3_CN. In the MS mass spectra (Figures S1–S3), peaks were observed corresponding to the masses of the resulting compounds in the case of **L** [M + H]^+^, and in the case of complexes **1** and **2** [(η^5^-Cp*)­Rh/IrCl­(**L**)]^+^. Furthermore, the compounds obtained were characterized using ^1^H, ^13^C, HSQC, and COSY NMR **(** Figures S4–S21 **)**. Table S1 presents the carbon and hydrogen atom numbering schemes, along with the assigned shift values. The FT-IR spectra are shown in Figure S22.

**Scheme 1:**
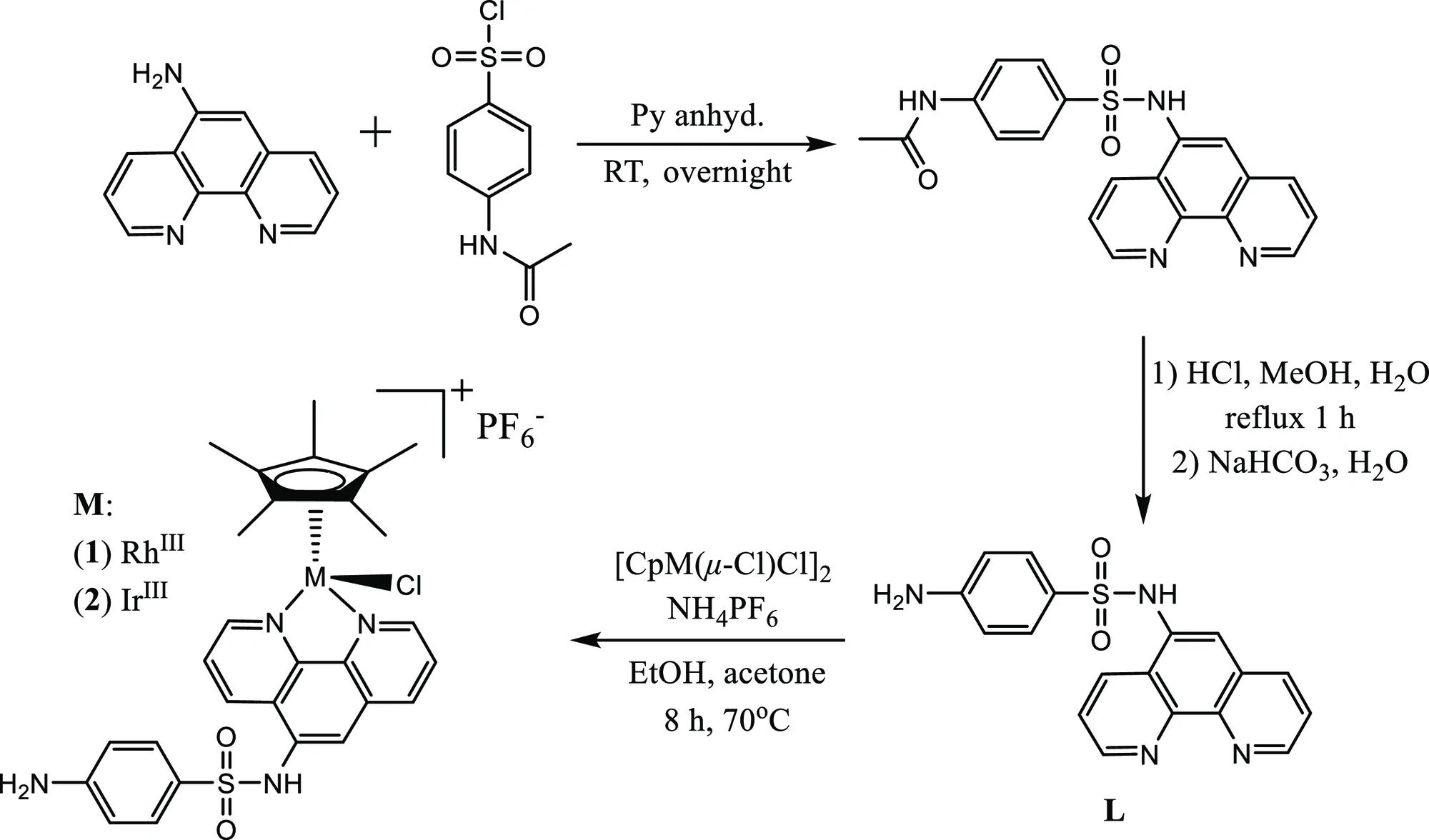
Schematic Diagram of the Synthesis of a New Sulfonamide Derivative (**L**), Followed by Two Coordination Compounds of Rh­(III) (**1**) and Ir­(III) (**2**)

### Crystal Structure of 4-Amino-*N*-(1,10-phenanthrolin-5-yl)­benzenesulfonamide (**L**)

The results of the X-ray diffraction experiments are presented in Table 1. 4-Amino-N-(1,10-phenanthrolin-5-yl)­benzenesulfonamide (**L**) crystallizes in the monoclinic space group *P*2_1_/*n* with one molecule per asymmetric unit and four molecules per unit cell. The molecular structure of **L** is shown in Figure 1a, and the bond lengths and angles are shown in Table S2. A 4-aminophenyl group forms the S-site of the sulfonamide molecule, while the N-site of the molecule consists of a 1,10-phenanthrolin-5-yl substituent. The S=O bond lengths are 1.434(3) and 1.438(7) Å, with S1–N2 and S1–C1 bond lengths of 1.650(2) and 1.742(9) Å, respectively. These values are comparable with previously reported data^42,51,52^ and are generally observed in sulfonamides. The τ_4_ descriptor defines the continuous shape measure of a four-coordinate center transitioning between perfect square-planar (τ_4_ = 0.00) and perfect tetrahedral geometries (τ_4_ = 1.00).^53^ A value of 0.94 for a sulfonamide group in **L** indicates that its 4-fold coordination is highly regular but slightly flattened or stretched from a perfect tetrahedron. Arylsulfonamide conformation depends on the mobility of the (C–S–N–C) bridge connecting its two aromatic rings: Ph1 (C1–C2–C3–C4–C5–C6) and Ph2 (C7–C8–C9–C13–C14–C18). Three single bonds give the molecule high flexibility, allowing it to adopt V-shaped or twisted conformations. These rotations allow the molecule to orient itself for protein binding or crystal packing. The four parameters define the conformation and flexibility of the **L** sulfonamide molecule:^54,55^ τ_1_ (<C2–C1–S1–N2) measures the orientation of the −SO_2_– group relative to the plane of the first phenyl ring (Ph1). It dictates the steric positioning of the sulfonyl group; τ_2_ (<C1–S1–N2–C7) describes the rotational mobility and flexibility of the sulfur–nitrogen (S–N) bond, which acts as a primary pivot point connecting the aromatic components; τ_3_ (<S1–N2–C7–C6) characterizes the positioning and rotation of the second phenyl ring of phenanthroline moiety (Ph2) with respect to the −NH– bridging group; < Ph1-Ph2 evaluates the overall 3D shape by calculating the dihedral angle between the planes of the two aromatic rings. The **L** sulfonamide molecule is Z-shaped with a dihedral angle between the two aromatic rings of 16.69° (Figure 1b). The torsion angles (τ_1_, τ_2,_ and τ_3_) bear high positive values, which suggests a (+)-anticlinal conformation. The most important torsion angle (τ_2_) allows the mobility of the −SO_2_NH– bridge and thus defines well the conformation of the whole molecule. Hence, the τ_2_ value of 102.23°, which is in the range of 90° to 150°^56^, consequently indicates the adoption of a (+)-anticlinal conformation by 4-amino-*N*-(1,10-phenanthrolin-5-yl)­benzenesulfonamide molecule in the solid state (Figure 1b).

**Table 1:** Crystallographic Data and Structure Refinement Details for Compounds **L**, **1**, and **2**

compound reference	**L**	**1**	**2**
empirical formula	C_18_H_14_N_4_O_2_S	C_31_H_35_ClF_6_N_4_O_3_PSRh	C_62_H_70_Cl_2_F_12_N_8_O_7_P_2_S_2_Ir_2_
*M*_r_ */*g mol^–1^	350.39	827.02	1848.62
temperature/K	120 (2)	120 (2)	120 (2)
wavelength/Å	1.54186	0.71073	0.71073
crystal system	monoclinic	triclinic	triclinic
space group	*P*2_1_/*n*	*P*1̅	*P*1̅
*a*/Å	9.2744(17)	8.4336 (3)	8.4316 (7)
*b*/Å	14.235(2)	14.5359 (6)	14.5709 (13)
*c*/Å	11.800(2)	16.3922 (7)	16.4238 (16)
α/°	90	112.634 (3)	112.705 (7)
β/°	104.029(13)	94.679 (3)	95.015 (7)
γ/°	90	101.322 (3)	101.378 (7)
*V*/Å^3^	1511.4(5)	1791.03 (13)	1794.7 (3)
Z	4	2	1
calculated density/Mg m^–3^	1.540	1.534	1.710
μ/mm^–1^	2.09	0.734	3.967
*F*(000)	728	840	912
*θ* range/°	4.958 to 64.930	2.466 to 25.100	2.467 to 29.631
limiting indices	–10 ≤ *h* ≤ 9	–10 ≤ *h* ≤ 10	–11 ≤ *h* ≤ 11
	–16 ≤ *k* ≤ 16	–17 ≤ *k* ≤ 17	–19 ≤ *k* ≤ 19
	–13 ≤ *l* ≤ 13	–19 ≤ *l* ≤ 19	–22 ≤ *l* ≤ 22
reflections collected/unique	6432/2520 [*R*_(int)_ = 0.0452]	55,886/6375 [*R*_(int)_ = 0.0375]	31,696/9191 [*R*_(int)_ = 0.0360]
completeness to *θ*_max_/%	98.2	100	90.8
refinement method	full-matrix least-squares on *F* ^2^	full-matrix least-squares on *F* ^2^	full-matrix least-squares on F^2^
data/restraints/parameters	2520/0/227	6375/225/461	9191/0/444
goodness-of-fit on *F* ^2^	1.069	1.951	1.069
final *R* indices [*I* > 2sigma(*I*)]	*R*_1_ = 0.0713 *wR*_2_ = 0.2011	*R*_1_ = 0.1325 *wR*_2_ = 0.3439	*R*_1_ = 0.0896 *wR*_2_ = 0.2450
*R* indices (all data)	*R*_1_ = 0.0743 *wR*_2_ = 0.2074	*R*_1_ = 0.1399 *wR*_2_ = 0.3974	*R*_1_ = 0.1047 *wR*_2_ = 0.2810
largest diff. peak and hole/eÅ^–3^	0.669; −1.019	3.479; −0.901	5.072; −4.377
CCDC number	2558558	2572068	2572069

**Figure 1:**
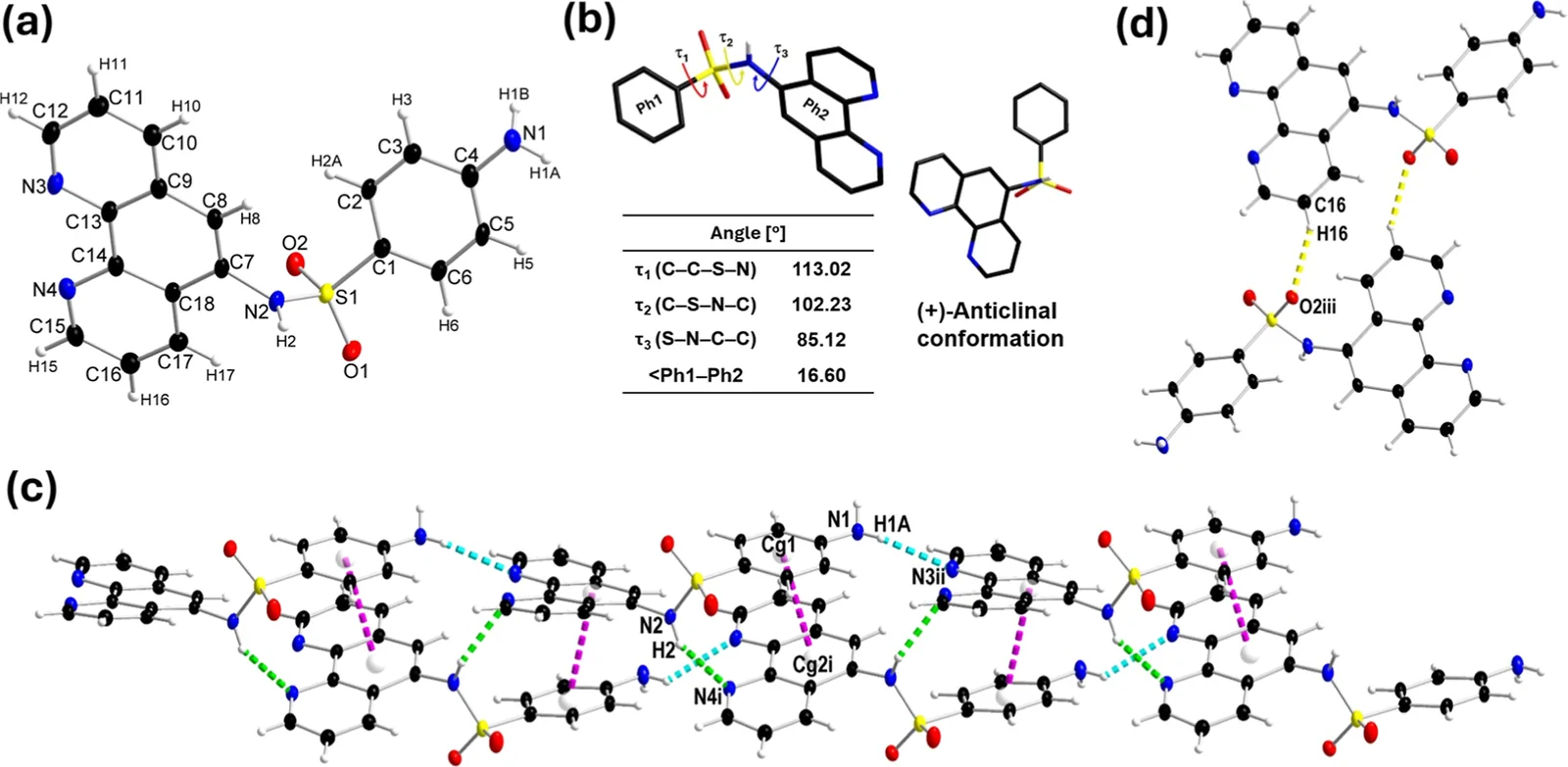
X-ray molecular structure of **L** sulfonamide with atom numbering scheme (a); selected X-ray geometrical parameters describing molecular conformational state in the crystal lattice of **L** (b); Geometry of noncovalent intermolecular interactions of **L**: (c) N–H···N (blue and green dashed) and π···π (purple dashed), (d) C–H···O (yellow dashed) [Symmetry codes: (i) 0.5 + *x*, 1.5 – *y*, 0.5 + *z*; (ii) 1 + *x*, *y*, 1 + *z*; (iii) −*x*, 1 – *y*, 1 – *z*.].

The crystal packing of **L** molecules results especially from the combination of H-bonds and π···π stacking interactions (Figure 1c,d; Table S3).^57^ The crystal structure is mainly stabilized by N–H···N hydrogen bonds formed between N–H donor of amine or sulfonamide group and phenanthroline N-acceptors (Figure 1c). These interactions form themselves 1D chains along ab plane, which are supported by π···π intertactions between S-phenyl and N-phenanthroline rings with intercentroid distance of 3.608(2) Å (Figure 1c). Further stabilization is provided by weaker C–H···O hydrogen bonds forming centrosymmetric dimeric units between phenanthroline C–H groups and sulfonamide O-acceptors of neighboring molecules (Figure 1d).

### Crystal Structures of Half-Sandwich Rhodium (**1**) and Iridium (**2**) Complexes

Both complexes **1** and **2** crystallize in the triclinic space group *P*-1. The basic unit consists of monocationic [(η^5^-Cp*)­MCl­(**L**)]^+^ species (where *M* = Rh­(III) (**1**) or Ir­(III) (**2**)), PF_6_^–^ anion and acetone molecule. In the structure of Ir­(III) complex (**2**) there is an additional water molecule (Figure 2). The molecular structure is described as a three-legged piano-stool geometry, where pentamethylcyclopentadienyl anion (Cp*) acts as a tridentate *fac*-donor, forming the “seat” of the piano stool, phenanthrolinesulfonamide ligand (**L**) binds in a bidentate N ∩ N fashion to the metal center and chloride anion (Cl^–^) acts as a terminal monodentate ligand. While the three legs (two nitrogen atoms and one chloride) visually suggest a pseudosquare-pyramidal or four-legged arrangement, the Cp* ligand in fact occupies three facial coordination sites, resulting in an overall distorted octahedral geometry around the metal center. The (+)-anticlinal conformation of the ligand is retained in the complexes. The Rh­(III) and Ir­(III) metal ions are coordinated by the η^5^-Cp* ligand with metal-centroid distances of 1.798(2) and 1.773(5) Å for **1** and **2**, respectively. The Ir/Rh–N and Ir/Rh–Cl bond lengths (Table 2) are in a typical range for the bonds in similar iridium and rhodium complexes reported previously.^42^ The crystal packing is mainly stabilized by a rich net of hydrogen bonds formed by N–H donors from amine and sulfonamide groups and F- or O-acceptors from PF_6_^–^, acetone, or H_2_O species (Figure S23).

**Figure 2:**
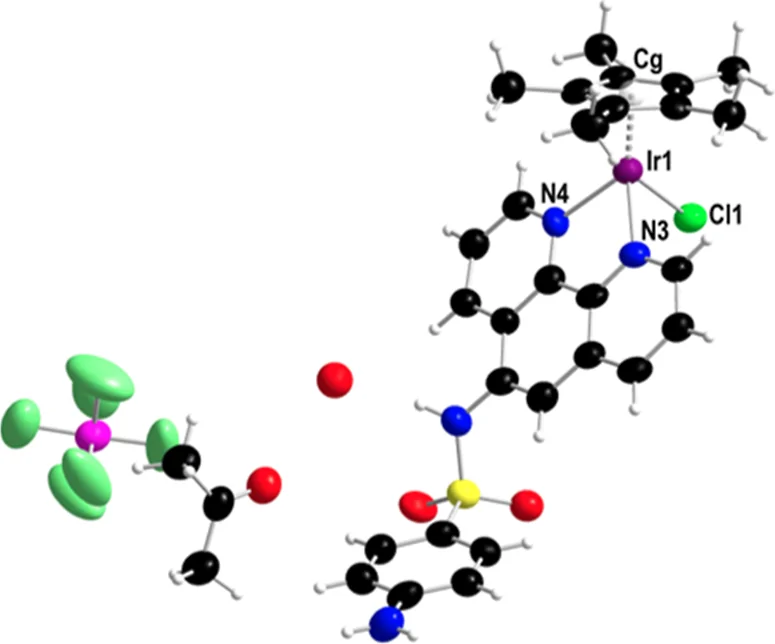
X-ray molecular structure of [(η^5^-Cp*)­IrCl­(**L**)]­PF_6_·(CH_3_)_2_CO·H_2_O complex (**2**) with the atom numbering scheme. The ellipsoids of all non-hydrogen atoms are shown at the 50% probability level.

**Table 2:** Selected Bond Lengths (Å) and Angles (deg) for Complexes **1** and **2**.^a^

1		2	
Bond Lengths (Å)			
Rh1–Cg	1.798(2)	Ir1–Cg	1.773(5)
Rh1–N3	2.138(6)	Ir1–N3	2.111(9)
Rh1–N4	2.168(6)	Ir1–N4	2.104(8)
Rh1–Cl1	2.394(2)	Ir1–Cl1	2.399(3)
Angles (deg)			
N3–Rh1–N4	76.5(2)	N3–Ir1–N4	76.9(4)
N3–Rh1–Cl1	87.7(2)	N3–Ir1–Cl1	85.6(3)
N4–Rh1–Cl1	87.6(2)	N4–Ir1–Cl1	86.2(3)

a The numbering of atoms is shown in Figure 2.

### FT-IR Spectroscopy

The FT-IR spectra are shown in Figure S22. In the higher-wavenumber range of the **L** spectra, absorption bands appear at 3453, 3367, and 3335 cm^–1^, corresponding to vibrations of the −NH_2_ group. In the complexes, the bands shift to higher frequencies and appear at 3488 and 3373 cm^–1^ for **1** and 3488 and 3374 cm^–1^ for 2, compared with the ligand spectrum. Furthermore, the bands originating from the −NH– group at 3225 (**L**), 3229 (**1**), and 3230 (**2**) cm^–1^ suggest that the sulfonamide group does not participate in metal ion coordination. In the 1592–1644 cm^–1^ range, characteristic stretching bands corresponding to the C=C_ring_ were observed in all compounds. However, the C=N stretching bands in the ligand occur at 1504 and 1419 cm^–1^. In contrast, in the spectra of the complexes, these bands shift significantly, indicating the involvement of nitrogen atoms in coordination to the metal ion. By comparison, we see that the shifts in the bands from the −SO_2_– group differ much less from one another, so we can definitively conclude that the oxygen atom from the −SO_2_– group is not involved in coordination; rather, this suggests that the oxygen atom participates in hydrogen bonding. Additionally, in the case of complexes, there are two intense bands originating from the PF_6_^–^ anion: (**1**) 832 cm^–1^ and (**2**) 834 cm^–1^. The FT-IR studies are consistent with SC-XRD results.

### Thermogravimetry

The TG analysis showed that **L** is stable up to 303 °C (Figure S24). After that, decomposition occurs in several stages. The DTG curve shows a peak with a maximum in the 303–335 °C temperature range, which is difficult to identify. This is followed by continuous decomposition starting at approximately 360 °C. At the end of the measurement, approximately 35% of the compound’s mass remained, indicating further decomposition at higher temperatures. For both complexes, an initial mass loss was observed at 49 and 81 °C, likely due to the evaporation of residual acetone (bp = 56 °C) and ethanol (bp = 78 °C) used during synthesis. For the complexes, both thermograms are very similar, reflecting their identical structures apart from the central metal. We can distinguish several minor mass losses of a few percent, the origin of which is unclear and difficult to identify, however, mass losses were observed in the temperature range of 313–325 °C, indicating the detachment of the PF_6_^–^ ion for **1** (20.2%/18.85% weight loss) and for **2** (17.41%/16.89% weight loss). This also confirms the successful completion of the complexation process. Next, a slight decrease was observed in the range of 430–437 °C, which is likely due to the detachment of −NH_2_ for **1** (1.87%/2.08% weight loss) and for **2** (1.68%/1.87% weight loss). In the final stage, right up until the end of the measurement, there is a significant, continuous decline, making it difficult to determine the source of the mass loss. For both complexes, at the end of the measurement at 1000 °C, the sample masses do not drop to zero. This is certainly due to the transition metals used, whose boiling points are 3695 °C for Rh and 4428 °C for Ir.

### Stability Study

The stability of the ligand **L** and coordination compounds **1** and **2** in solution was assessed by UV–vis spectroscopy in DMSO, DMSO/water (Figure S25), and mixtures of acetonitrile/water (Figure S26). Spectra were recorded after 0, 24, 48, and 72 h of incubation at room temperature for the DMSO solutions (Figure S25) and after 0 and 24 h of incubation for the acetonitrile/water mixtures (Figure S26). No spectroscopic changes were observed over time and across different solvent gradients, confirming the stability of the compounds tested under the experimental conditions used.

### Determination of the *n*-Octanol/water Partition Coefficient (log *P*)

The partition coefficient, expressed as log *P*, allowed for the assessment of the lipophilicity of the tested compounds, i.e., their affinity for the organic phase relative to the aqueous phase. The calibration curves prepared in advance were used to determine the relationship between absorbance and concentration (μM). The initial concentrations of the compounds under investigation were then calculated based on the absorbance values (Figures S27 and S28). For **L**, the log *P* value was 0.78 ± 0.07, indicating its lipophilic nature. This means that the ligand exhibits a greater affinity for the hydrophobic environment than for the aqueous phase. After the complexation process, a negative shift in the log *P* value was observed −0.65 ± 0.05 for **1** and −0.91 ± 0.02 for **2**, respectively. This indicates increased hydrophilicity of complexes 1 and 2 and a greater affinity for the aqueous environment. This change shows that coordination strongly alters the ligand’s solution properties, however, log *P* alone does not determine membrane permeability or cellular uptake, which this study did not directly measure. A useful comparison is provided by the isostructural Cp* Rh­(III)/Ir­(III) 2,2′-biimidazole pair reported by Gilewska et al., for which log *P* values of −0.54 ± 0.02 (Rh) and +1.55 ± 0.04 (Ir) were obtained.^29^ In contrast, both **1** and **2** in the present phenanthroline–sulfonamide series are more hydrophilic. Thus, lipophilicity in Rh­(III)/Ir­(III) half-sandwich systems is highly sensitive to the ligand environment and cannot be predicted solely from the identity of the metal center. Therefore, complexation affects the compound’s physicochemical properties, increasing its water solubility and decreasing its lipophilicity. The complexes are more hydrophilic than the lipophilic sulfonamide ligand. Consequently, their penetration of the cell membrane may be somewhat limited, resulting in reduced cytotoxicity. Cisplatin is a well-known anticancer drug. Its logP values reported in the literature range from about −1.7 to −2.3.^58–60^ These values clearly indicate its hydrophilic nature, which is also in agreement with log *P* for complexes **1** and **2** qualitatively.

### CA Inhibition Studies

Carbonic anhydrase isolated from bovine erythrocytes, consisting predominantly of the well-characterized CA II isoform, was used as a model enzyme for the initial evaluation of inhibitory activity. Although CA II is not cancer-associated, it is routinely employed as a reference isoform before studies on therapeutically relevant CA IX and CA XII. Before the inhibition studies, the Michaelis constant (*K*_m_) for the hydrolysis of 4-nitrophenyl acetate was determined under our experimental conditions. Although *K*_m_ values for this substrate have been reported previously, an independent determination was performed because kinetic parameters depend on assay conditions, including enzyme source, buffer composition, pH, and temperature. The obtained *K*_m_ value was 5.01 mM (95% Cl: 3.52–7.30). This K_m_ value agrees well with previously reported values for the hydrolysis of 4-nitrophenyl acetate catalyzed by bovine erythrocyte carbonic anhydrase, which are typically in the low millimolar range.^61,62^ These findings indicate that the assay conditions used in this study are consistent with those described in the literature and provide reliable kinetic parameters for subsequent inhibition studies. The inhibitory activity of the synthesized compounds was initially assessed by determining their IC_50_ values. Compound **1** was tested in the range of 120–780 μM and compound **2** at 180–840 μM (Figure S29). The obtained IC_50_ values were 489.3 μM (95% CI: 469.5–510.1 μM) and 649.7 μM (95% CI: 619.0–685.7 μM), respectively (Table S4). In contrast, the free ligand **L** displayed only weak inhibition within the tested concentration range (Figure S30). Together with its limited aqueous solubility, this prevented reliable determination of its IC_50_ value. Overall, the synthesized complexes are relatively weak CA II inhibitors; however, the enhancement of activity upon metal coordination justifies further evaluation against the cancer-associated CA IX and CA XII isoforms. The higher inhibitory activity of the metal complexes compared with the free ligand highlights the beneficial effect of metal coordination. Although both complexes were markedly less potent than acetazolamide, this comparison should be interpreted with caution. Acetazolamide is a classical primary sulfonamide, representing the most extensively studied class of carbonic anhydrase inhibitors that directly coordinate the catalytic Zn^2+^ ion and typically exhibit high affinity toward the enzyme.^63^ In contrast, the synthesized compounds contain a secondary sulfonamide, a zinc-binding group generally associated with substantially lower inhibitory potency. Therefore, the improved activity of the metal complexes relative to the free ligand suggests that metal coordination partially compensates for the intrinsically weaker inhibitory properties of the secondary sulfonamide scaffold.

### CT-DNA Binding Studies

Because DNA is a key target of anticancer therapy, a study was conducted to investigate the interactions of **L** and its two coordination compounds, **1** and **2**, with CT-DNA using UV–vis spectroscopy (Figure S31). Binding ability was assessed based on changes in absorbance values recorded at various CT-DNA concentrations and constant concentrations of the tested compounds. However, the absorption maximum of CT-DNA coincides with the absorption maxima of the tested compounds, which significantly complicates the interpretation of the obtained spectra. Consequently, it is difficult to determine whether the observed increase in absorbance results solely from increasing CT-DNA concentration or from interactions between DNA and the tested compounds. No additional effects were observed in the 300–500 nm range, which may suggest no interaction between the compounds and DNA.

### Study of HSA Interactions

Serum albumin is one of the most abundant proteins in blood. It plays many important roles in the body, such as maintaining oncotic pressure, but above all, it transports drugs to their target sites. That is why it is important to test potential drug candidates to determine whether they can bind to human serum albumin (HSA).^64^ The interactions of compounds **L**, **1**, and **2** with HSA were studied using the UV–vis method. As shown in Figure 3, absorbance increases with concentration. The *K*_b_ values for complexes **1** and **2** were calculated as 4.63 × 10^2^ and 9.10 × 10^2^, respectively. Unfortunately, it was not possible to determine the *K*_b_ value for **L** using this technique. *K*_b_ values on the order of 10^2^ suggest that the tested compounds have low affinity for HSA. For example, cisplatin, a well-known anticancer drug, has *K*_b_ = 8.52 × 10^2^.^65^ Values of the order of 10^3^ are considered moderate,^66^ while values of 10^4^ and above indicate high affinity.^67^ We are primarily interested in albumin’s transport function. High affinity for HSA may lead to lower concentrations of free drug, whereas weaker binding may increase free drug and promote faster metabolism or elimination. Therefore, it is important to maintain a balance in albumin binding. After complexation, which increased the hydrophilicity of the tested compounds, binding to HSA may have strengthened through the formation of, for example, hydrogen bonds.^68^

**Figure 3:**
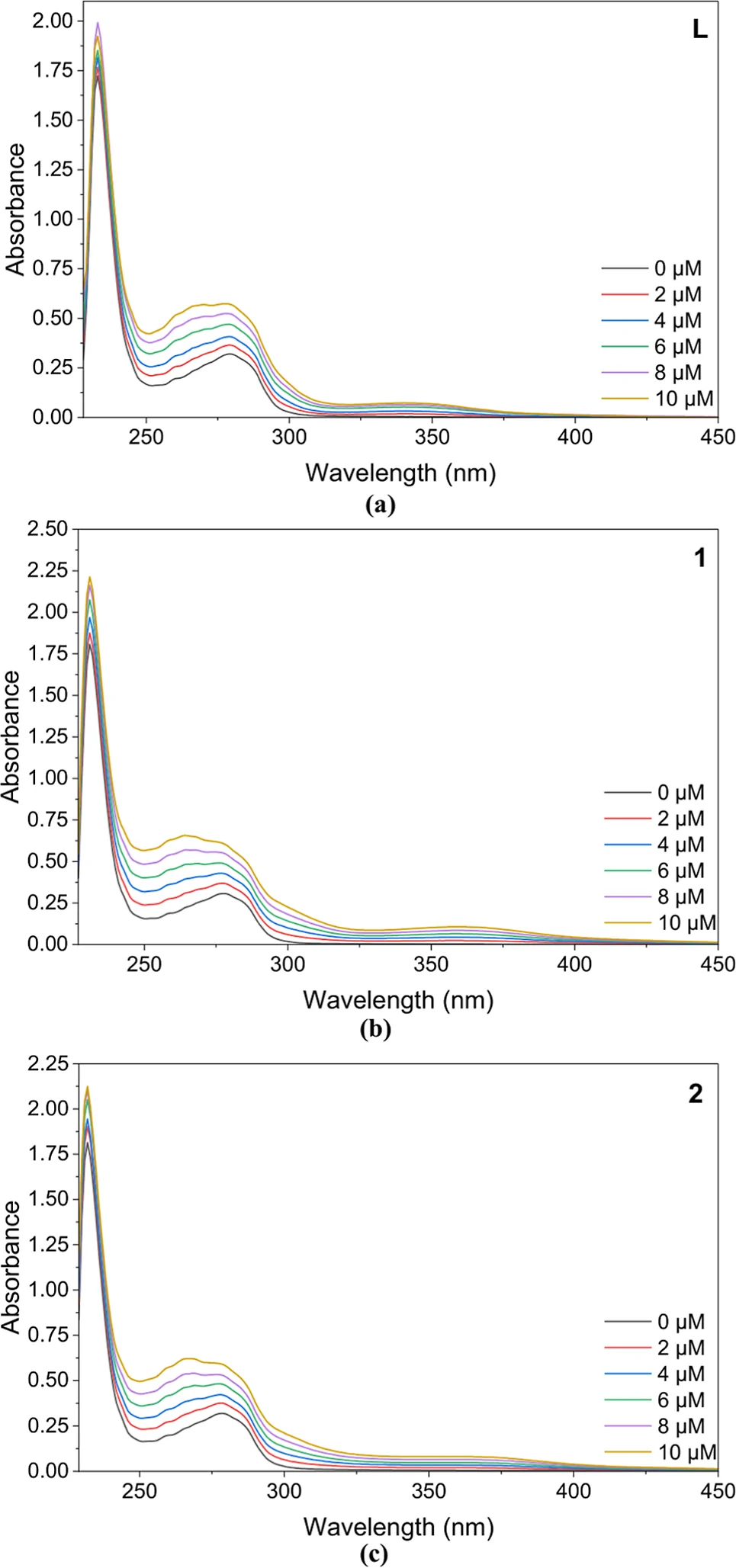
Changes in the UV–vis spectra of HSA induced by increasing concentrations of compound **L** (a), complex **1** (b), and complex **2** (c).

### Comparative Cytotoxic Profiles Across Cancer and Non-malignant Cell Lines

To quantitatively assess the antiproliferative potency of the tested compounds, we performed a 72 h MTT (3-(4,5-dimethylthiazol-2-yl)-2,5-diphenyltetrazolium bromide) assay across a panel of cancer cell lines representing different tissue origins—breast adenocarcinoma (MCF-7), nonsmall cell lung cancer (A-549), colorectal carcinoma (HCT-116), and acute lymphoblastic leukemia (NALM-6)—as well as nontransformed embryonic kidney cells (HEK-293) used as a model for healthy proliferating cells. Results are expressed as IC_50_ values, denoting the concentration required to inhibit cell viability by 50% (Table 3). **L** exhibited potent cytotoxic activity, with IC_50_ values ranging from 0.55 μM in NALM-6 to 1.87 μM in MCF-7, while retaining moderate overall selectivity toward cancer cells compared with nontransformed HEK-293 cells (SI = 3.06). Complexation with rhodium­(III) resulted in **1**, which showed reduced cytotoxicity across all cancer cell lines (IC_50_ values ∼ 32–37 μM) but retained some selectivity toward NALM-6 (IC_50_ = 4.41 μM) and favorable overall selectivity (SI > 3.78). In contrast, **2** showed intermediate potency (IC_50_ = 1.85–6.99 μM) but limited overall selectivity (SI = 1.52), reflecting broader cytotoxicity, including in nonmalignant cells (HEK-293 IC_50_ = 7.25 μM). Cisplatin (CCPD), included as a reference drug, showed strong cytotoxicity in A-549 and NALM-6 cells (IC_50_ = 2.97 μM and 0.83 μM, respectively), but limited overall selectivity (SI = 1.72), reflecting broader cytotoxicity, including in nonmalignant cells (IC_50_ = 30.59 μM). Based on its enhanced selectivity toward leukemia cells, **1** was selected for further mechanistic studies. **L**, owing to its superior cytotoxic potency, was also included in downstream assays as a comparative ligand control.

**Table 3:** In vitro Cytotoxic Activity (IC_50_ ± SD, μM) of the Tested Compounds against a Panel of Human Cancer Cell Lines: Breast Adenocarcinoma (MCF-7), Non-small Cell Lung Carcinoma (A-549), Colorectal Carcinoma (HCT-116), Acute Lymphoblastic Leukemia (NALM-6), and Embryonic Kidney Cells (HEK-293).^a^

compound	MCF-7	A-549	HCT-116	NALM-6	HEK-293	SI
	IC_50_ (μM)					
**L**	1.87 ± 0.25	1.55 ± 0.59	1.46 ± 0.07	0.55 ± 0.1	4.16 ± 0.19	3.06
**1**	37.02 ± 4.58	32.56 ± 1.08	31.72 ± 4.87	4.41 ± 0.4	>100	>3.78
**2**	5.66 ± 0.54	4.59 ± 0.69	6.99 ± 0.89	1.85 ± 0.0	7.25 ± 0.30	1.52
**CCPD**	63.48 ± 5.03	2.97 ± 0.09	4.16 ± 0.58	0.83 ± 0.1	30.59 ± 2.07	1.71

a IC_50_ values represent concentrations required to inhibit 50% of cell viability after 72 h of treatment. Overall selectivity index (SI) was calculated as IC_50_ (HEK-293)/mean IC_50_ (cancer cell lines).

### Cell Cycle Arrest Driven by S-Phase Accumulation

To assess the impact of the tested compounds on cell cycle progression, NALM-6 cells were treated with **L**, its rhodium­(III) complex **1**, or the reference drug CCPD at IC_90_ concentrations for 24 and 48 h. DNA content analysis was performed by flow cytometry following propidium iodide staining (Figure 4a). At 24 h, both **L** and **1** induced a cell cycle redistribution, increasing the S-phase population to 38.0% and 35.7%, respectively, relative to 31.5% in vehicle-treated cells (Figure 4b). This shift was accompanied by a reduction in the G_1_ population to 27.5% (**L**) and 20.6% (**1**). G_2_/M phase proportions remained largely unaffected. These changes suggest early interference with S-phase progression. At 48 h, **1** induced a more pronounced phenotype, with S-phase accumulation reaching 42.0%, while G_1_ and G_2_/M fractions declined to 8.0% and 10.4%, respectively (Figure 4b). **L** produced a comparable but less intense profile (S: 40.0%; G_1_: 16.1%; G_2_/M: 11.9%). Quantitative comparison confirmed that **1** induced significantly stronger S-phase retention than **L** (*p* < 0.0001), suggesting enhanced engagement with replication-associated checkpoints or disruption of DNA synthesis. In contrast, CCPD-treated cells exhibited an increase in the G_1_ population at 48 h (41.5%), accompanied by a reduction in S phase (23.5%) (Figure 4b). While CCPD is commonly associated with S-phase stalling or G_2_/M arrest, the observed G_1_ enrichment in NALM-6 cells likely reflects p53-dependent checkpoint activation, consistent with the wild-type TP53 status of this cell line.^69,70^

**Figure 4:**
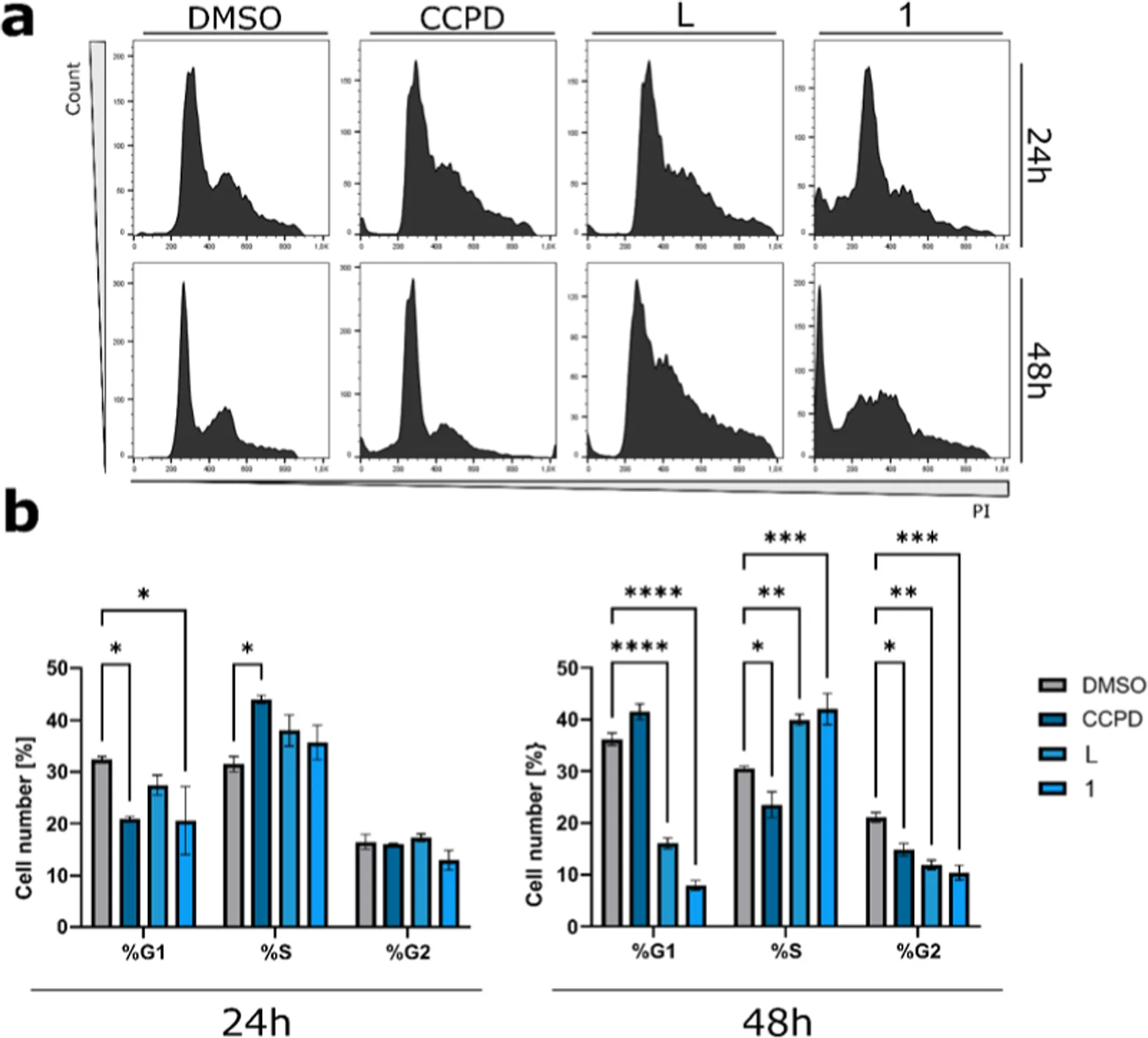
Analysis of the cell cycle profile in NALM-6 cells after 24 and 48 h of exposure to selected compounds at their respective IC_90_ concentrations (a), accompanied by a quantitative summary of the results (b). Values represent mean ± SD (*n* = 3). Statistical significance: *p < 0.01, **p < 0.001, ***p < 0.0001, ****p < 0.00001 compared to the vehicle control (two-way ANOVA followed by Dunnett’s post hoc test).

### Apoptosis Induction by **L** and **1**

To determine whether the tested compounds promote apoptotic cell death, NALM-6 cells were treated with **L**, its rhodium­(III) complex **1**, and the reference drug CCPD at IC_90_ concentrations for 24 and 48 h. Apoptosis was assessed by dual staining with Annexin V-FITC and 7-AAD, allowing discrimination between viable, early apoptotic, late apoptotic, and necrotic populations (Figure 5a). At 24 h, **L** led to a modest apoptotic response, with 17.8% of cells in early apoptosis and 6.95% in late apoptosis (Figure 5b). In contrast, **1** exhibited a more potent effect, with early and late apoptotic fractions reaching 25.0% and 12.3%, respectively, alongside a noticeable reduction in viability. By 48 h, **1** further increased apoptosis, with over 40% of cells undergoing late apoptotic death, compared with ∼35% in the **L** group (Figure 5b). This difference became more pronounced after 48 h. Treatment with **1** led to a cumulative apoptotic index exceeding 68%, driven by increases in both early (28.1%) and late (40.3%) apoptosis. In contrast, **L** reached a combined value of ∼58%. While both compounds induced a time-dependent apoptotic response, **1** consistently exhibited greater potency. CCPD triggered a rapid and distinct apoptotic profile. At 24 h, it rapidly triggered extensive apoptosis, characterized by a high fraction of late apoptotic cells (Figure 5b). At 48 h, necrotic cells increased, suggesting progression to secondary necrosis or loss of membrane integrity. Although CCPD effectively induced cell death, its mode of action appeared to involve more abrupt cytotoxicity, in contrast to the progressive apoptotic engagement seen with **1**.

**Figure 5:**
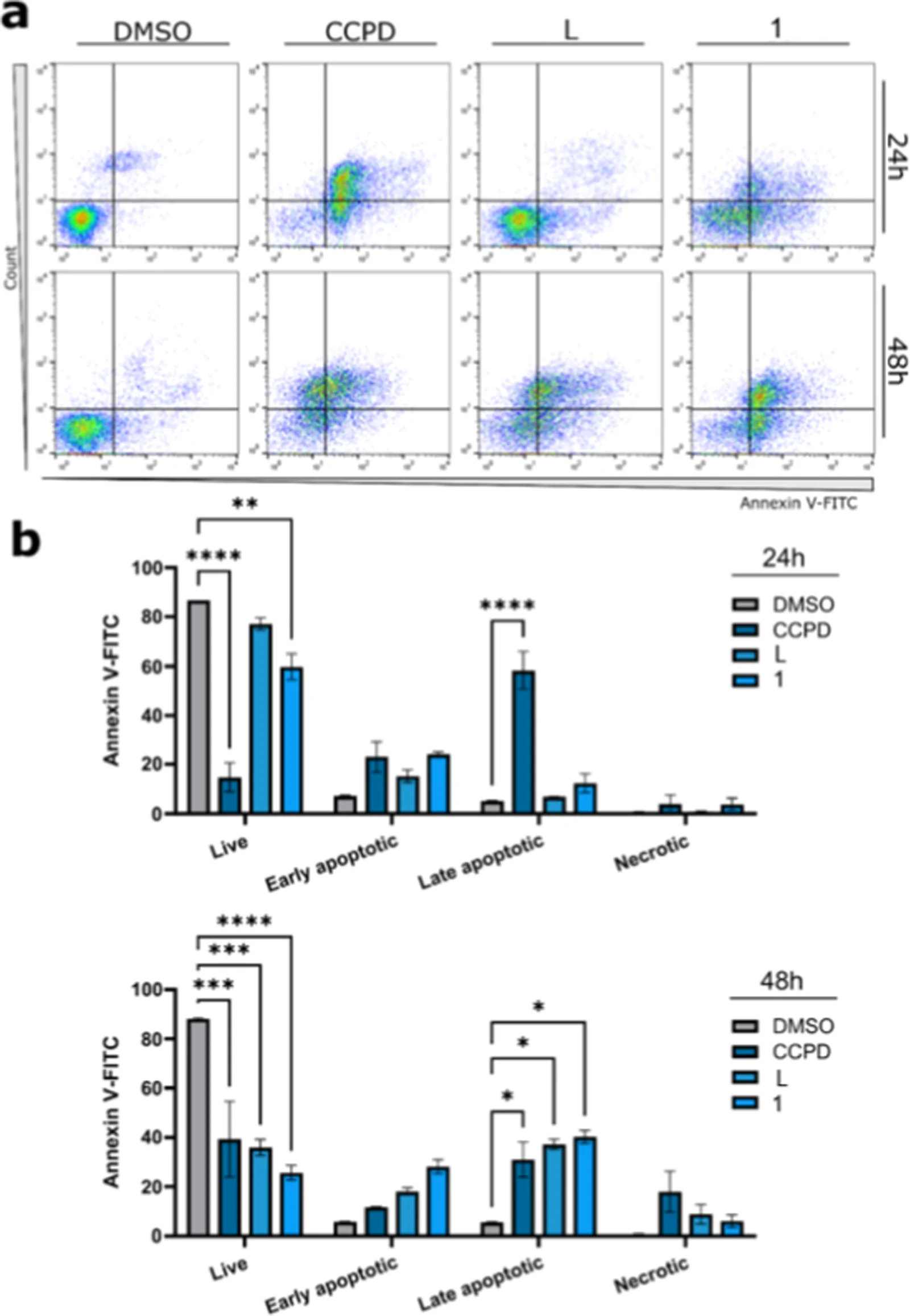
Evaluation of apoptosis in NALM-6 cells treated with selected compounds at IC_90_ concentrations for 24 and 48 h (a), with representative flow cytometry dot plots based on Annexin V-FITC and 7-AAD staining, and corresponding quantitative analysis (b). Values represent mean ± SEM (*n* = 3). Statistical significance: *p < 0.01, **p < 0.001, ***p < 0.0001, ****p < 0.00001 compared to the vehicle control (two-way ANOVA followed by Dunnett’s post hoc test).

To determine whether the observed apoptotic phenotype reflects activation of the canonical intrinsic death pathway, caspase-3/7 activity was measured after 24 and 48 h of treatment with **L**, **1**, and CCPD. Fluorescence-based detection of DEVD-cleaving enzymatic activity revealed differential engagement of the caspase cascade across compounds and time points (Figure 6a). At 24 h, **1** treatment rapidly and robustly activated caspase-3/7, with signal intensities comparable to those induced by CCPD (Figure 6b). In contrast, **L** elicited only a modest increase in caspase activity, consistent with its lower apoptotic index at this time point. This early trend supports more rapid apoptotic engagement following metal complexation. By 48 h, all tested compounds induced marked caspase activation (Figure 6b). **1** remained the most active, with signal intensities exceeding those of both CCPD and **L**. Each treatment group showed a significant increase relative to the control (***p < 0.0001 for 1 and L; ****p < 0.00001 for CCPD), confirming caspase-dependent mechanisms. These findings suggest that **1** engages the executioner caspase machinery early, in parallel with its pro-apoptotic activity, and sustains this response over time.

**Figure 6:**
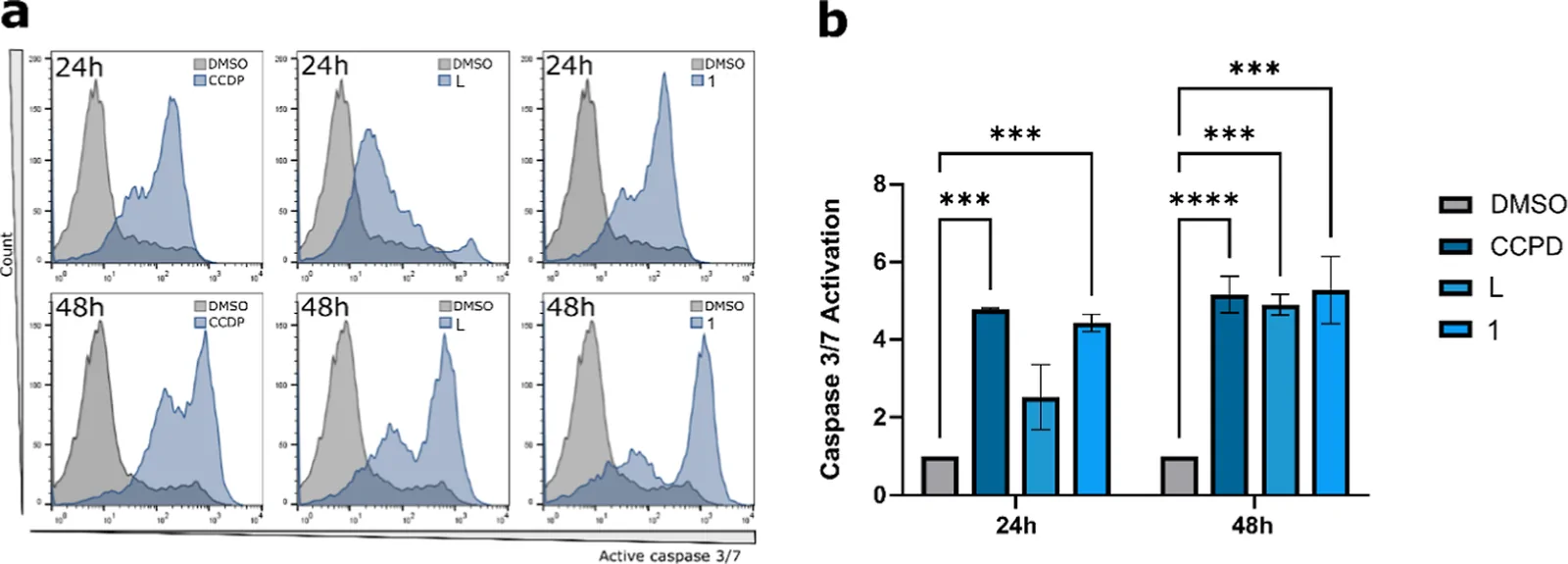
Caspase-3/7 activation in NALM-6 cells following treatment with selected compounds at IC_90_ concentrations for 24 and 48 h (a), visualized by representative histograms of caspase-3/7 fluorescence intensity, accompanied by quantitative analysis of caspase-3/7-positive cell populations (b). Values represent mean ± SEM (*n* = 3). Statistical significance: *p < 0.01, **p < 0.001, ***p < 0.0001, ****p < 0.00001 compared to the vehicle control (two-way ANOVA followed by Dunnett’s post hoc test).

### Evaluation of Mitochondrial Membrane Potential Reveals Divergent Effects of **L** and **1**

To test whether the compounds affect mitochondrial integrity, a hallmark of intrinsic apoptosis, mitochondrial membrane potential (ΔΨ*m*) was evaluated using JC-1 dye staining followed by flow cytometric analysis (Figure 7a).^71^ In polarized mitochondria, JC-1 forms fluorescent aggregates, whereas mitochondrial depolarization promotes its accumulation in the monomeric form. Therefore, an increase in JC-1 monomer fluorescence reflects a loss of mitochondrial membrane potential.^72^ Treatment with **L** resulted in a rapid and pronounced dissipation of ΔΨ*m* (Figure 7). As early as 6 h post-treatment, JC-1 monomer fluorescence increased to a mean of 1.26-fold, rising further to 3.89-fold at 24 h, indicating progressive mitochondrial depolarization (Figure 7b). By contrast, **1** induced only modest mitochondrial depolarization, as indicated by a limited increase in JC-1 monomer fluorescence at both time points (1.37-fold at 6 h; 1.80-fold at 24 h), suggesting that despite its strong pro-apoptotic activity, loss of ΔΨ*m* is not an early or dominant event following treatment with **1**. This apparent disconnect between **1**’s strong apoptotic phenotype and its limited disruption of mitochondrial membrane potential suggests that mitochondrial dysfunction may play a less prominent role in the apoptotic response induced by **1.** Alternatively, **1** may engage a redox-driven apoptotic pathway characterized by limited early mitochondrial depolarization.^73^ These findings indicate a different contribution of mitochondrial dysfunction compared with **L** and suggest that metal coordination modulates the sequence and nature of apoptotic signaling events. FCCP, used as a positive control, triggered a robust and sustained increase in the proportion of cells with depolarized mitochondria, reaching 1.86-fold and 4.22-fold at 6 and 24 h, respectively, validating the assay’s responsiveness (Figure 7b). CCPD, used as a reference compound, caused a moderate but time-dependent loss of mitochondrial potential, with JC-1 monomer fluorescence increasing to approximately 1.3-fold at 6 h and 3.65-fold at 24 h, consistent with its known mitochondrial toxicity and delayed-onset apoptosis.^74^

**Figure 7:**
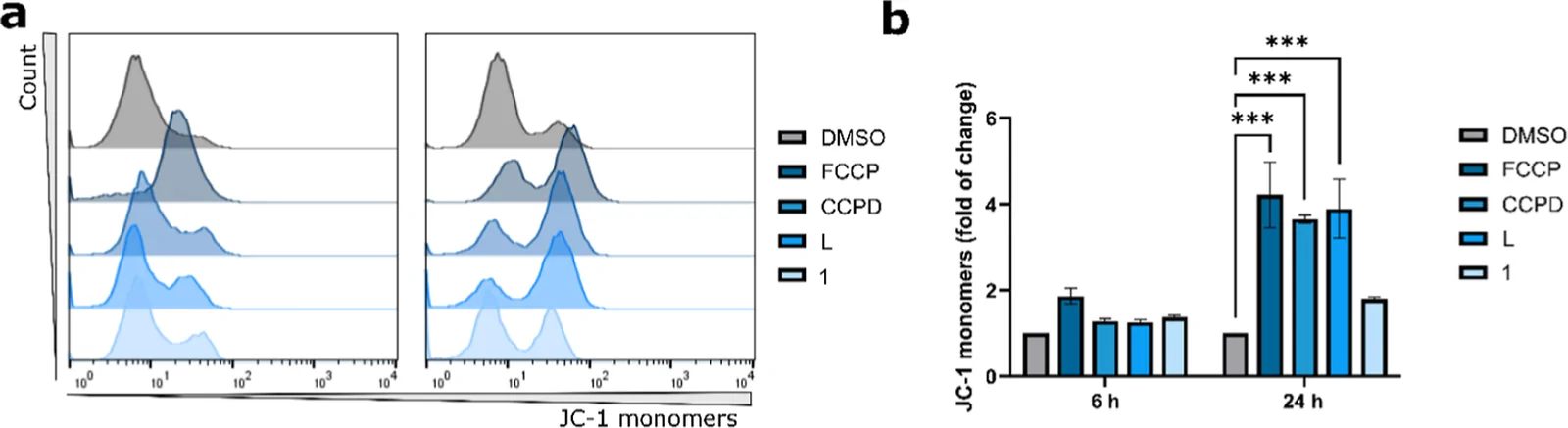
Assessment of mitochondrial membrane potential (ΔΨ*m*) in NALM-6 cells treated with selected compounds at IC_90_ concentrations for 6 and 24 h (**a**), illustrated by representative flow cytometry histograms of JC-1 fluorescence intensity, with corresponding quantitative analysis of ΔΨ*m* changes (**b**). Results are shown as mean ± SEM from three independent experiments (*n* = 3). Statistical significance: *p < 0.01, **p < 0.001, ***p < 0.0001, ****p < 0.00001 compared to the vehicle control (two-way ANOVA followed by Dunnett’s post hoc test).

### Induction of DNA Double-Strand Break Signaling via γH2AX Phosphorylation

To elucidate the relationship between DNA damage and the observed pro-apoptotic effects, we next quantified the phosphorylation of histone H2AX at Ser139 (γH2AX), a sensitive marker of DNA double-strand breaks (DSBs) and early chromatin-level stress (Figure 8a). H2AX phosphorylation is a rapid response to genotoxic insults and often precedes both cell cycle arrest and commitment to apoptosis. After 24 h of treatment, both CCPD and **1** induced strong γH2AX signals, with ∼34–35% and ∼29–32% of γH2AX-positive cells, respectively, indicating substantial DNA damage burden (Figure 8b). This coincided temporally with early caspase-3/7 activation (Figure 6) and the onset of apoptosis (Figure 5), suggesting that DNA damage may contribute to the subsequent apoptotic response in both cases. In contrast, **L** elicited only background-level γH2AX signals (<0.8%), consistent with its weaker early pro-apoptotic activity (Figure 8b). By 48 h, CCPD-treated cells retained a high proportion of γH2AX-positive nuclei, consistent with persistent or unresolved DNA lesions (Figure 8b). This likely reflects sustained replication stress and stalled repair, hallmarks of CCPD’s mechanism of action in rapidly dividing tumor cells.^75^ In contrast, **1**-treated cells exhibited a pronounced reduction in γH2AX signal (to ∼9.5–12.6%), despite clear evidence of ongoing apoptosis and caspase activity (Figure 8b). This decline likely reflects the chromatin condensation and nuclear fragmentation associated with execution-phase apoptosis, during which γH2AX may be degraded by apoptotic nucleases or become inaccessible to detection due to structural disassembly.^76,77^ Notably, **L** remained weakly active throughout, with γH2AX-positive fractions not exceeding 1.2% at either time point.

**Figure 8:**
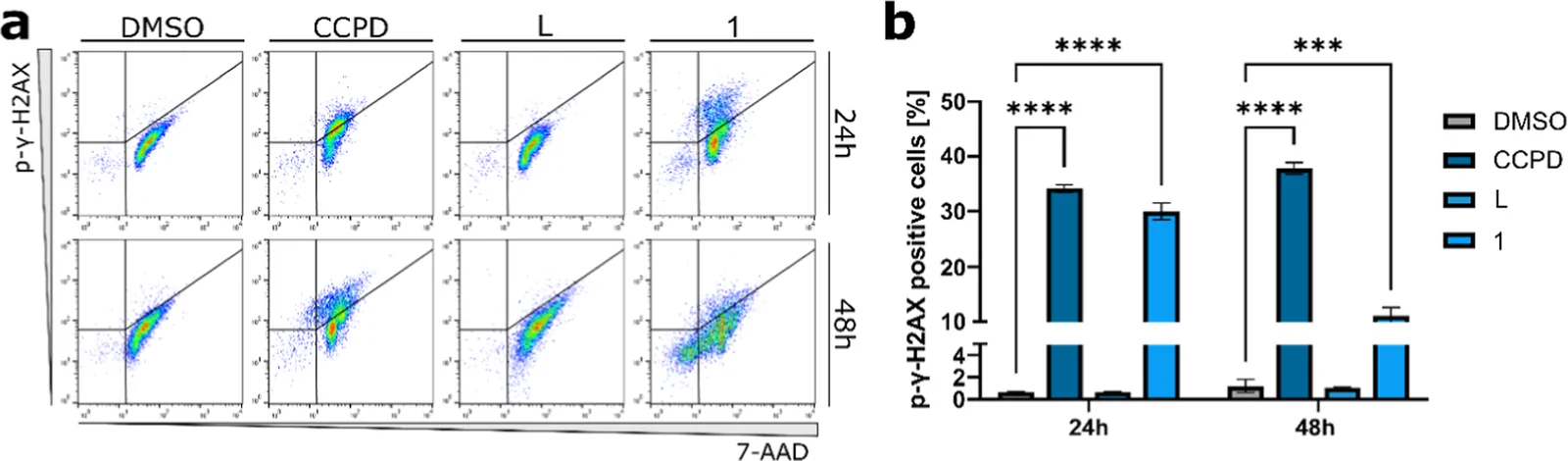
Phosphorylated γH2AX expression in NALM-6 cells treated with selected compounds at IC_90_ concentrations for 24 and 48 h (a), shown by representative flow cytometry dot plots, along with quantitative analysis of p-γH2AX-positive cell populations (b). Data are presented as mean ± SEM (*n* = 3). Statistical significance: ***p < 0.0001, ****p < 0.00001 compared to the vehicle control (two-way ANOVA followed by Dunnett’s post hoc test).

### Compound-Specific Kinetics of ROS Generation

To evaluate the involvement of oxidative stress in the cytotoxic mechanisms of the tested compounds, intracellular reactive oxygen species (ROS) generation was assessed using the fluorogenic probe H_2_DCFDA at multiple time points (1, 3, 6, and 24 h) following treatment with **L**, **1** and the reference agent CCPD (Figure 9a,b). The redox-active environment is a known modulator of both caspase-dependent cell death and DNA damage, and may therefore contribute to the apoptotic phenotype observed in subsequent assays. At early time points (1–3 h), both **L** and **1** induced a mild increase in the proportion of ROS-positive cells (∼7–9% at 1 h and ∼13–15% at 3 h), indicating a moderate and comparable redox perturbation during initial exposure. However, by 6 h, **1** triggered a markedly stronger oxidative response (∼59% ROS-positive cells), whereas **L**-treated cells reached ∼33.5%, suggesting compound-specific differences in redox stress kinetics. Despite the earlier ROS peak, execution-phase apoptosis, as evidenced by caspase-3/7 activation, was first detected at 24 h, implying a temporal separation between redox imbalance and caspase engagement. This temporal pattern is consistent with oxidative stress preceding caspase activation and apoptosis, although the present data do not establish a causal relationship. At 24 h, ROS levels subsided but remained elevated relative to vehicle controls, with **1**- and **L**-treated cells maintaining ∼22% and ∼10% ROS-positive populations, respectively. These persistent ROS elevations likely contribute to cumulative intracellular stress and damage. The delayed induction of γH2AX positivity, observed primarily at 24 and 48 h, suggests that oxidative and apoptotic stress may culminate in secondary DNA damage rather than direct genotoxicity. CCPD induced a transient oxidative response, peaking at 3 h (∼23% ROS-positive cells), followed by a decrease to ∼ 14% at 6 h and further attenuation at 24 h. This pattern aligns with its known mechanism of mitochondrial dysfunction and ROS-mediated DNA damage as a downstream consequence of cellular stress.^78^ As a validation control, treatment with hydrogen peroxide (H_2_O_2_) rapidly induced robust ROS accumulation (>75% ROS-positive cells within 1 h), confirming the dynamic range and responsiveness of the assay.

**Figure 9:**
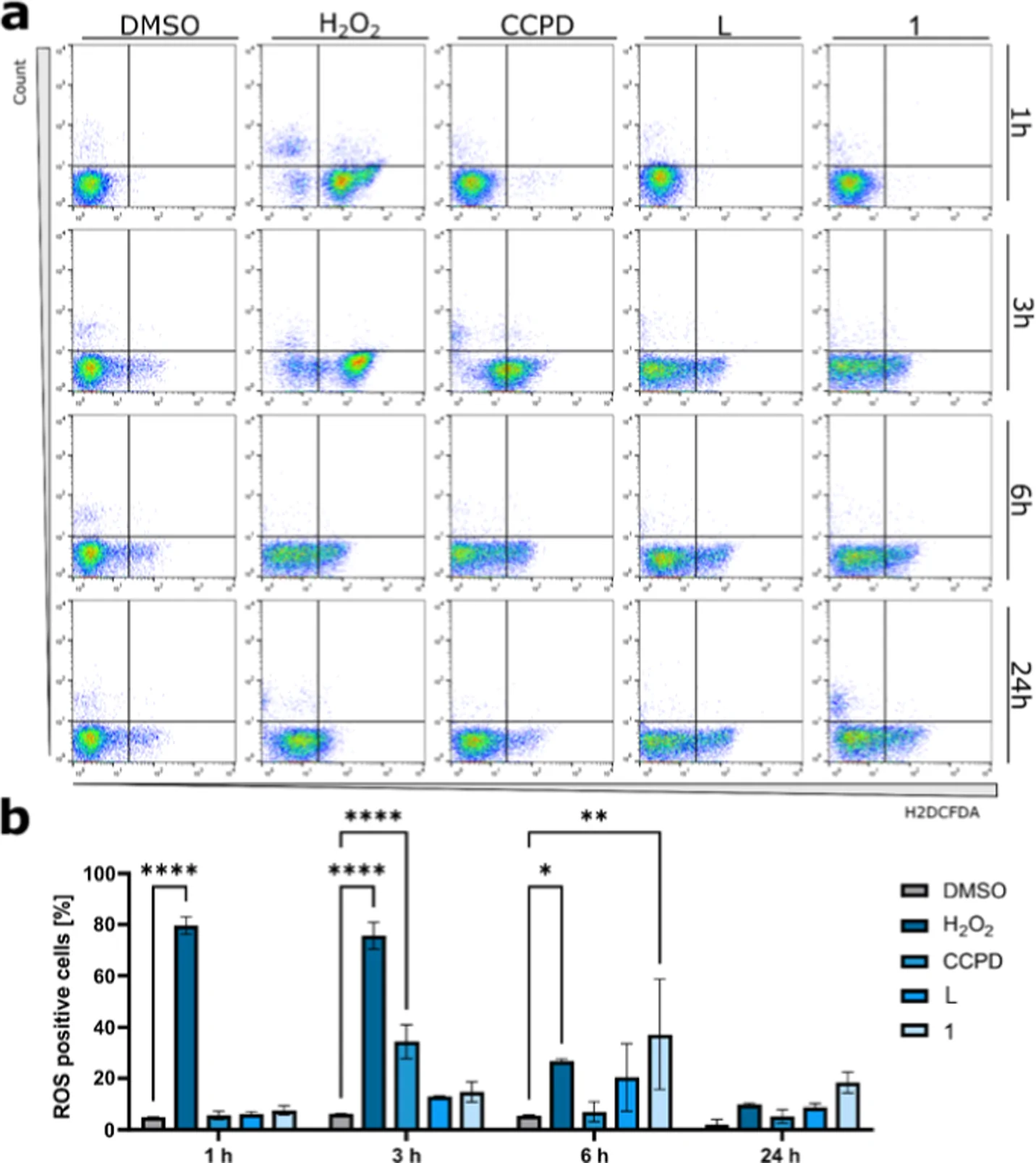
Intracellular ROS generation in NALM-6 cells following treatment with selected compounds at IC_90_ concentrations (a), illustrated by representative flow cytometry dot plots of H_2_DCFDA fluorescence, and corresponding quantitative analysis of ROS-positive populations (b). Data are expressed as mean ± SEM from three independent experiments (*n* = 3). Statistical significance: *p < 0.01, **p < 0.001, ***p < 0.0001 versus vehicle control (two-way ANOVA followed by Dunnett’s post hoc test).

### Temporal Comparison of **L**- and **1**-Induced Cellular Responses

The time-resolved assays reveal a different weighting of early stress responses for **L** and **1**. At 6 h, **1** displays its strongest measured ROS response while mitochondrial depolarization remains comparatively modest. By 24 h, the ROS-positive fraction has declined, whereas caspase-3/7 activation, phosphatidylserine externalization, and γH2AX signaling have become prominent. **L** follows a different pattern: its ROS response is weaker, while mitochondrial depolarization becomes much more pronounced by 24 h, and its early caspase and γH2AX responses remain lower than those of **1**. By 48 h, both compounds produce substantial apoptosis. These observations therefore support mechanistic divergence between the free ligand and the Rh­(III) complex, but they define temporal association rather than a complete causal pathway. In particular, the data are consistent with a redox-associated response to **1** in which major ΔΨ*m* loss is not an early dominant event. In contrast, mitochondrial depolarization is more prominent during the response to **L**.

### Metal-dependent structure–property–activity Relationships

The matched **L**/**1**/**2** series provides a compact structure–property–activity comparison in which the ligand framework is held constant while coordination and metal identity are varied. Coordination converts the comparatively lipophilic free ligand **L** into hydrophilic cationic complexes, while Rh­(III) and Ir­(III) derivatives retain closely analogous piano-stool structures. Despite this structural similarity, cellular profiles diverge substantially. Rh­(III) complex **1** loses much of the broad potency of **L** in adherent tumor models but retains activity against NALM-6 cells together with markedly reduced HEK-293 toxicity. Ir­(III) complex **2** remains active at low-micromolar concentrations but shows substantially weaker overall discrimination between malignant and nonmalignant cells. The negative log *P* values of both complexes indicate that this selectivity difference cannot be explained by lipophilicity itself. The NALM-6 time-course further shows that Rh­(III) coordination changes not only potency but also the relative timing of ROS generation, mitochondrial depolarization, DNA-damage signaling, and caspase-dependent apoptosis. Collectively, these results identify metal identity as an active design variable within this phenanthroline–sulfonamide scaffold while also highlighting the need for direct measurements of cellular uptake, intracellular speciation, and pathway perturbation in future analogs.

## Conclusions

In this study, a new sulfonamide derivative containing a phenanthroline moiety (**L**) was synthesized and used to obtain two half-sandwich complexes of the general formula [(η^5^-Cp*)­MCl­(**L**)]­PF_6_, where M = Rh­(III) (**1**) or Ir­(III) (**2**). Structural and physicochemical characterization confirmed the formation and identity of the compounds and revealed that metal coordination markedly changes their solution properties. In particular, coordination shifts the relatively lipophilic ligand **L** toward more hydrophilic Rh­(III) and Ir­(III) complexes. However, the distinct biological profiles of **1** and **2** indicate that lipophilicity alone cannot account for the observed metal-dependent activity. The CT-DNA UV–vis experiments were limited by substantial spectral overlap and therefore did not allow reliable conclusions regarding a specific DNA-binding mode. HSA binding by the complexes was relatively weak, with binding constants on the order of 10^2^. In contrast, the carbonic anhydrase studies showed that both metal complexes were stronger inhibitors than the free ligand.

The biological results further demonstrate that metal coordination substantially reshapes the activity of the phenanthroline–sulfonamide scaffold. The free ligand **L** showed pronounced cytotoxic activity, including submicromolar IC_50_ values in selected tumor cell lines, while retaining favorable selectivity toward nonmalignant HEK-293 cells. Among the metal complexes, Rh­(III) complex **1** provided the most favorable balance between activity against NALM-6 acute lymphoblastic leukemia cells and low HEK-293 toxicity. In contrast, Ir­(III) complex **2**, although retaining micromolar anticancer activity, showed weaker overall discrimination between malignant and nonmalignant cells. Thus, the results indicate that preserving cytotoxic potency alone is not sufficient to optimize this scaffold and that metal-dependent effects on selectivity must also be considered.

Mechanistic studies in NALM-6 cells revealed clear differences between **L** and **1**. Complex **1** induced a pronounced oxidative burst, with the strongest ROS response observed at 6 h, preceding prominent caspase-3/7 activation, phosphatidylserine externalization, and γH2AX accumulation. At the same time, mitochondrial depolarization remained comparatively limited. The dissociation between modest ΔΨ*m* disruption and robust caspase activation suggests that extensive early mitochondrial dysfunction is unlikely to be the dominant event in the apoptotic response induced by **1**. However, mitochondrial participation cannot be excluded.^76,77^ The temporal sequence of ROS generation followed by apoptotic signaling and γH2AX accumulation is consistent with a redox-associated cellular stress response in which oxidative perturbation may contribute to subsequent replication stress and DNA damage.^79,80^ However, these data establish temporal association rather than causality, and direct ROS-rescue experiments will be required to determine whether ROS generation is mechanistically necessary for the response to **1**. Both **L** and **1** induced S-phase accumulation, with **1** producing a stronger and more sustained effect, whereas significant early γH2AX accumulation was observed predominantly for **1**. The subsequent decline in γH2AX is consistent with changes associated with execution-phase apoptosis, including chromatin condensation and DNA fragmentation.^74,75^ In contrast, **L** produced a weaker, more transient ROS response, along with more pronounced time-dependent mitochondrial depolarization, further demonstrating that Rh­(III) coordination shifts the balance between redox stress, mitochondrial perturbation, and downstream apoptotic signaling.

Taken together, these findings show that modification of the metal center within a closely related half-sandwich phenanthroline–sulfonamide framework can substantially alter physicochemical properties, cytotoxic selectivity, and the temporal pattern of cellular stress responses. In particular, Rh­(III) complex **1** emerges as a promising NALM-6-active, leukemia-oriented scaffold rather than a leukemia-targeted agent, since it has not been shown to target leukemia specifically. From a design perspective, future optimization should aim to preserve the favorable combination of NALM-6 activity and low HEK-293 toxicity observed for **1**, while systematically modifying the phenanthroline, arylsulfonamide, and Cp-derived components to tune hydrophobicity, cellular uptake, and intracellular reactivity. Direct measurements of cellular uptake and subcellular distribution, ROS-rescue experiments, and expanded testing across leukemia and nonmalignant hematopoietic cell panels will be essential to determine whether the response observed for **1** can be generalized and translated into a predictive structure–activity framework for the further development of organorhodium sulfonamide anticancer candidates.

## Supplementary Material



## Notes

ESI–MS spectrograms of **L**, **1**, and **2**; ^1^H, ^13^C, HSQC, COSY NMR spectra of **L**, **1**, and **2**; FT-IR spectra of **L**, **1**, and **2;** additional crystallographic information includes selected bond lengths and angles for **L** obtained from single-crystal X-ray diffraction; geometry of hydrogen bonds in its crystal structure for **L**; the crystal packing of complex **2**, viewed along the *a* axis; TG and DTG curves of **L**, **1**, and **2**; UV–vis spectra (stability studies in DMSO, DMSO/water, acetonitrile/water) of **L**, **1**, and **2**; calibration curves used for partition-coefficient measurements of **L**, **1**, and **2**; the corresponding linear relationships between absorbance and concentration of **L**, **1**, and **2**; carbonic anhydrase inhibition data of **L**, **1**, and **2**; UV–vis spectra of the interaction between **L**/**1**/**2** and CT-DNA (PDF)
