# Supplementary material for: Metal-dependent Structure, Solution Properties, and Redox-Mediated Anticancer Activity of Rhodium(III) and Iridium(III) Half-Sandwich Phenanthroline–Sulfonamide Complexes
Source: Inorg Chem. 2026 Oct 2;65(41):24456–75. doi: 10.1021/acs.inorgchem.6c04078 (PMC13685411; doi:10.1021/acs.inorgchem.6c04078)
Supplement: Supplementary file 1 [file ic6c04078_si_001.pdf]

Supporting Information

Metal-Dependent Structure, Solution Properties, and Redox-Mediated Anticancer Activity of Rhodium(III) and Iridium(III) Half-Sandwich Phenanthroline-Sulfonamide Complexes

Natalia Żukowska[1#], Mateusz Kowalik[1#], Natalia Maciejewska[2], Jan Alfuth[2], Dawid Dębowski[1], Katarzyna Kazimierczuk[2], Mariusz Makowski[1*]

[1] Faculty of Chemistry, University of Gdańsk, Wita Stwosza 63, 80-308 Gdańsk, Poland

[2] Faculty of Chemistry, Gdańsk University of Technology, Gabriela Narutowicza 11/12, 80-233 Gdańsk, Poland

[#]equally contributed

*corresponding author: mariusz.makowski@ug.edu.pl

Keywords: Ir(III) and Rh(III) half-sandwich complexes; phenanthroline-sulfonamide ligand; interactions with biomolecules; biological study; anticancer activity.

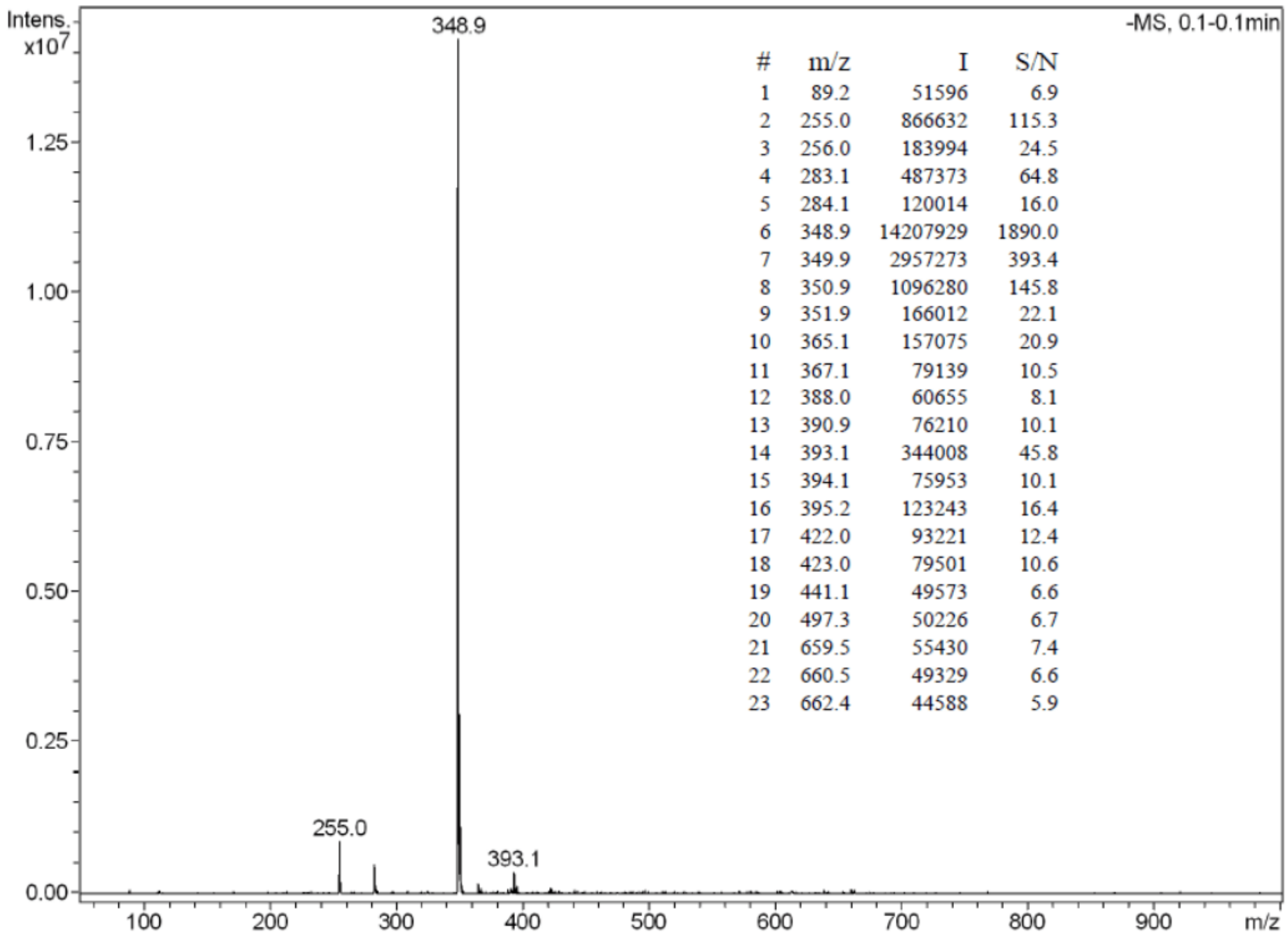

(a)

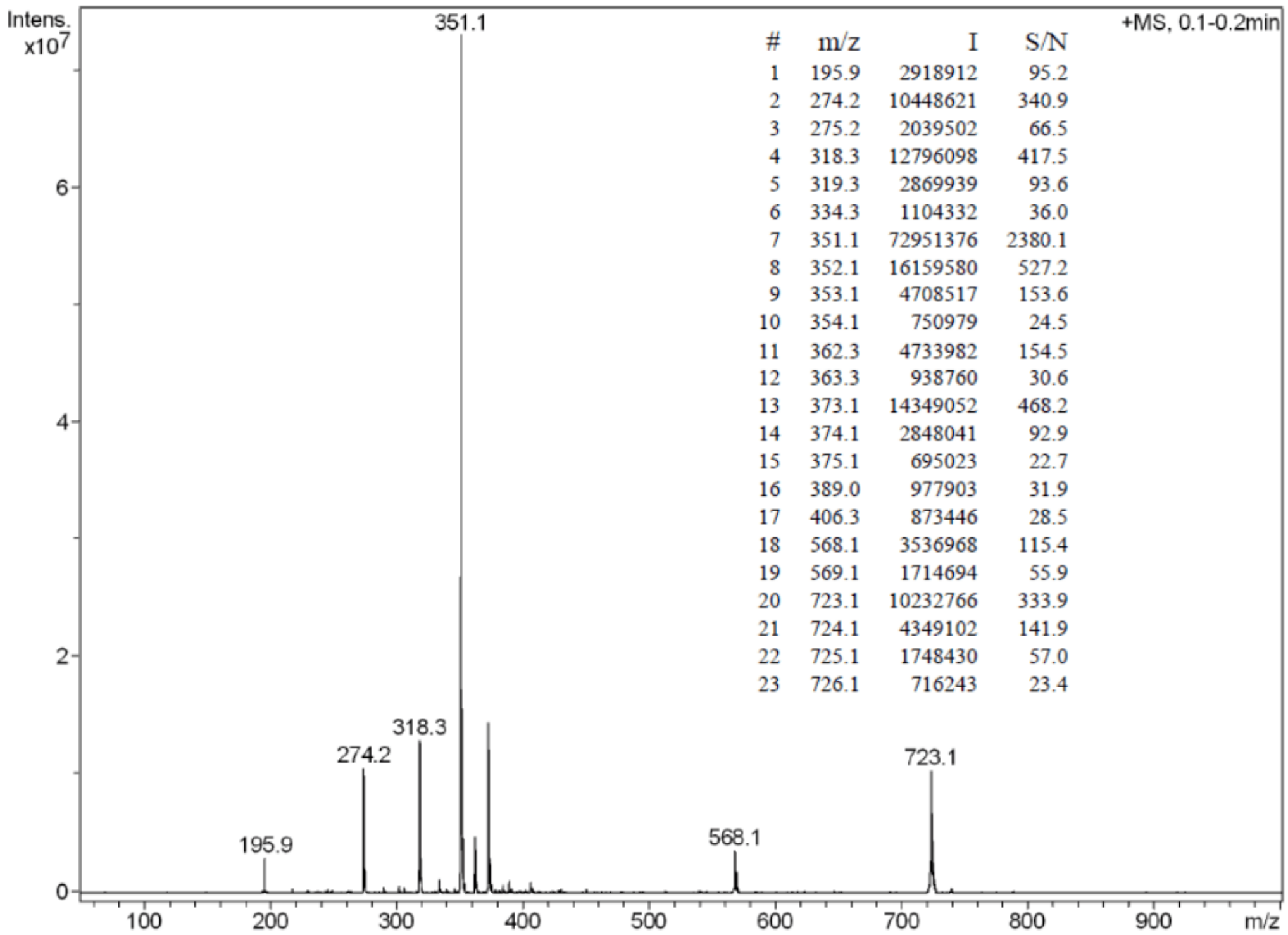

(b)

Figure S1. The ESI-MS spectra in negative (**a**) and positive (**b**) modes for the ligand (**L**) solution in methanol.

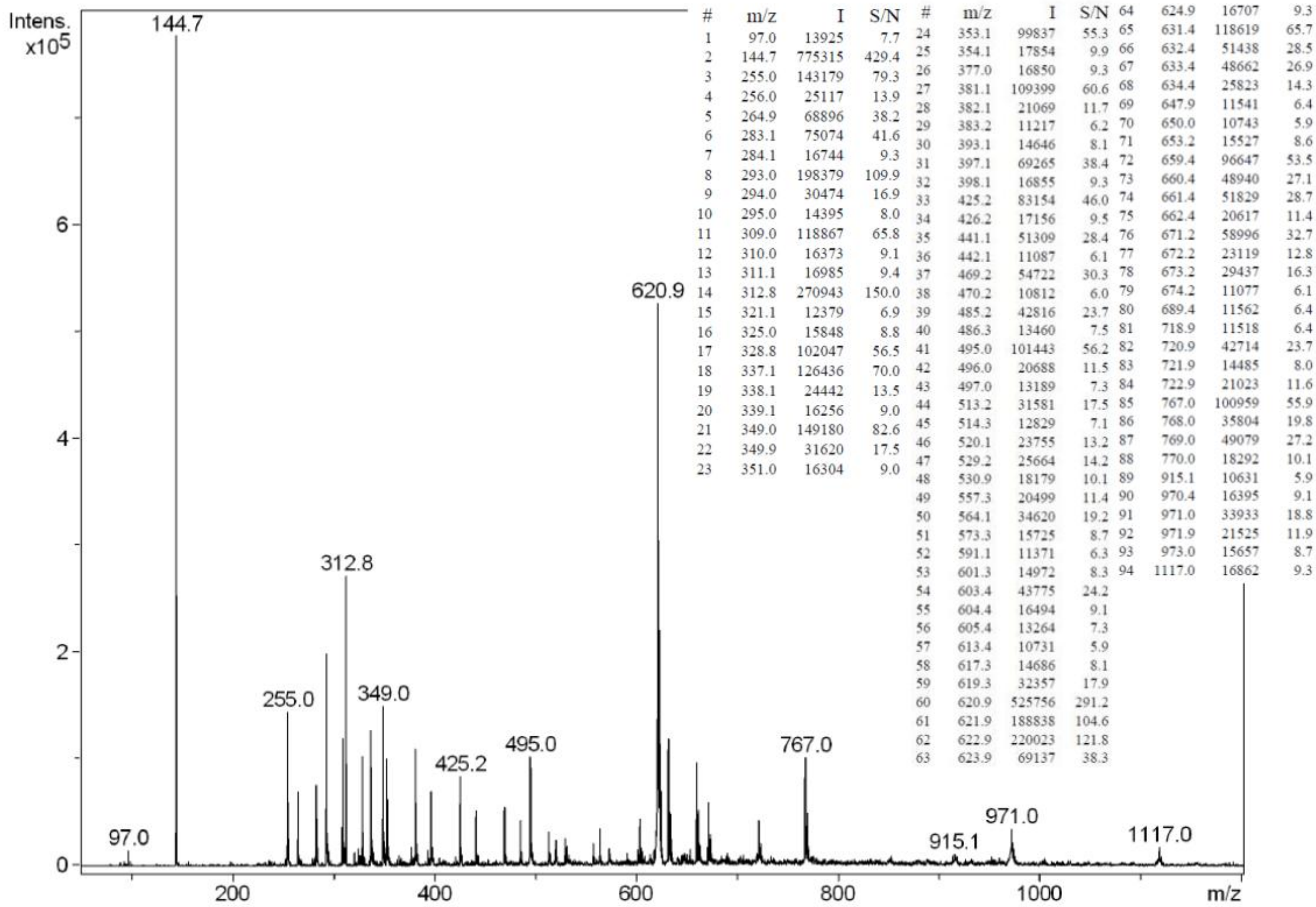

(a)

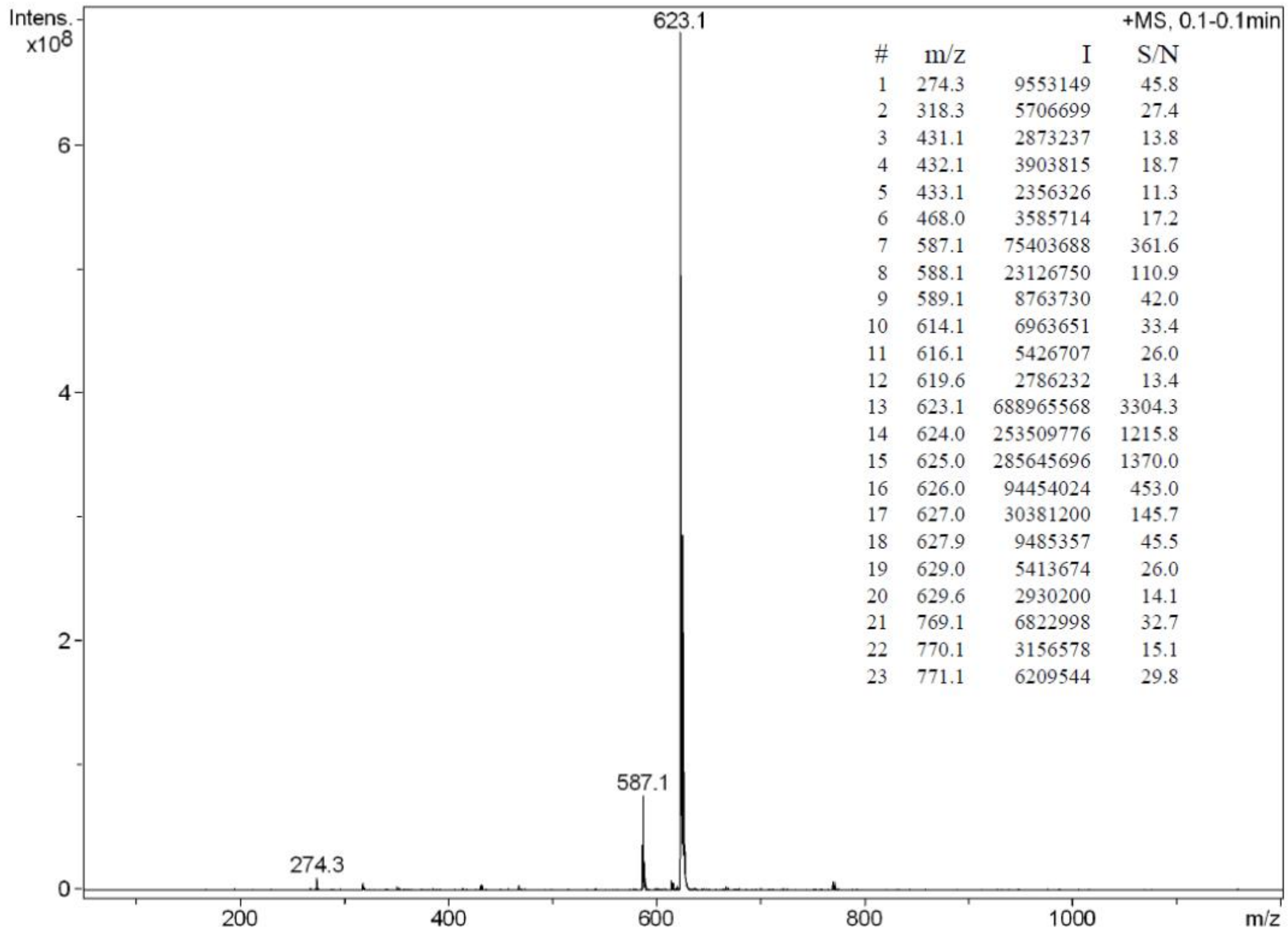

(b)

Figure S2. The ESI-MS spectra in negative (**a**) and positive (**b**) modes for the complex **1** solution in methanol.

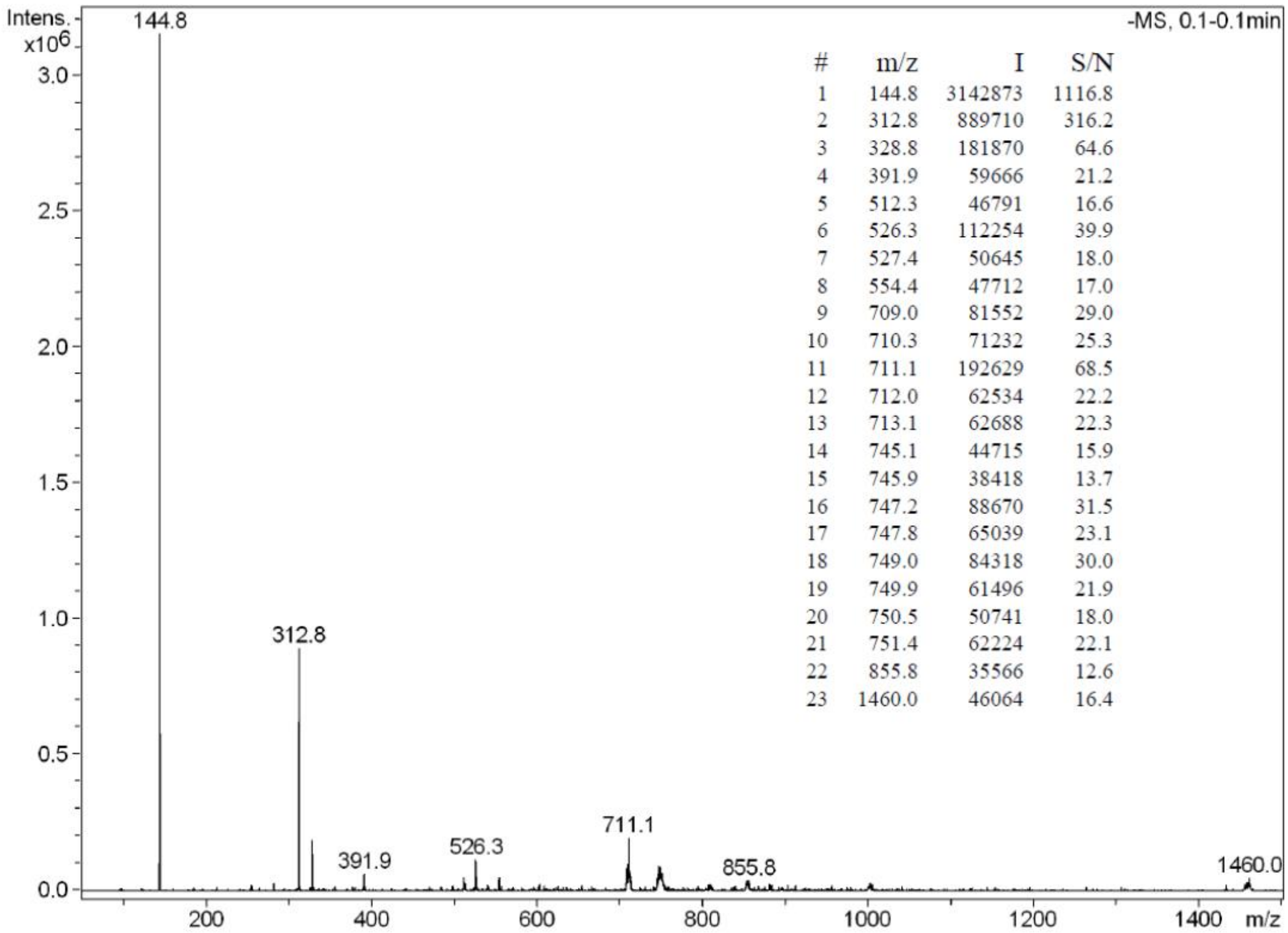

(a)

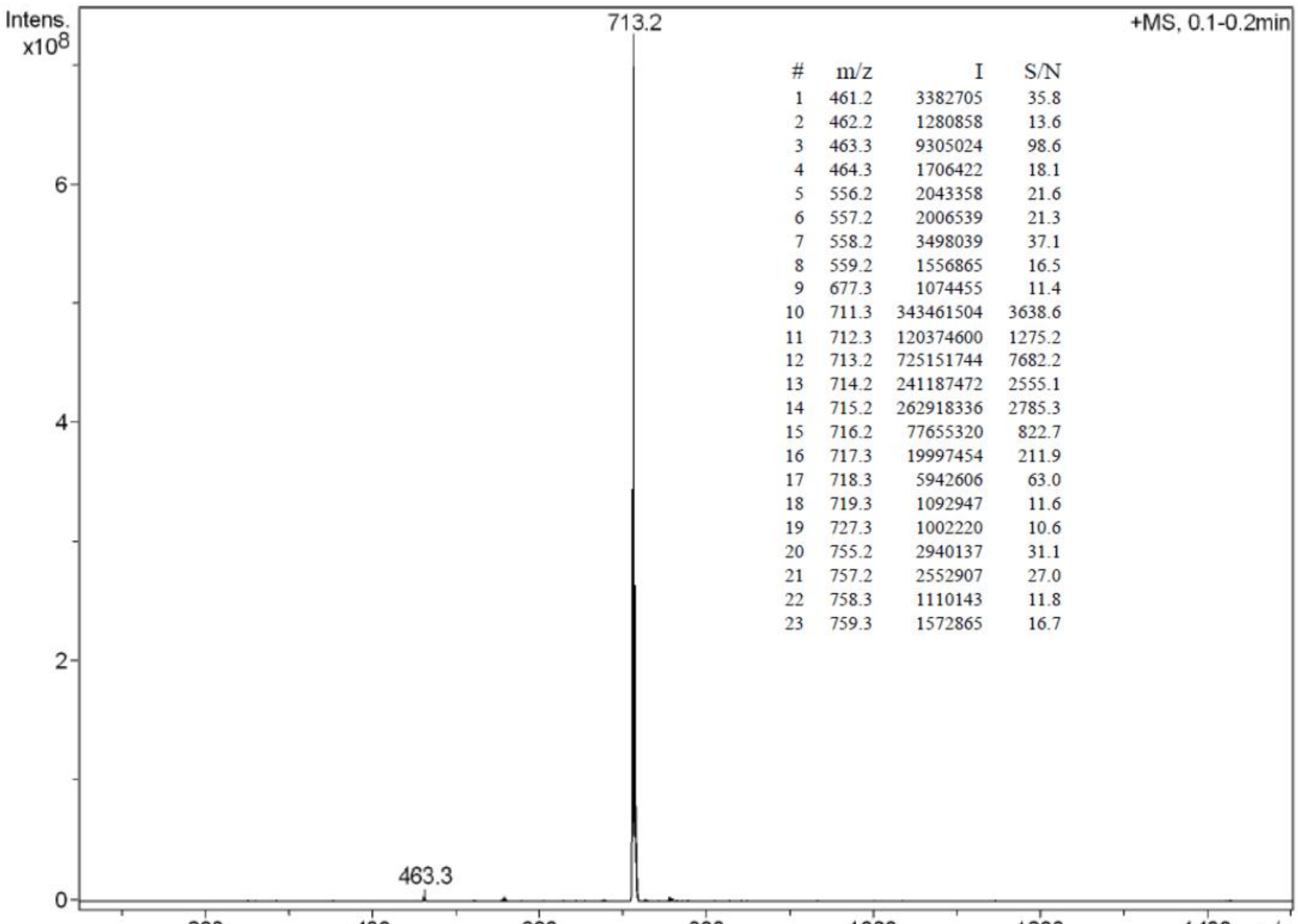

(b)

Figure S3. The ESI-MS spectra in negative (**a**) and positive (**b**) modes for the complex **2** solution in methanol.

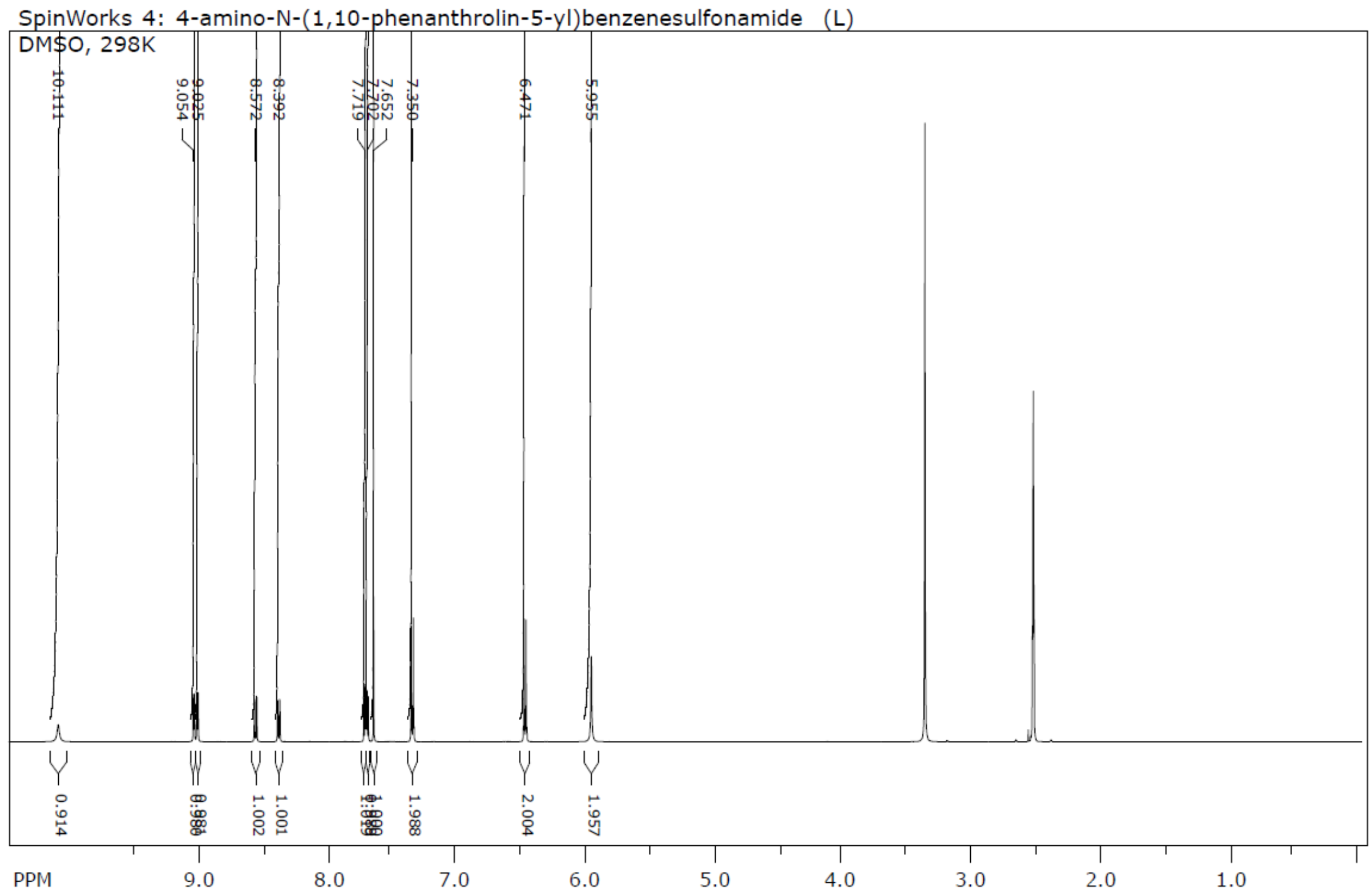

Figure S4. The ^{1}H NMR spectrum of ligand (**L**) in DMSO-d_6. The NMR signals are assigned in **Table S1** and in the section *Synthesis of the Ligand and Complexes* (manuscript).

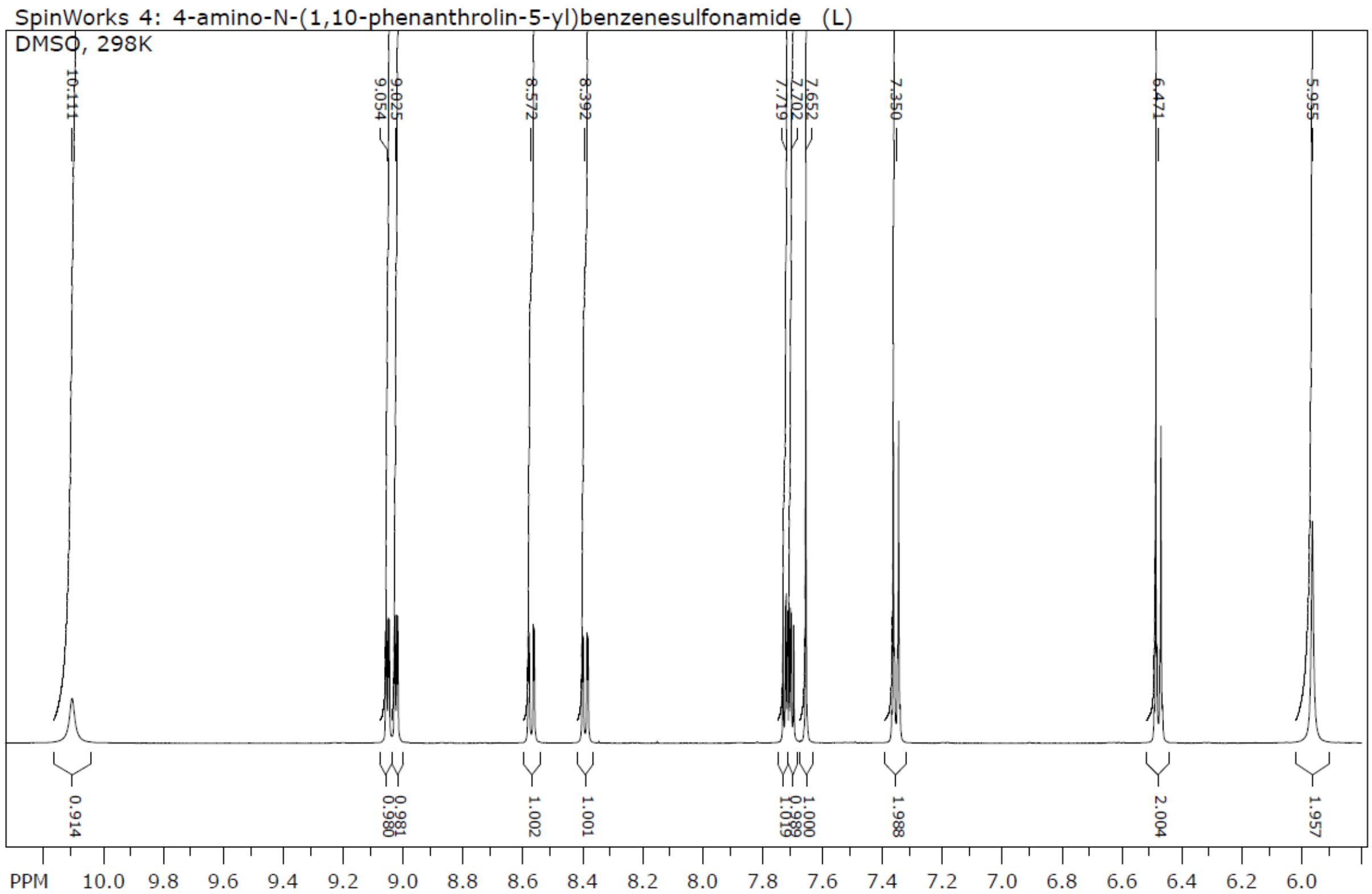

Figure S5. ^{1}H NMR spectrum (magnified) of ligand (**L**) in DMSO-d_6. The NMR signals are assigned in **Table S1** and in the section *Synthesis of the Ligand and Complexes* (manuscript).

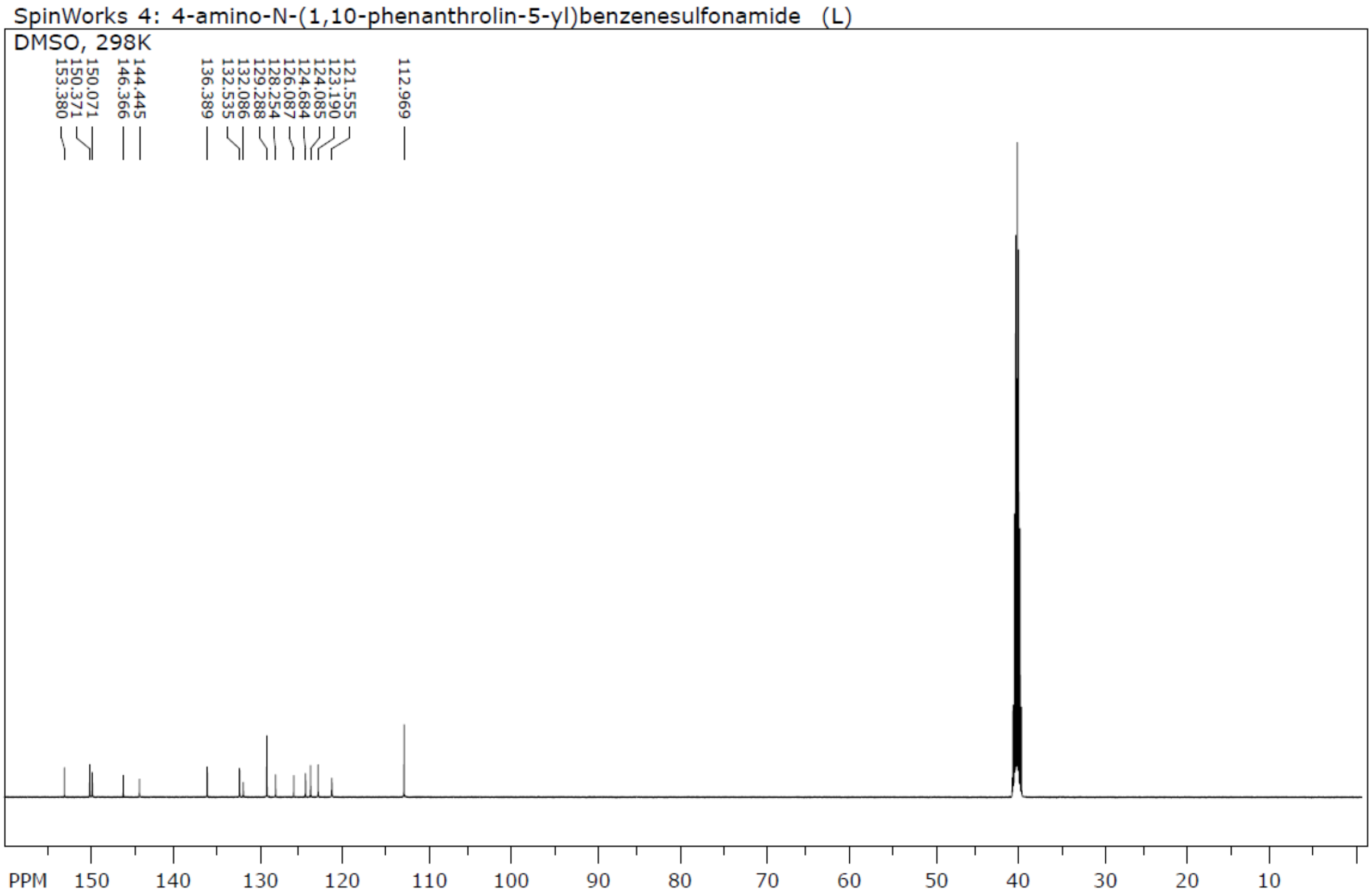

Figure S6. The ^{13}C NMR spectrum of ligand (**L**) in DMSO-d_6. The NMR signals are assigned in **Table S1** and in the section *Synthesis of the Ligand and Complexes* (manuscript).

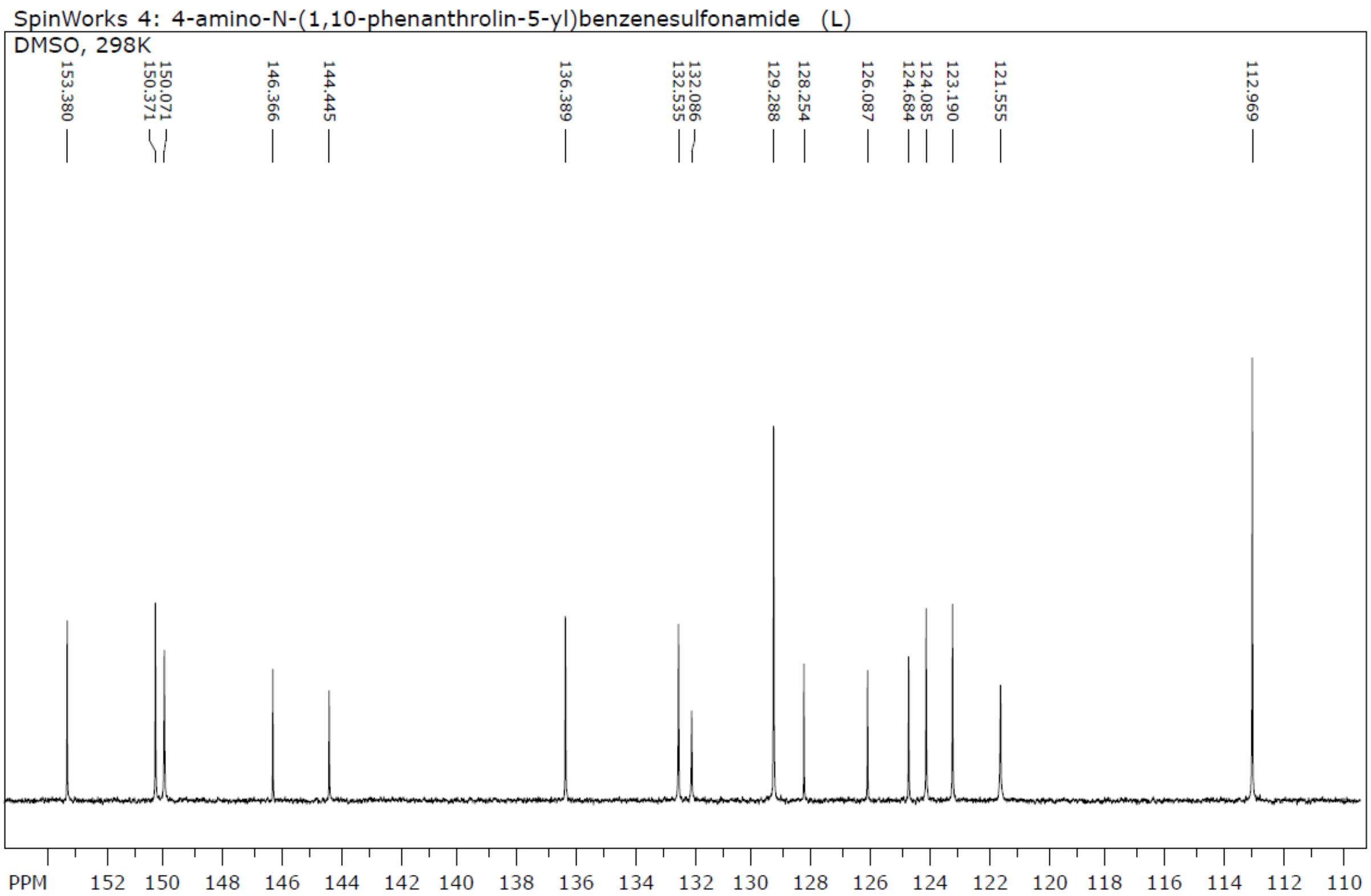

Figure S7. The ^{13}C NMR spectrum (magnified) of ligand (**L**) in DMSO-d_6. The NMR signals are assigned in **Table S1** and in the section *Synthesis of the Ligand and Complexes* (manuscript).

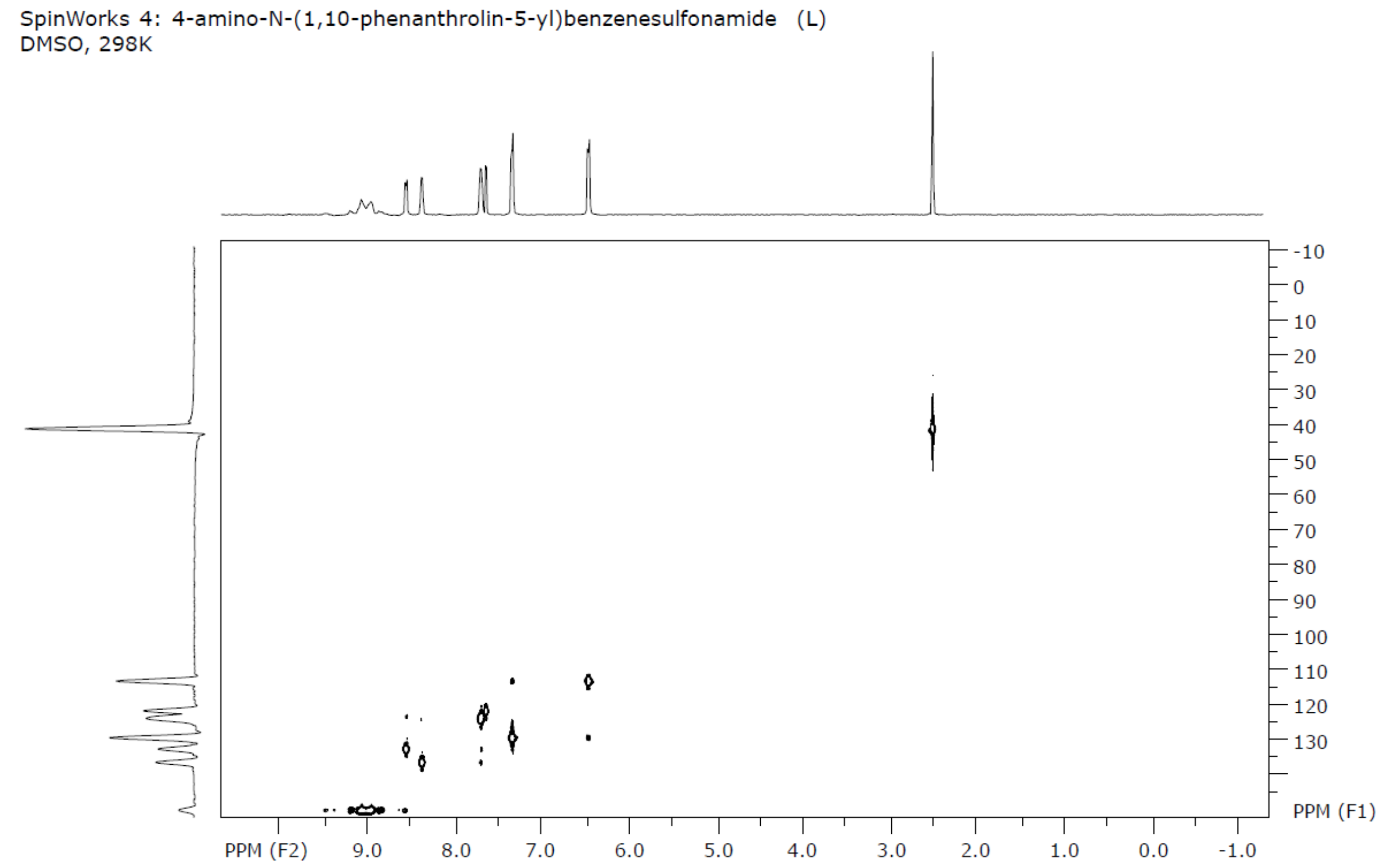

Figure. S8. The HSQC NMR correlation spectrum of ligand (**L**) in DMSO-d_6.

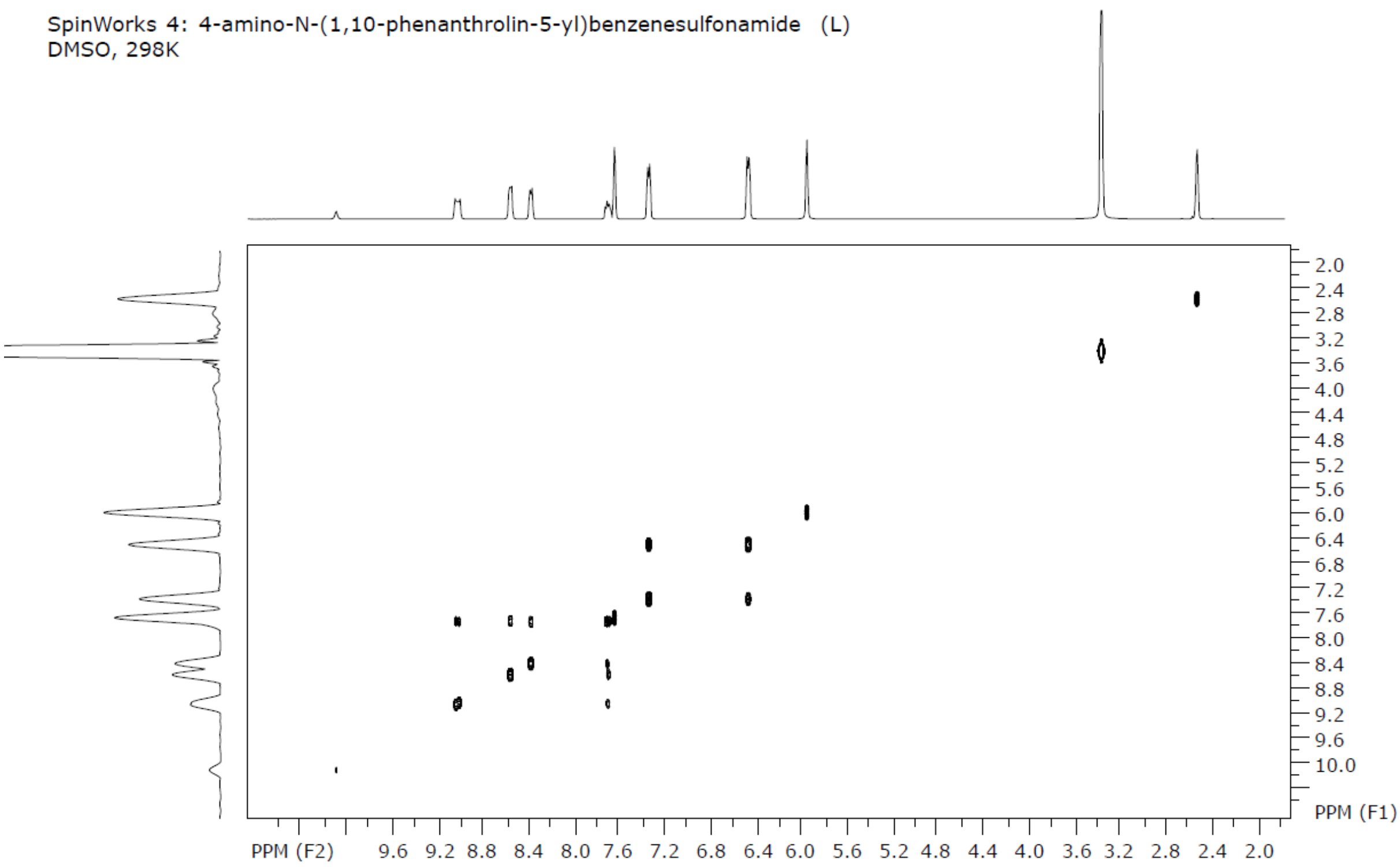

Figure S9. The COSY NMR correlation spectrum of ligand (**L**) in DMSO-d_6.

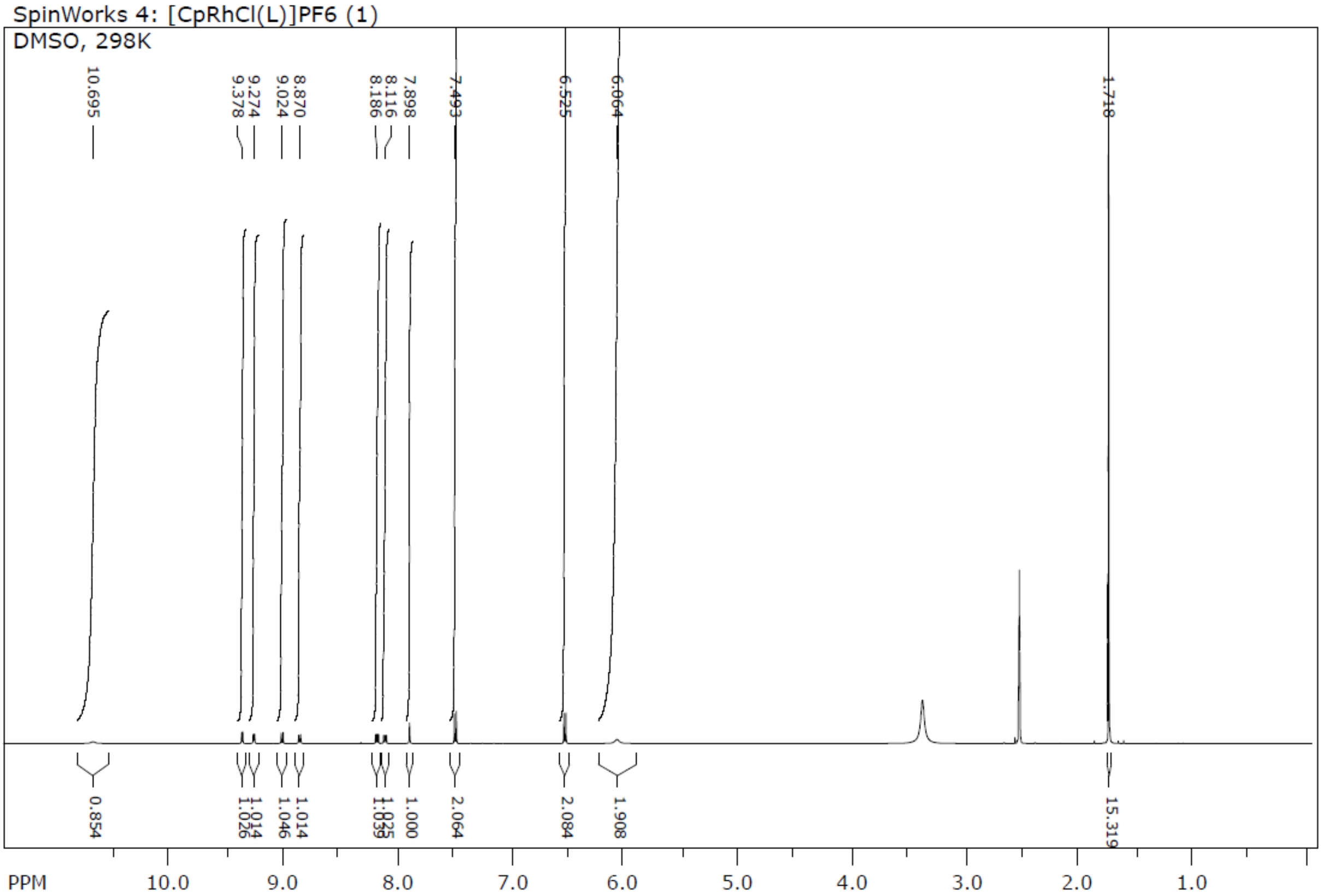

Figure S10. The ^{1}H NMR spectrum of complex **1** in DMSO-d_6. The NMR signals are assigned in **Table S1** and in the section *Synthesis of the Ligand and Complexes* (manuscript).

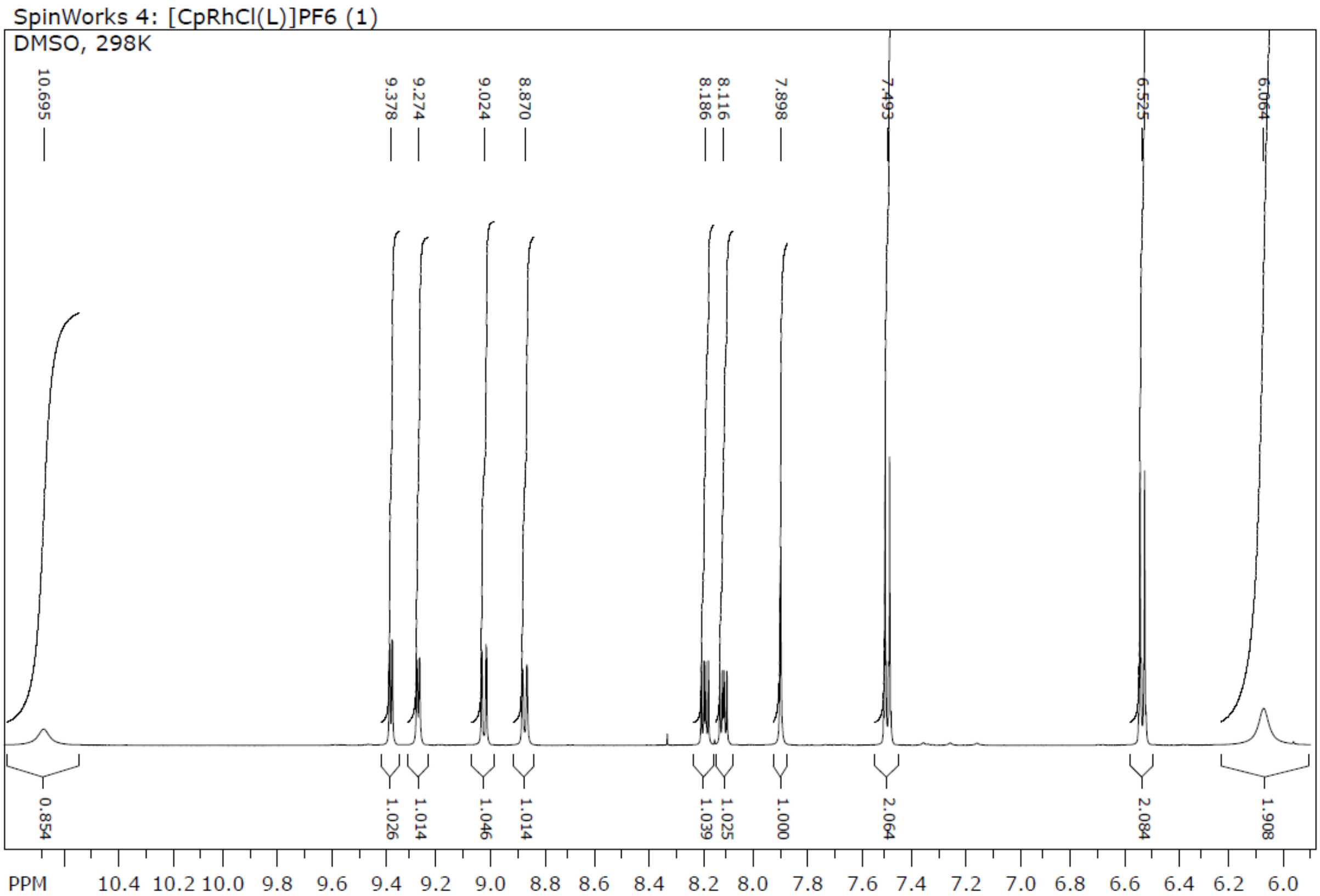

Figure S11. The ^{1}H NMR spectrum (magnified) of complex **1** in DMSO-d_6. The NMR signals are assigned in **Table S1** and in the section *Synthesis of the Ligand and Complexes* (manuscript).

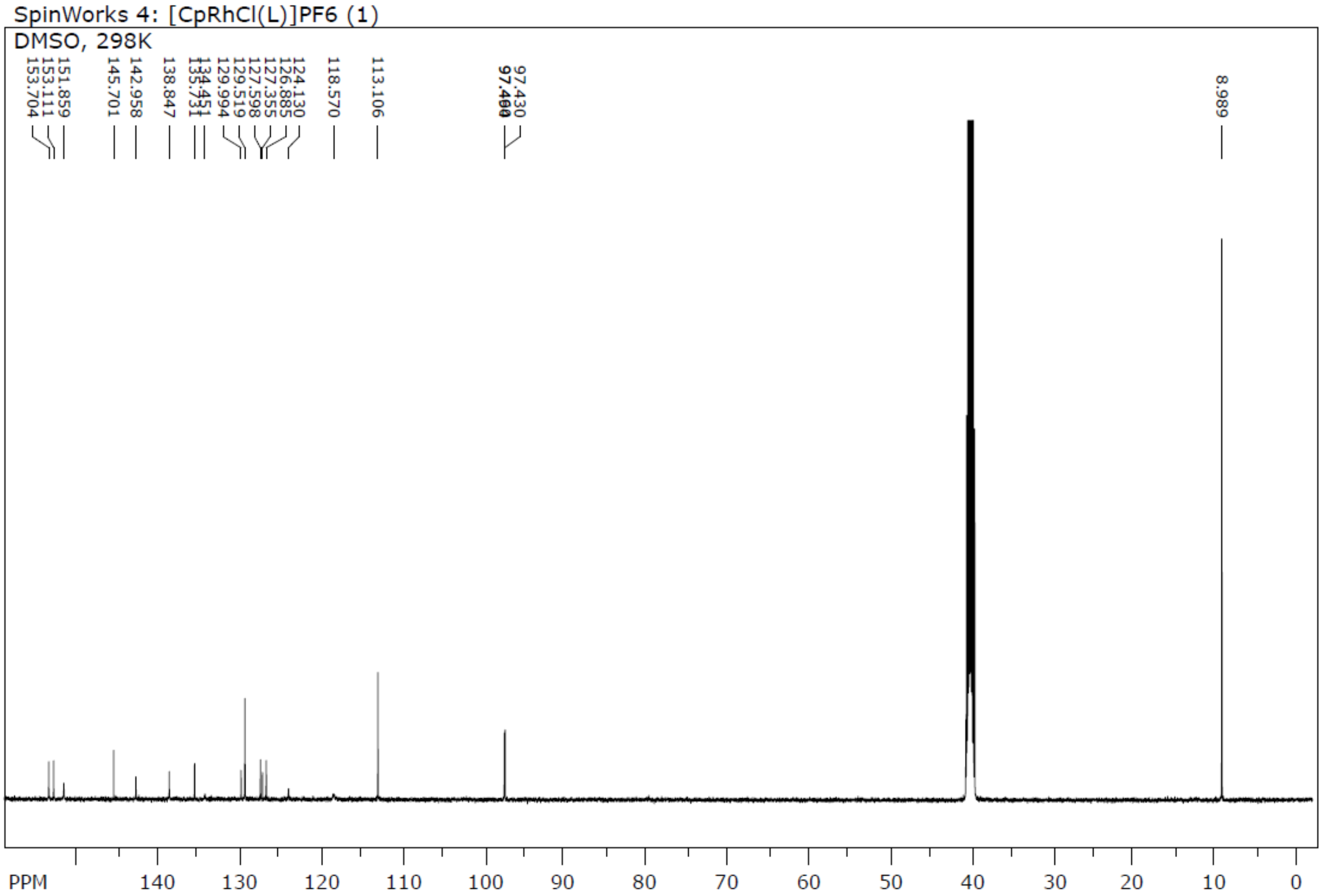

Figure S12. The ^{13}C NMR spectrum of complex **1** in DMSO-d_6. The NMR signals are assigned in **Table S1** and in the section *Synthesis of the Ligand and Complexes* (manuscript).

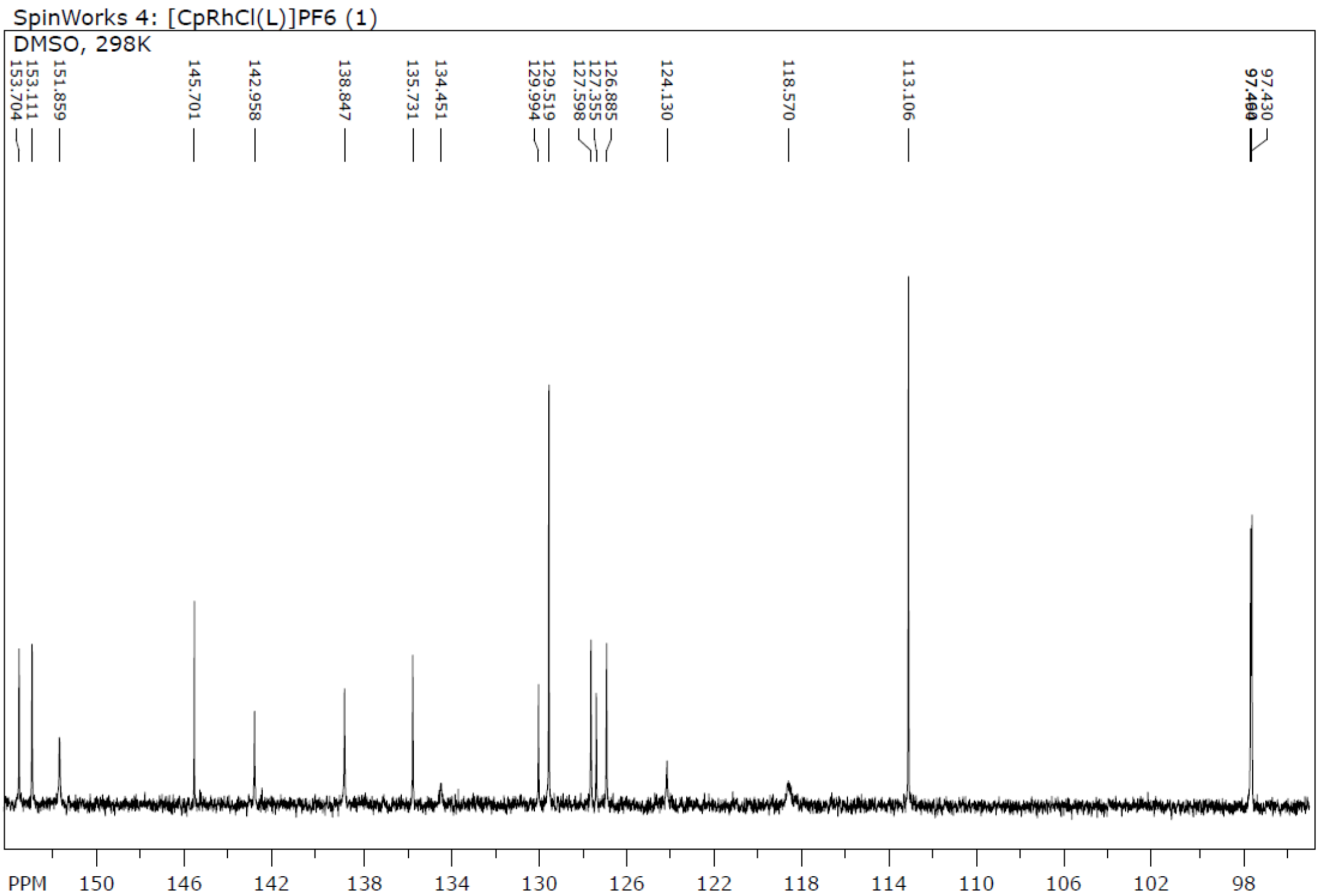

Figure S13. The ^{13}C NMR spectrum (magnified) of complex **1** in DMSO-d_6. The NMR signals are assigned in **Table S1** and in the section *Synthesis of the Ligand and Complexes* (manuscript).

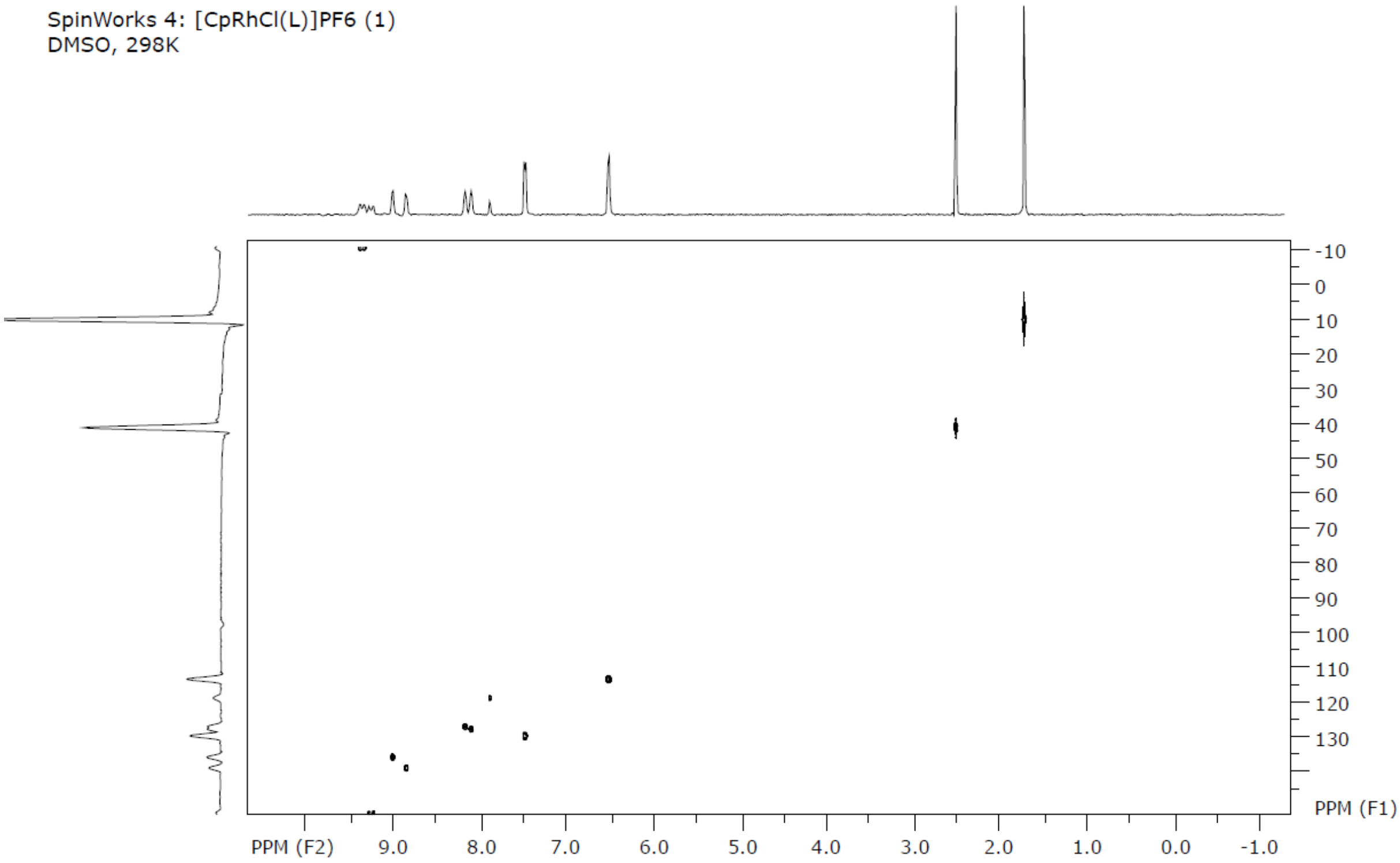

Figure S14. The HSQC NMR correlation spectrum of complex **1** in DMSO-d_6.

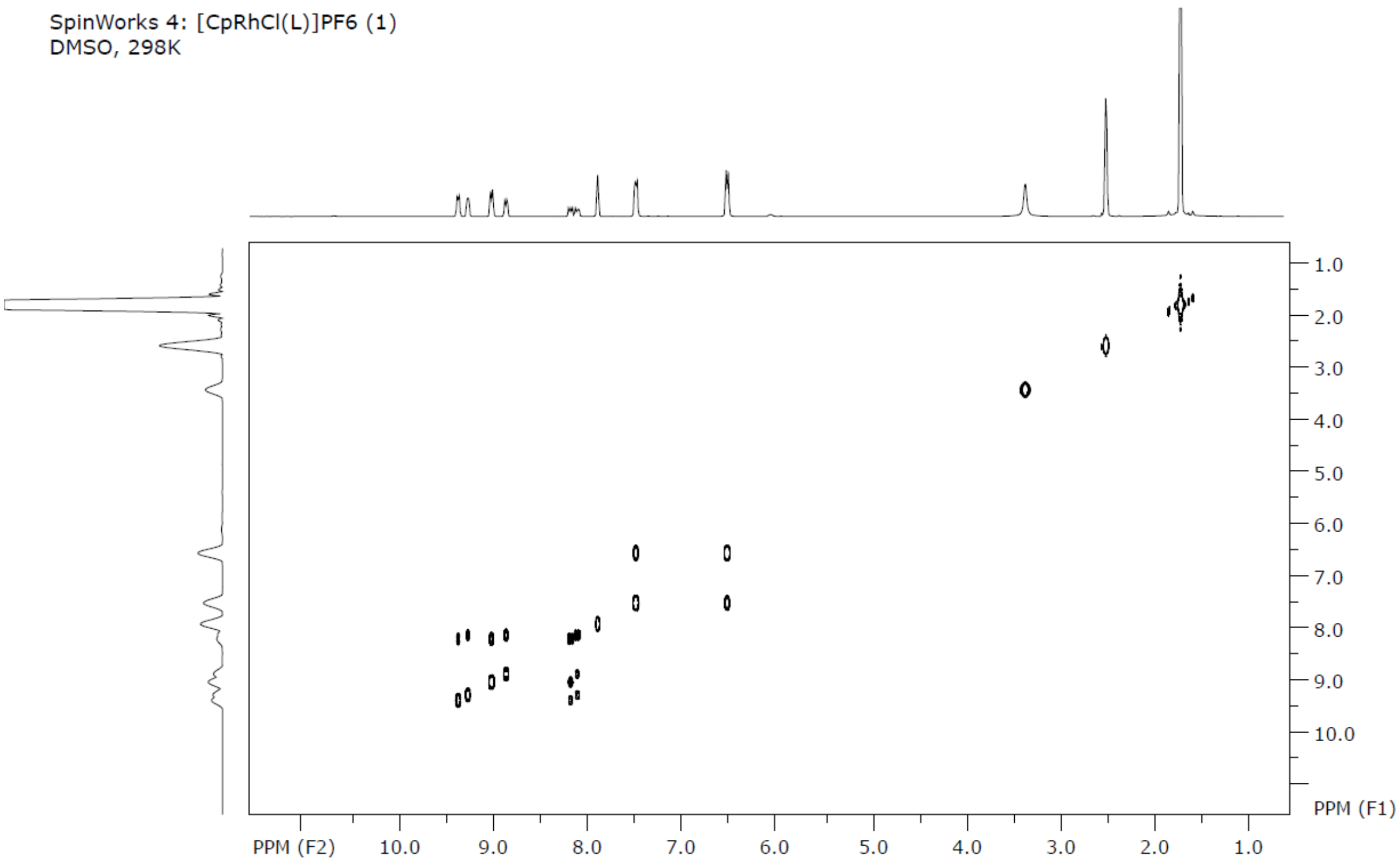

Figure S15. The COSY NMR correlation spectrum of complex **1** in DMSO-d_6.

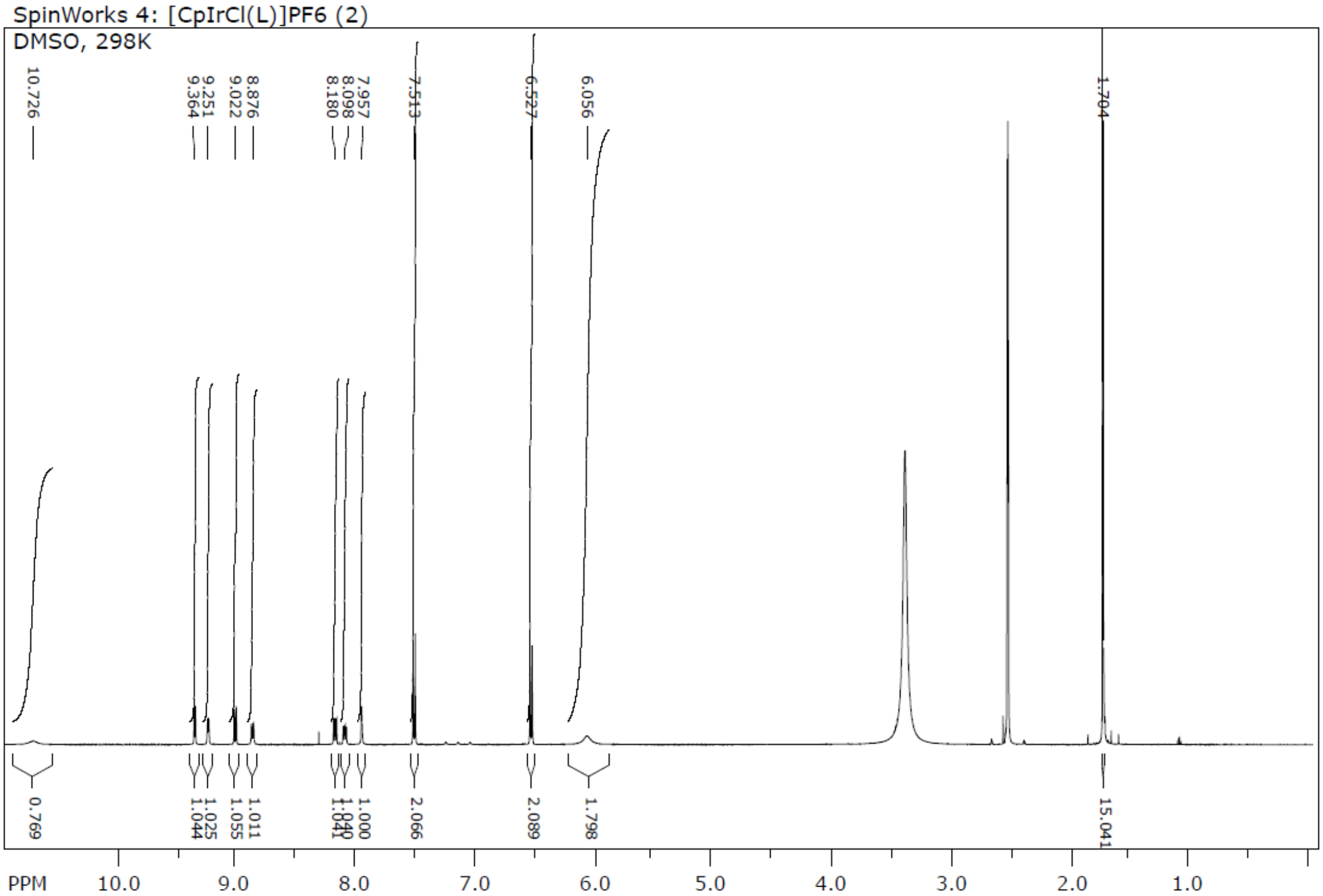

Figure S16. The ^{1}H NMR spectrum of complex **2** in DMSO-d$_6$. The NMR signals are assigned in **Table S1** and in the section *Synthesis of the Ligand and Complexes* (manuscript).

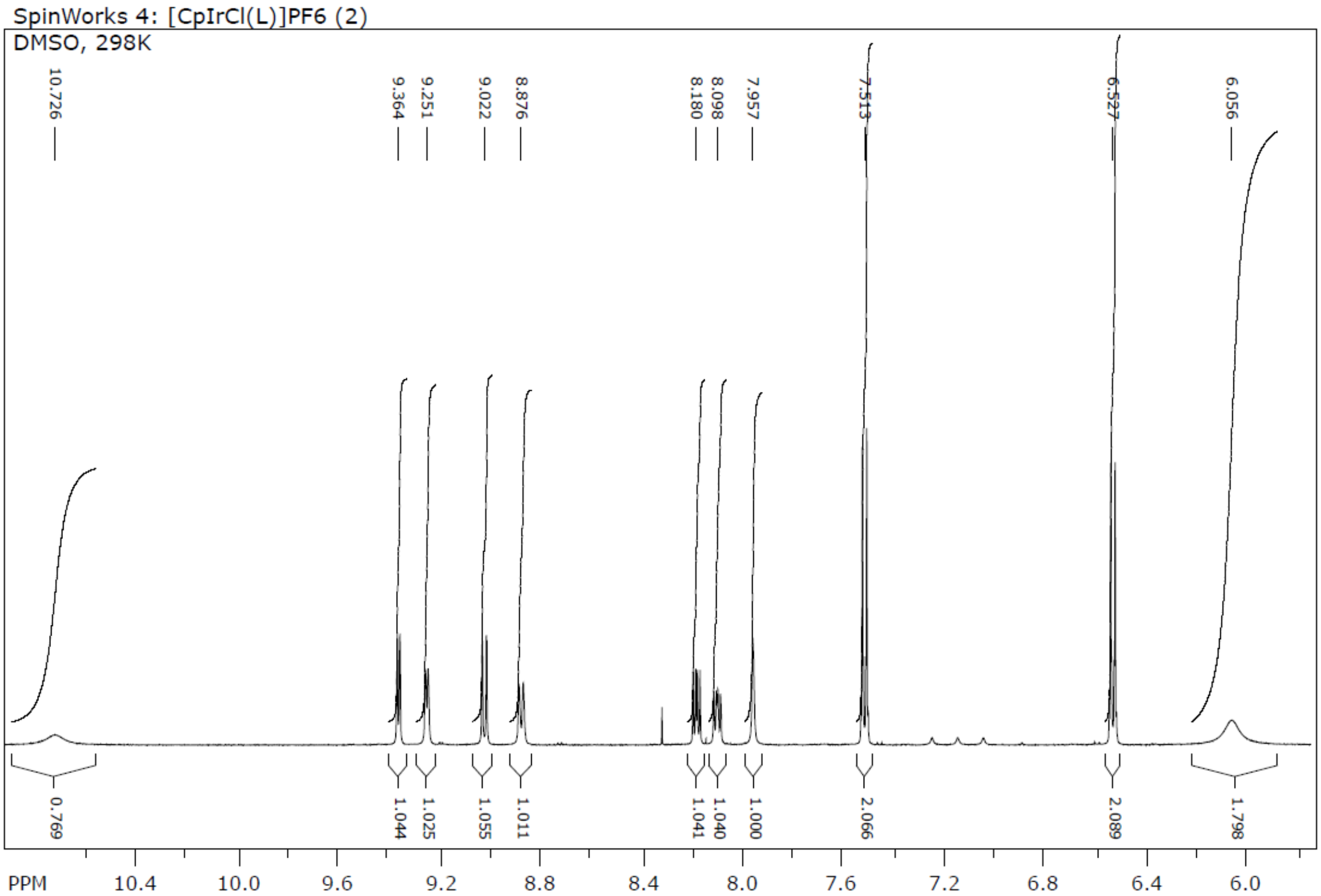

Figure S17. The ^{1}H NMR spectrum (magnified) of complex **2** in DMSO-d$_6$. The NMR signals are assigned in **Table S1** and in the section *Synthesis of the Ligand and Complexes* (manuscript).

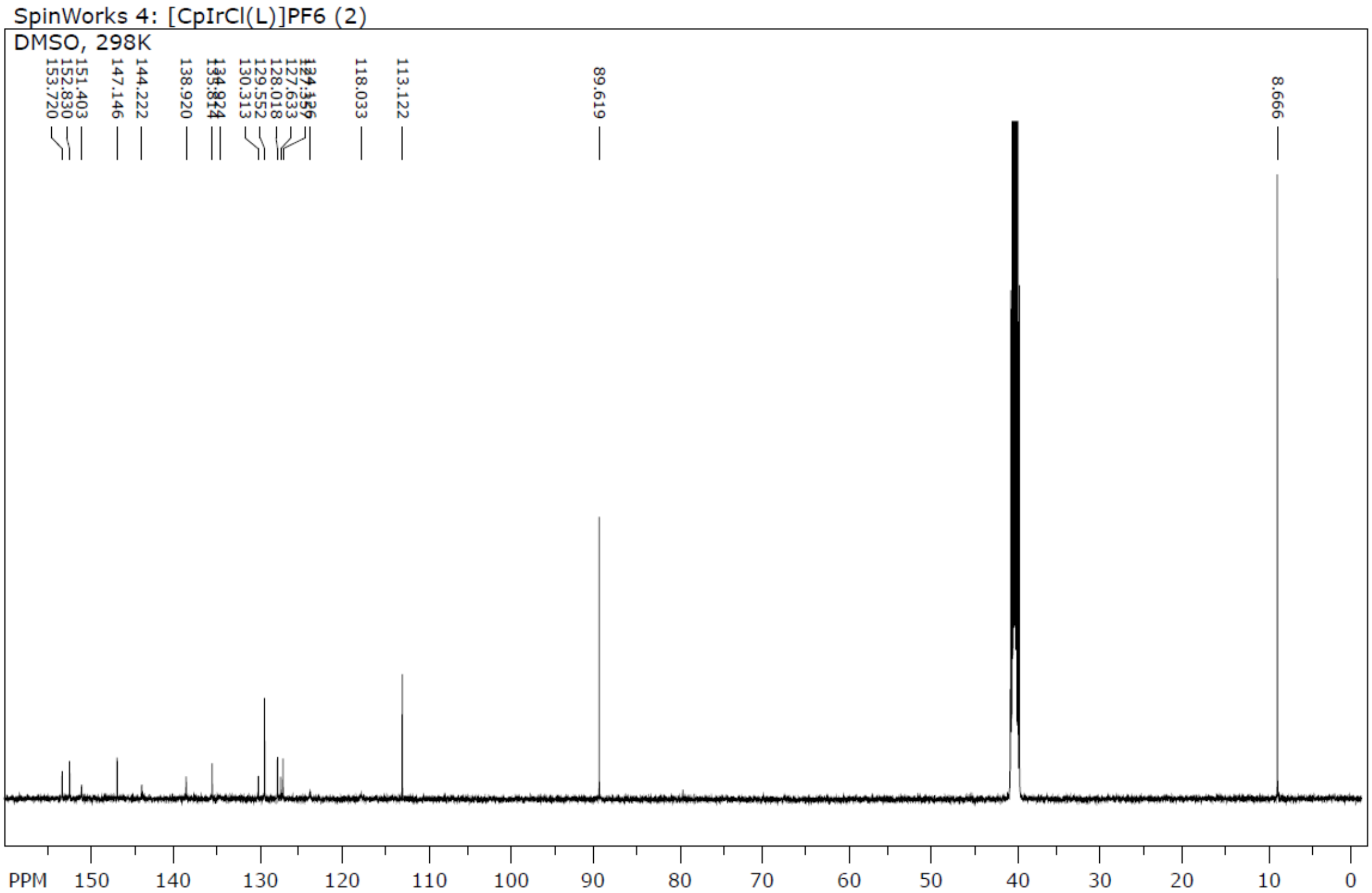

Figure S18. The ^{13}C NMR spectrum of complex **2** in DMSO-d_6. The NMR signals are assigned in **Table S1** and in the section *Synthesis of the Ligand and Complexes* (manuscript).

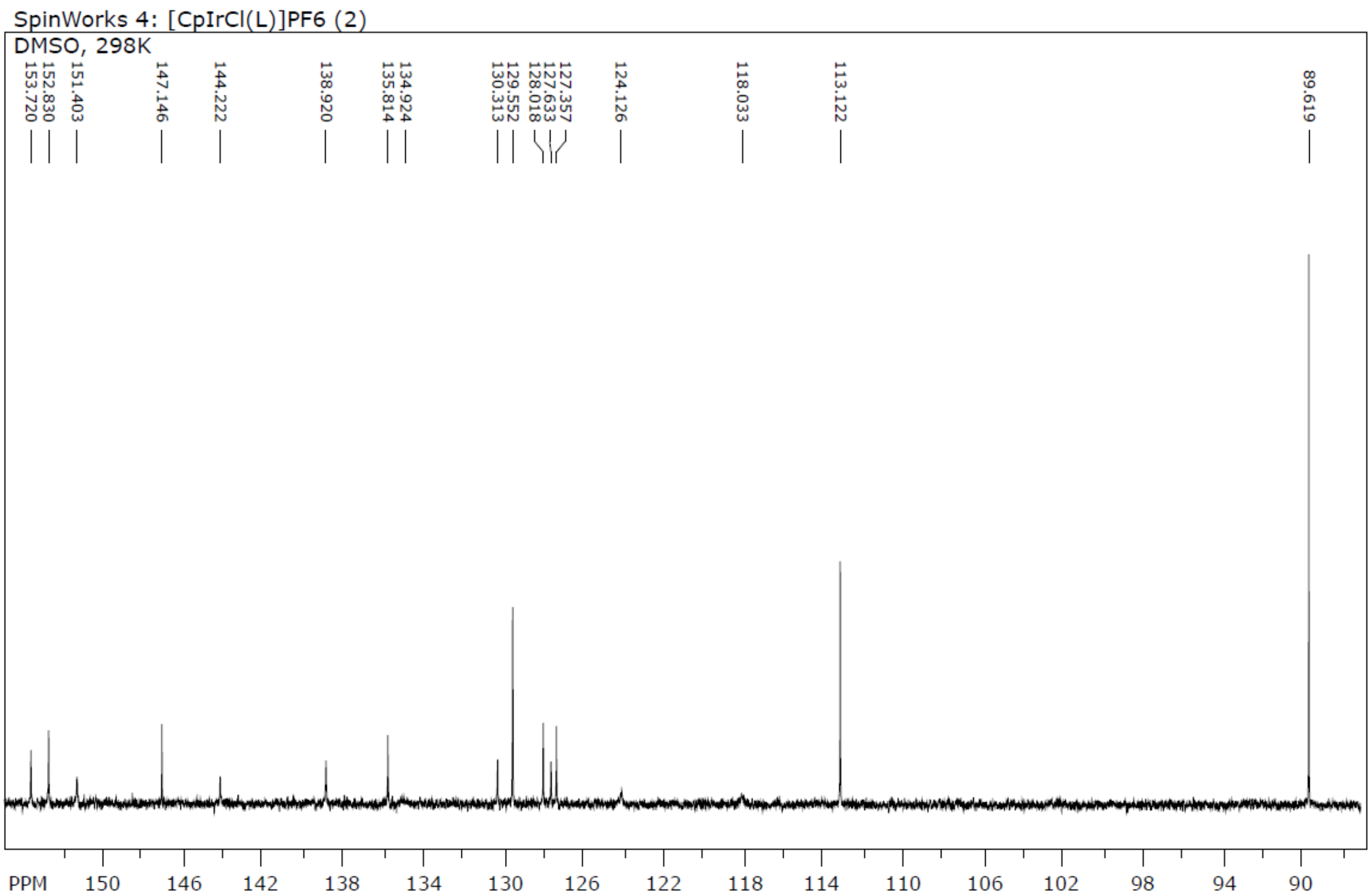

Figure S19. The ^{13}C NMR spectrum (magnified) of complex **2** in DMSO-d_6. The NMR signals are assigned in **Table S1** and in the section *Synthesis of the Ligand and Complexes* (manuscript).

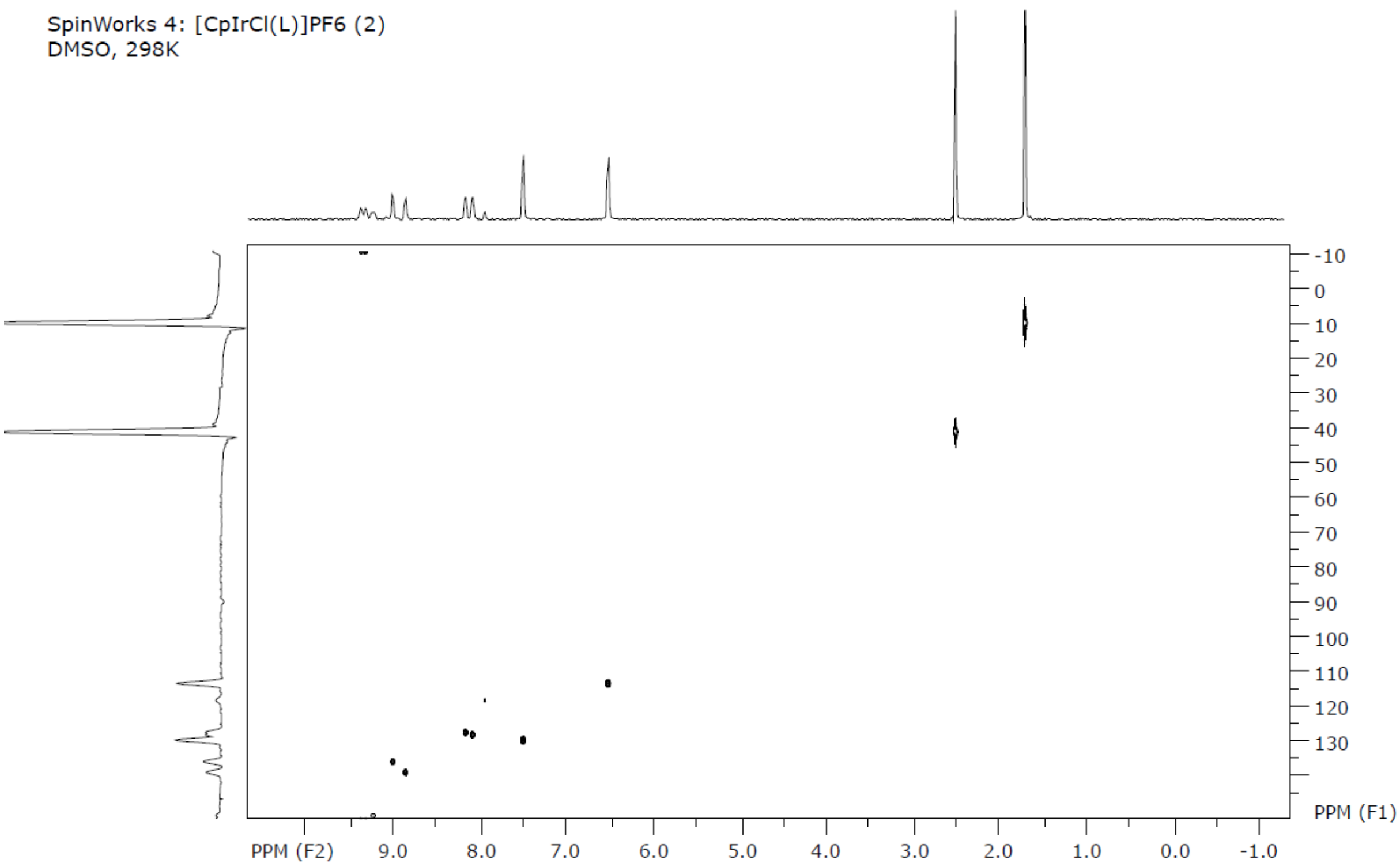

Figure S20. The HSQC NMR correlation spectrum of complex **2** in DMSO-d_6.

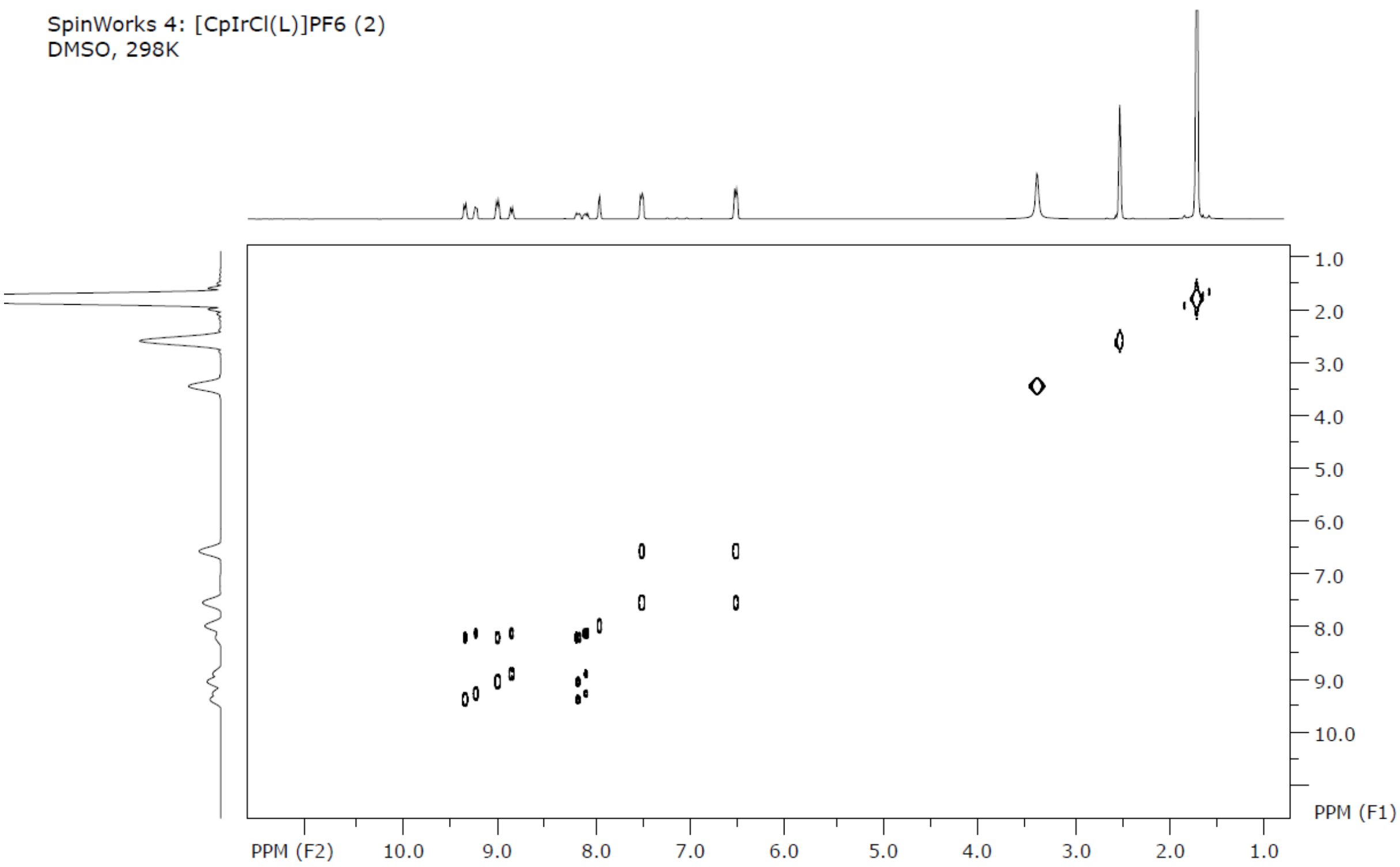

Figure S21. The COSY NMR correlation spectrum of complex **2** in DMSO-d_6.

Table S1.
Atom and ring markers used for NMR spectroscopic determinations for **L** (according to **Figs. S4-S9**) and complexes **1** (according to **Figs. S10-S15**) and **2** (according to **Figs. S16-S21**), along with the assigned shifts (ppm).

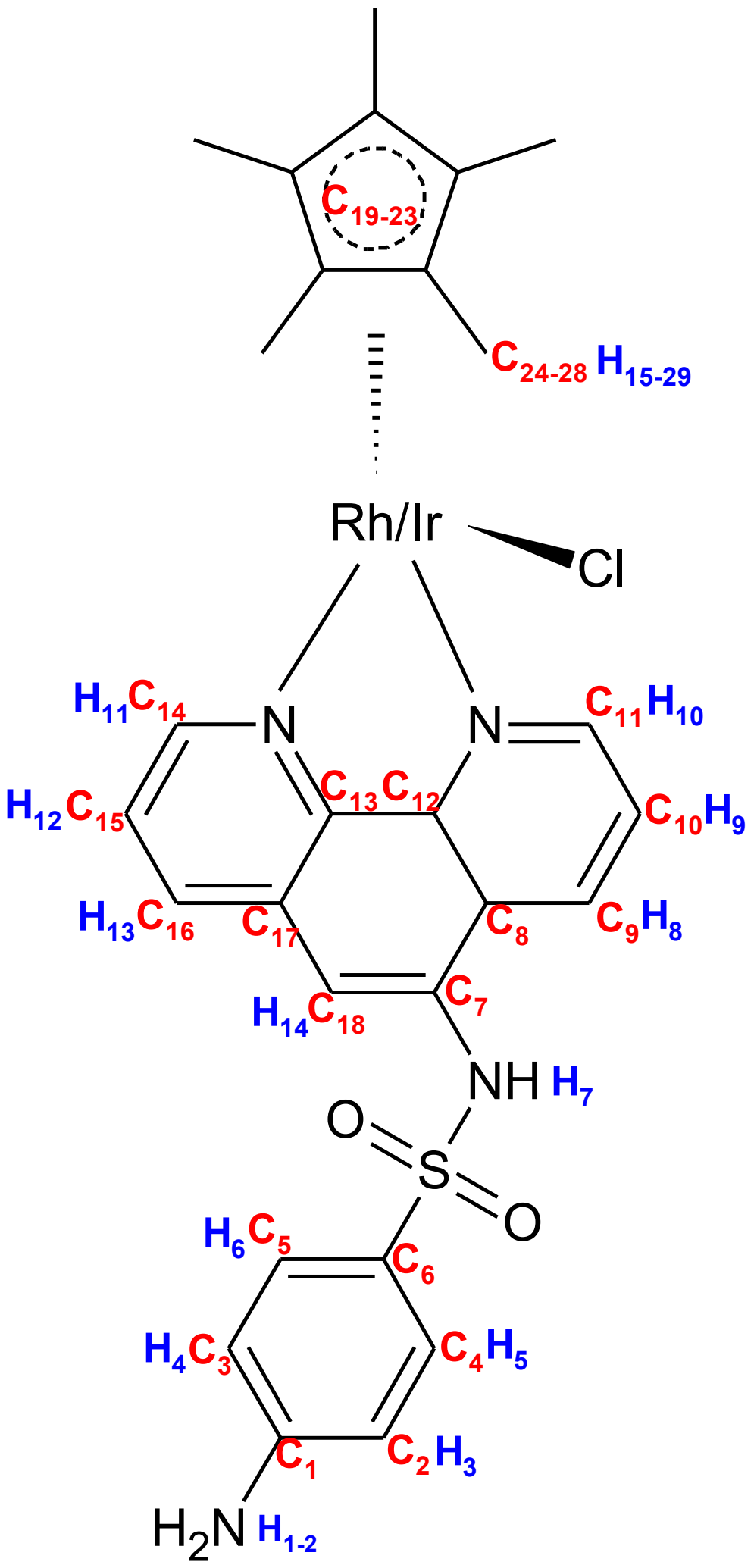

Atom labels	L	1 (Rh)	2 (Ir)
H1/H2	5.96	6.06	6.06
H3/H4	6.50-6.44	6.56-6.49	6.56-6.50
H5/H6	7.38-7.32	7.53-7.46	7.55-7.48
H7	10.11	10.70	10.73
H8	8.57	9.02	9.02
H9	7.72	8.19	8.18
H10	9.05	9.38	9.36
H11	9.03	9.27	9.25
H12	7.70	8.12	8.10
H13	8.39	8.87	8.88
H14	7.65	7.90	7.96
H15-H29	-	1.72	1.70
C1	124.09	124.13	124.13
C2/C3	112.97	113.11	113.12
C4/C5	129.29	129.52	129.55
C6	153.38	153.70	153.72
C7	132.09	134.45	134.92
C8	126.09	127.36	127.63
C9	132.54	135.73	135.81
C10	124.68	126.89	127.36
C11	150.37	153.11	152.83
C12	146.37	145.70	147.15
C13	144.45	142.96	144.22
C14	150.07	151.86	151.40
C15	123.19	127.60	128.02
C16	136.39	138.85	138.92
C17	128.25	129.99	130.31
C18	121.56	118.57	118.03
C19-C23	-	97.46	89.62
C24-C28	-	8.99	8.67

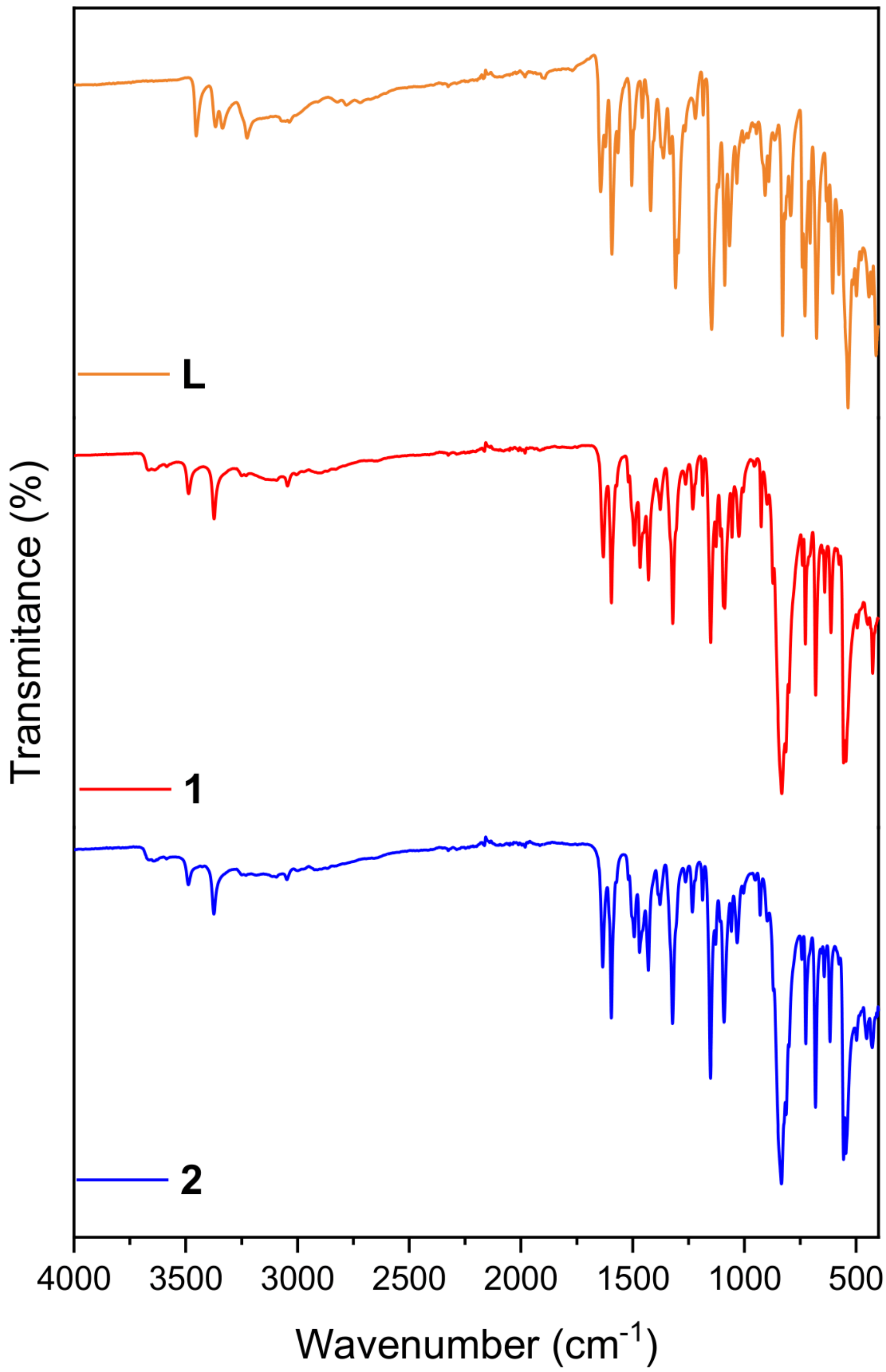

Figure S22. Experimental FT-IR spectra of compound **L** and coordination compounds **1** and **2**. The characteristic FT-IR bands were identified and discussed in the *RESULTS AND DISCUSSION* section, under the subsection titled *FT-IR Spectroscopy* (manuscript).

Table S2.
Bond lengths [Å] and angles [º] for **L** obtained from X-ray analysis. Atom numbering as in **Fig. 1** (manuscript).

Bond lengths [Å]		Bond angles [°]	
S(1)-O(2)	1.434(2)	O(2)-S(1)-O(1)	118.61(12)
S(1)-O(1)	1.438(2)	O(2)-S(1)-N(2)	105.55(12)
S(1)-N(2)	1.650(2)	O(1)-S(1)-N(2)	107.27(12)
S(1)-C(1)	1.742(3)	O(2)-S(1)-C(1)	110.73(12)
N(4)-C(15)	1.322(4)	O(1)-S(1)-C(1)	107.68(13)
N(4)-C(14)	1.362(3)	N(2)-S(1)-C(1)	106.29(12)
N(3)-C(12)	1.320(4)	C(15)-N(4)-C(14)	117.7(2)
N(3)-C(13)	1.369(4)	C(12)-N(3)-C(13)	117.4(2)
N(2)-C(7)	1.440(3)	C(7)-N(2)-S(1)	120.68(19)
N(1)-C(4)	1.363(4)	C(8)-C(7)-N(2)	121.2(2)
C(7)-C(8)	1.341(4)	C(8)-C(7)-C(18)	120.9(2)
C(7)-C(18)	1.447(4)	N(2)-C(7)-C(18)	117.6(2)
C(3)-C(2)	1.383(4)	C(2)-C(3)-C(4)	120.9(3)
C(3)-C(4)	1.414(4)	C(7)-C(8)-C(9)	121.3(2)
C(8)-C(9)	1.438(4)	C(10)-C(9)-C(13)	118.5(2)
C(9)-C(10)	1.397(4)	C(10)-C(9)-C(8)	121.7(2)
C(9)-C(13)	1.410(4)	C(13)-C(9)-C(8)	119.8(3)
C(4)-C(5)	1.397(4)	N(1)-C(4)-C(5)	121.0(3)
C(14)-C(18)	1.420(4)	N(1)-C(4)-C(3)	120.4(3)
C(14)-C(13)	1.449(4)	C(5)-C(4)-C(3)	118.6(3)
C(18)-C(17)	1.401(4)	N(4)-C(14)-C(18)	122.0(2)
C(2)-C(1)	1.401(4)	N(4)-C(14)-C(13)	118.7(2)
C(10)-C(11)	1.378(4)	C(18)-C(14)-C(13)	119.3(2)
C(17)-C(16)	1.369(4)	C(17)-C(18)-C(14)	117.8(2)
C(6)-C(5)	1.391(4)	C(17)-C(18)-C(7)	123.0(2)
C(6)-C(1)	1.396(4)	C(14)-C(18)-C(7)	119.2(2)
C(15)-C(16)	1.406(4)	C(3)-C(2)-C(1)	119.6(3)
C(11)-C(12)	1.406(4)	C(11)-C(10)-C(9)	119.2(3)
		N(3)-C(13)-C(9)	122.2(3)
		N(3)-C(13)-C(14)	118.6(2)
		C(9)-C(13)-C(14)	119.2(2)
		C(16)-C(17)-C(18)	119.9(3)
		C(5)-C(6)-C(1)	119.7(3)
		N(4)-C(15)-C(16)	124.4(3)
		C(17)-C(16)-C(15)	118.0(3)
		C(6)-C(1)-C(2)	120.3(3)
		C(6)-C(1)-S(1)	120.4(2)
		C(2)-C(1)-S(1)	119.3(2)
		C(10)-C(11)-C(12)	118.4(3)
		C(6)-C(5)-C(4)	120.9(3)
		N(3)-C(12)-C(11)	124.3(3)

Table S3.
The geometry of hydrogen bonds in the crystal structure of **L**. Atom numbering as in **Fig. 1** (manuscript).

D–H···A	D–H [Å]	H···A [Å]	D···A [Å]	D–H···A [º]
N2–H2···N4^{i}	0.88	2.352(8)	3.080(8)	140.27(0)
N1–H1A···N3^{ii}	0.862(2)	2.451(15)	3.071(20)	129.27(1)
C16–H16···O2^{iii}	0.950(4)	2.366(5)	3.175(1)	142.82(1)

Symmetry codes: (i) 0.5+x,1.5-y,0.5+z; (ii) 1+x,y,1+z; (iii) -x,1-y,1-z.

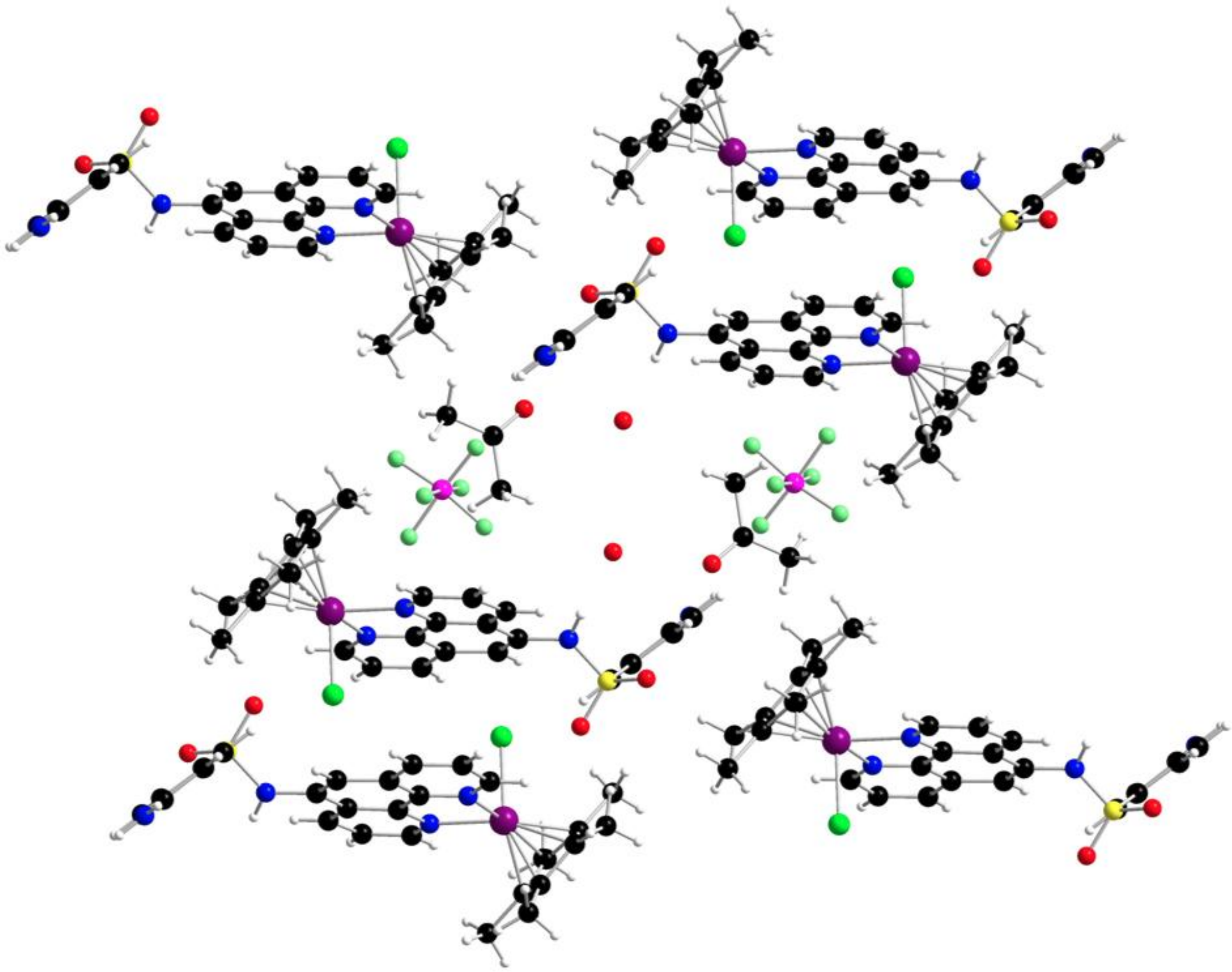

Figure S23. The X-ray crystal packing of complex **2** along *a* axis.

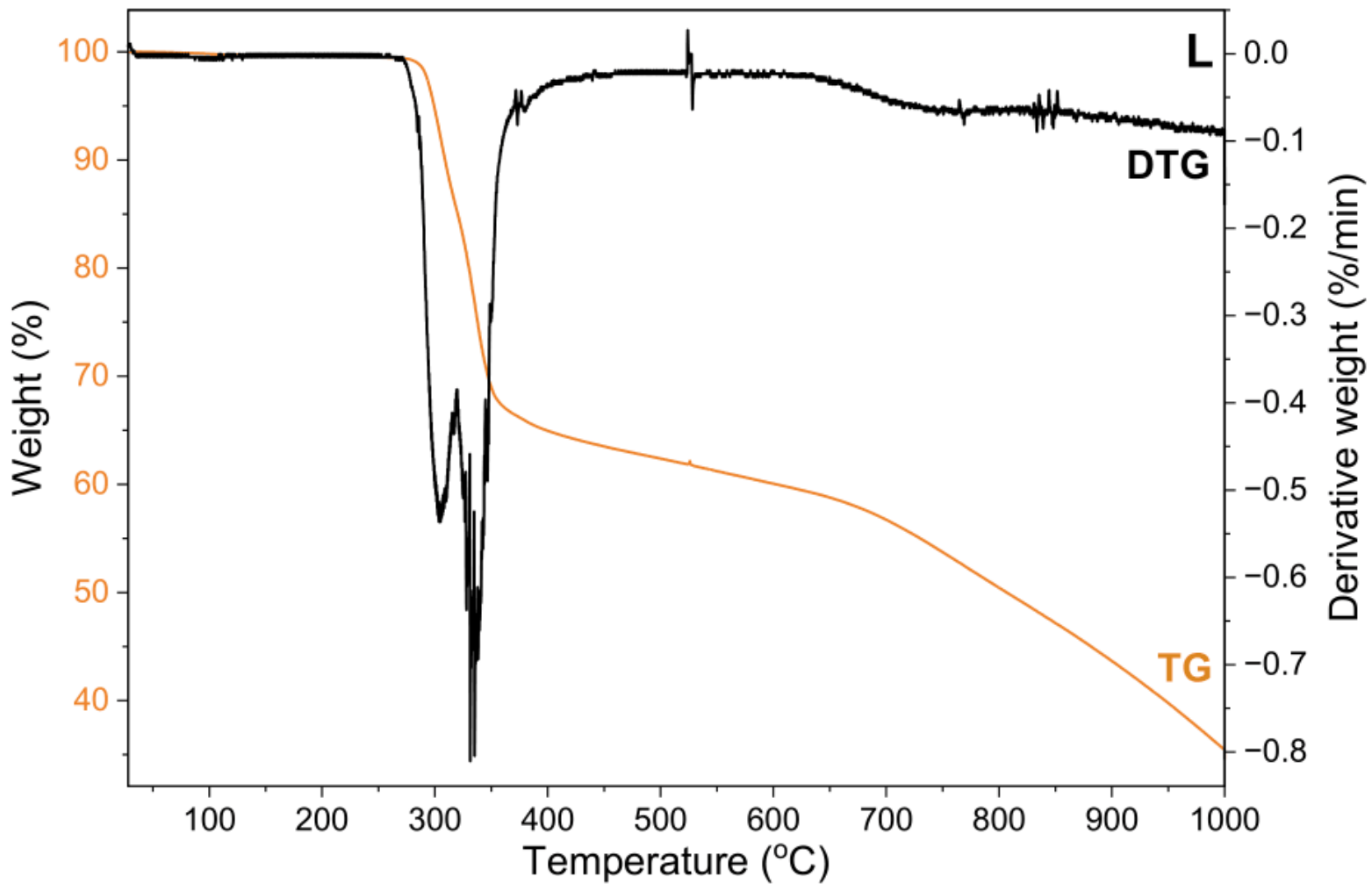

(a)

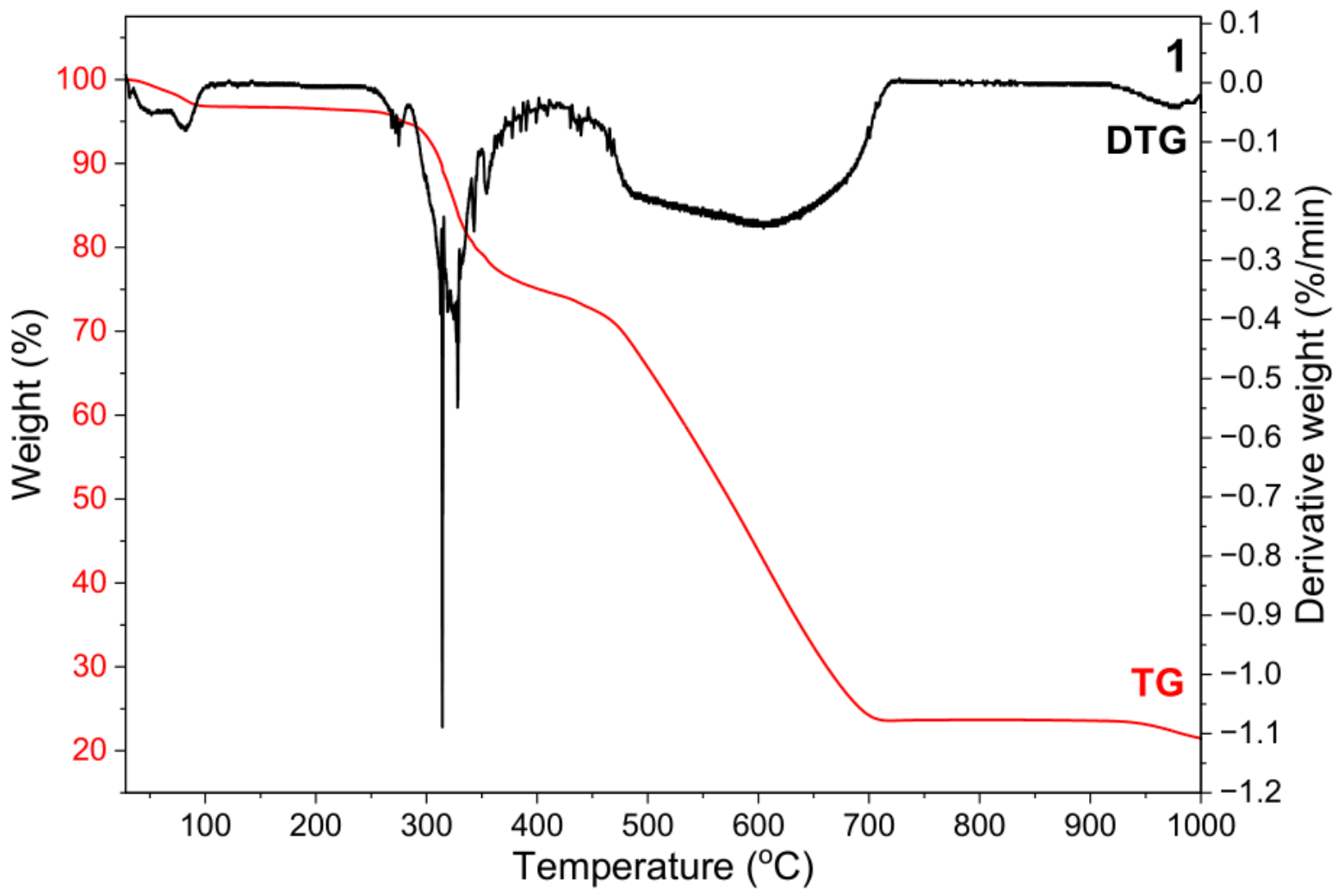

(b)

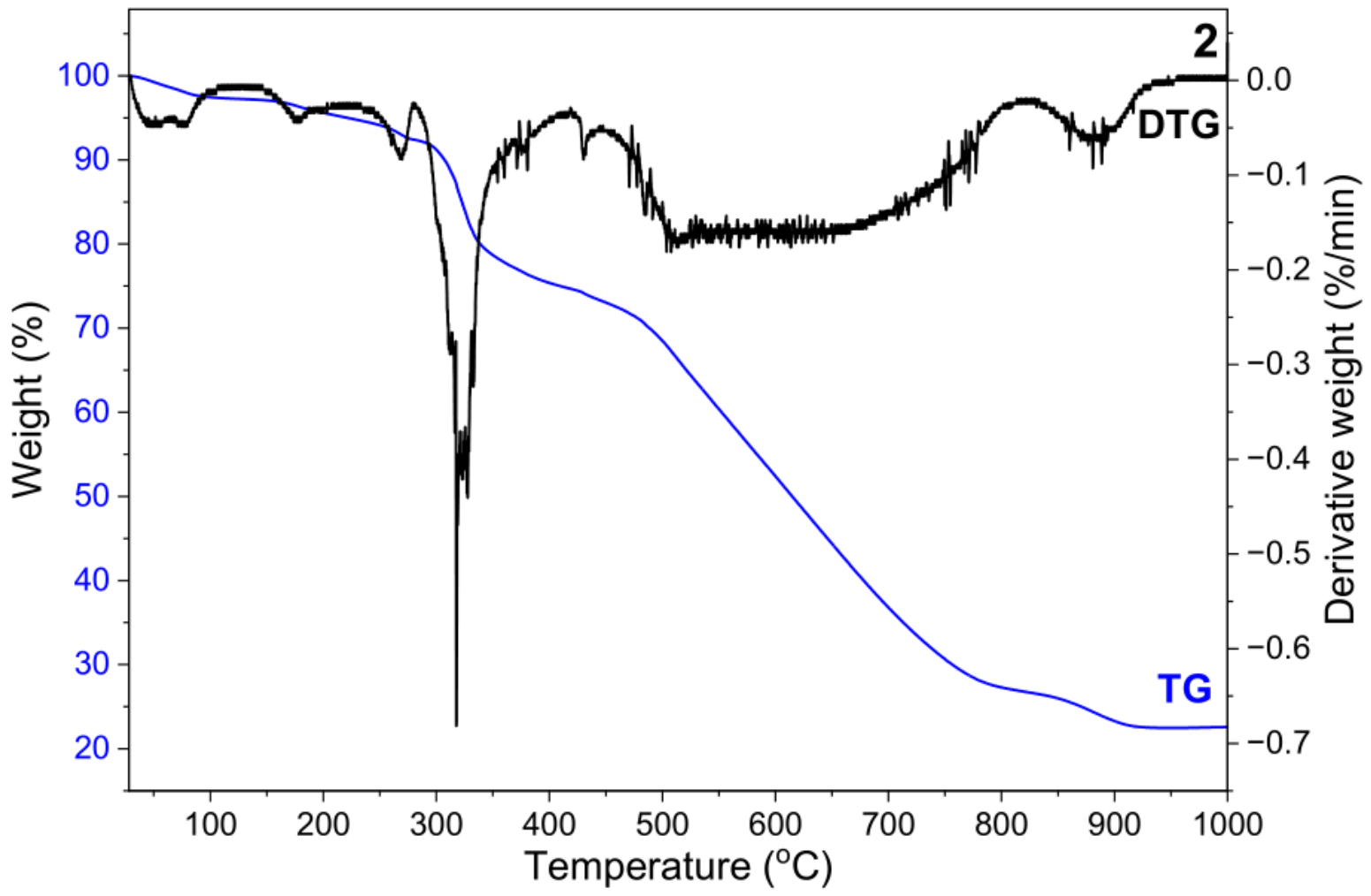

(c)

Figure S24. TG and DTG curves for **L** (**a**), complexes **1** (**b**) and **2** (**c**). The characteristic thermal phenomena and the corresponding stages of mass loss are discussed in the *RESULTS AND DISCUSSION* section, under the subsection titled *Thermogravimetry (TG)* (manuscript).

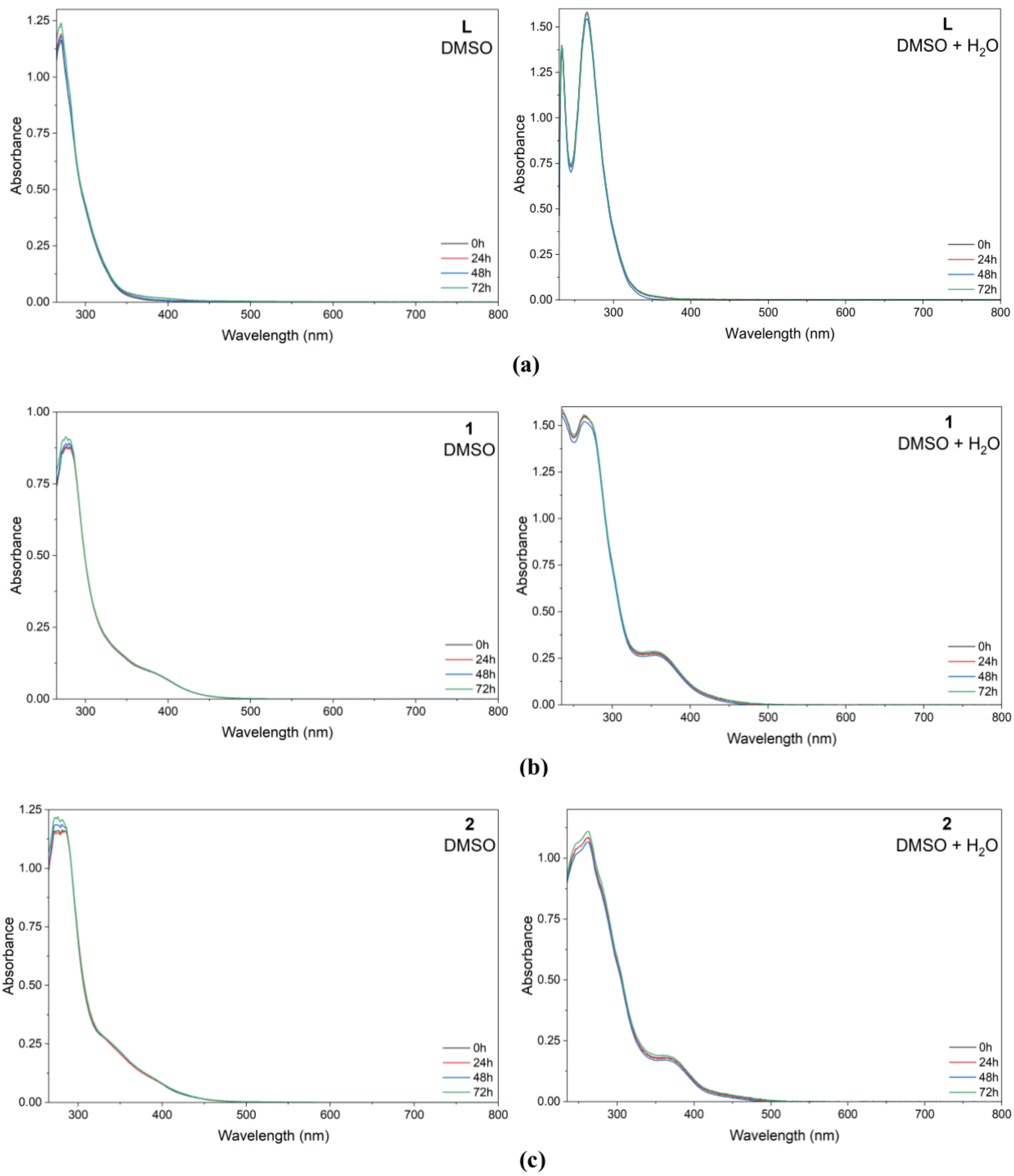

Figure S25. UV–Vis stability profiles of ligand **L** (**a**), complex **1** (**b**), and complex **2** (**c**) in DMSO (left) and DMSO/H_2O (right) monitored at selected time intervals (0 - 72 h).

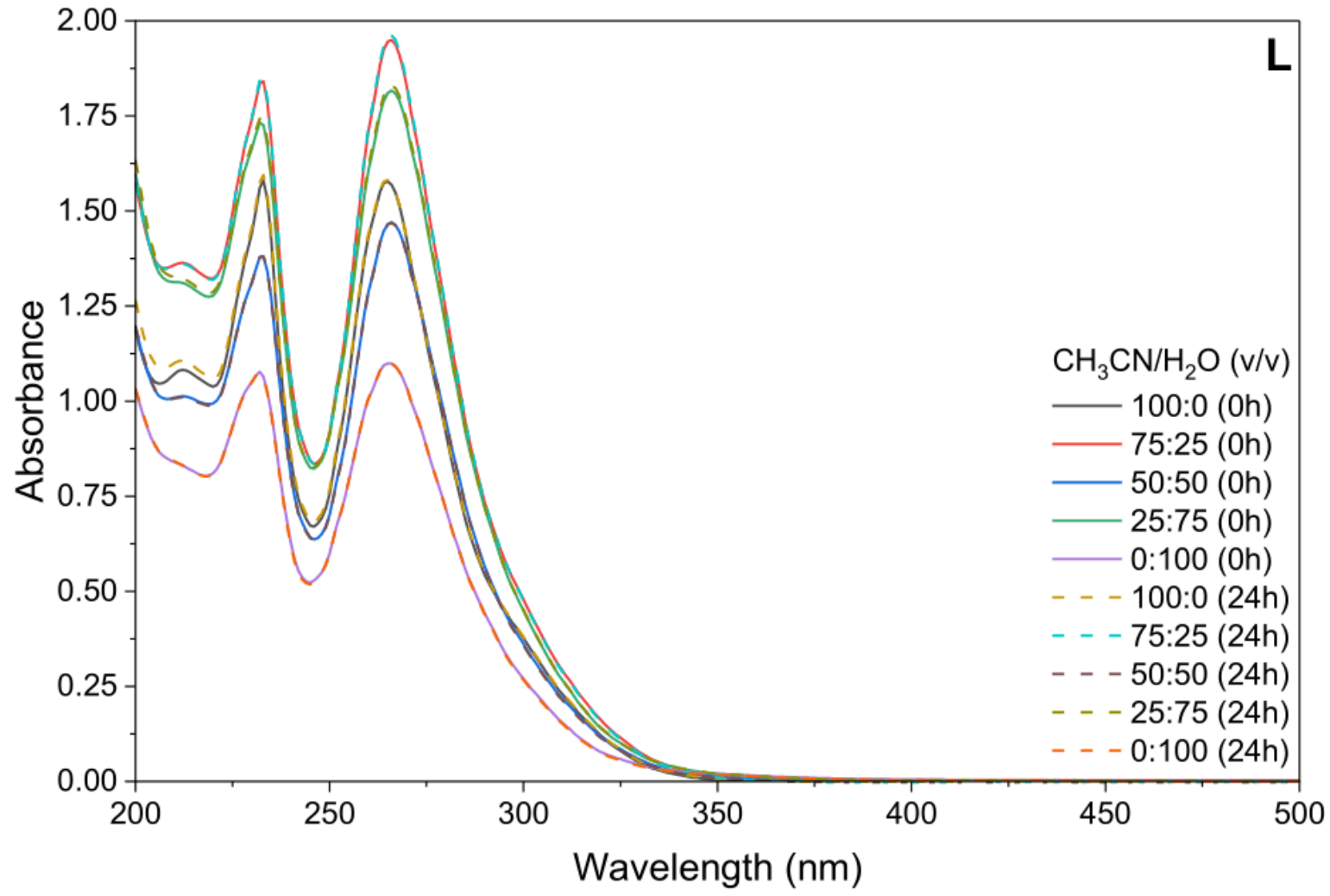

(a)

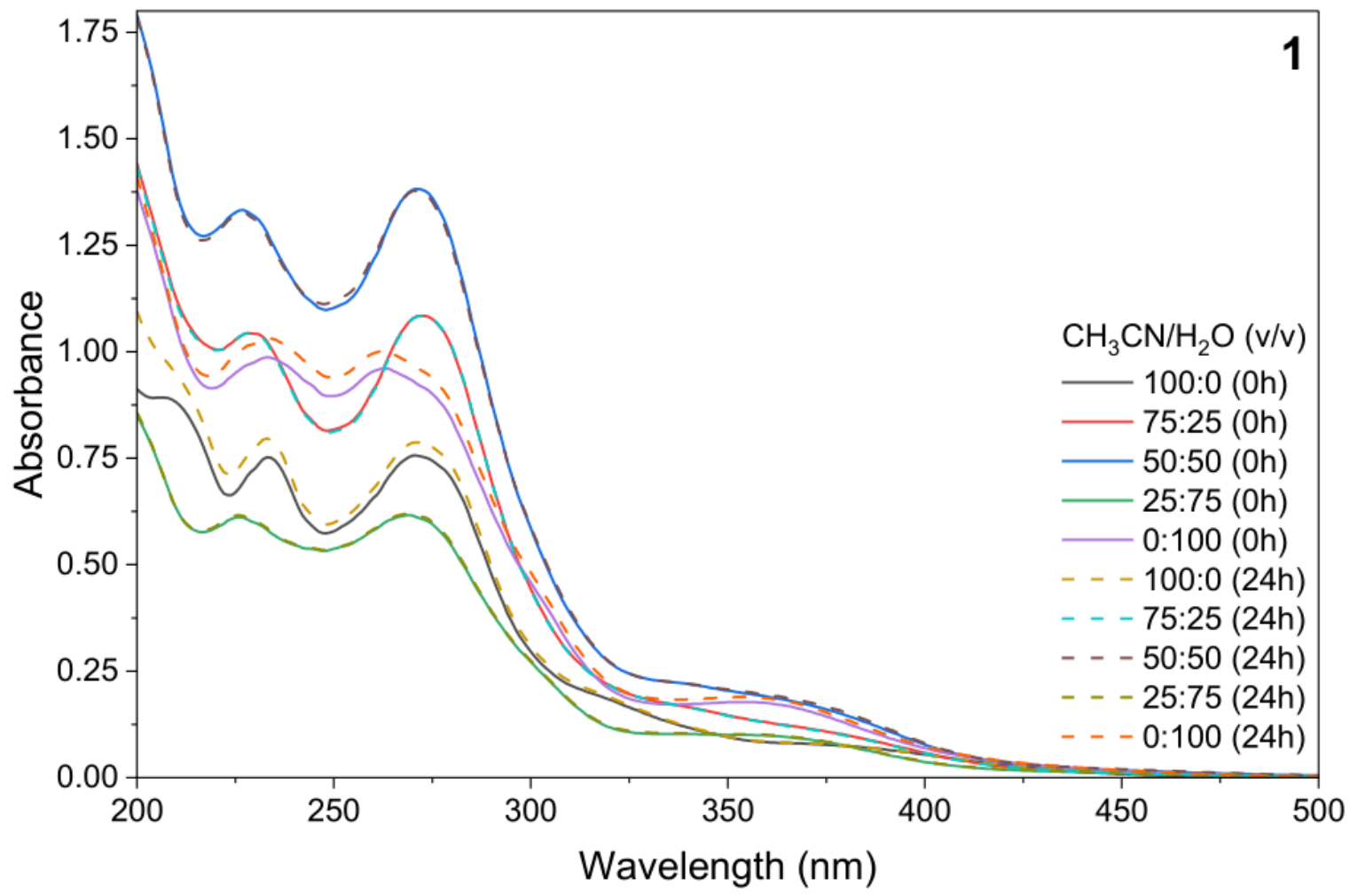

(b)

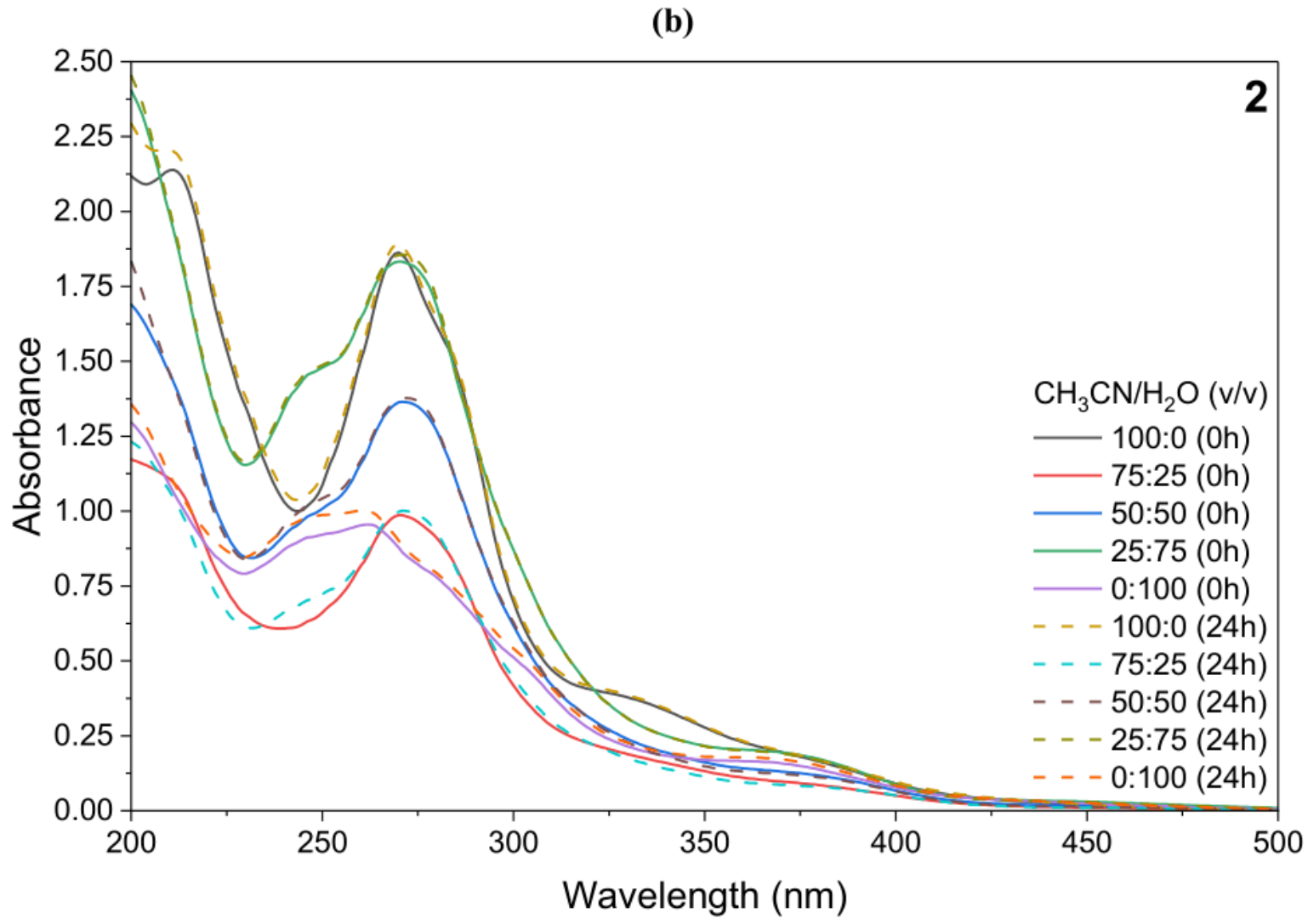

(c)

Figure S26. Evaluation of the stability of the tested compounds **L**/**1**/**2** in various ratios of acetonitrile and water based on UV-Vis spectra recorded immediately after the preparation of the solutions (0 h) and after 24 h.

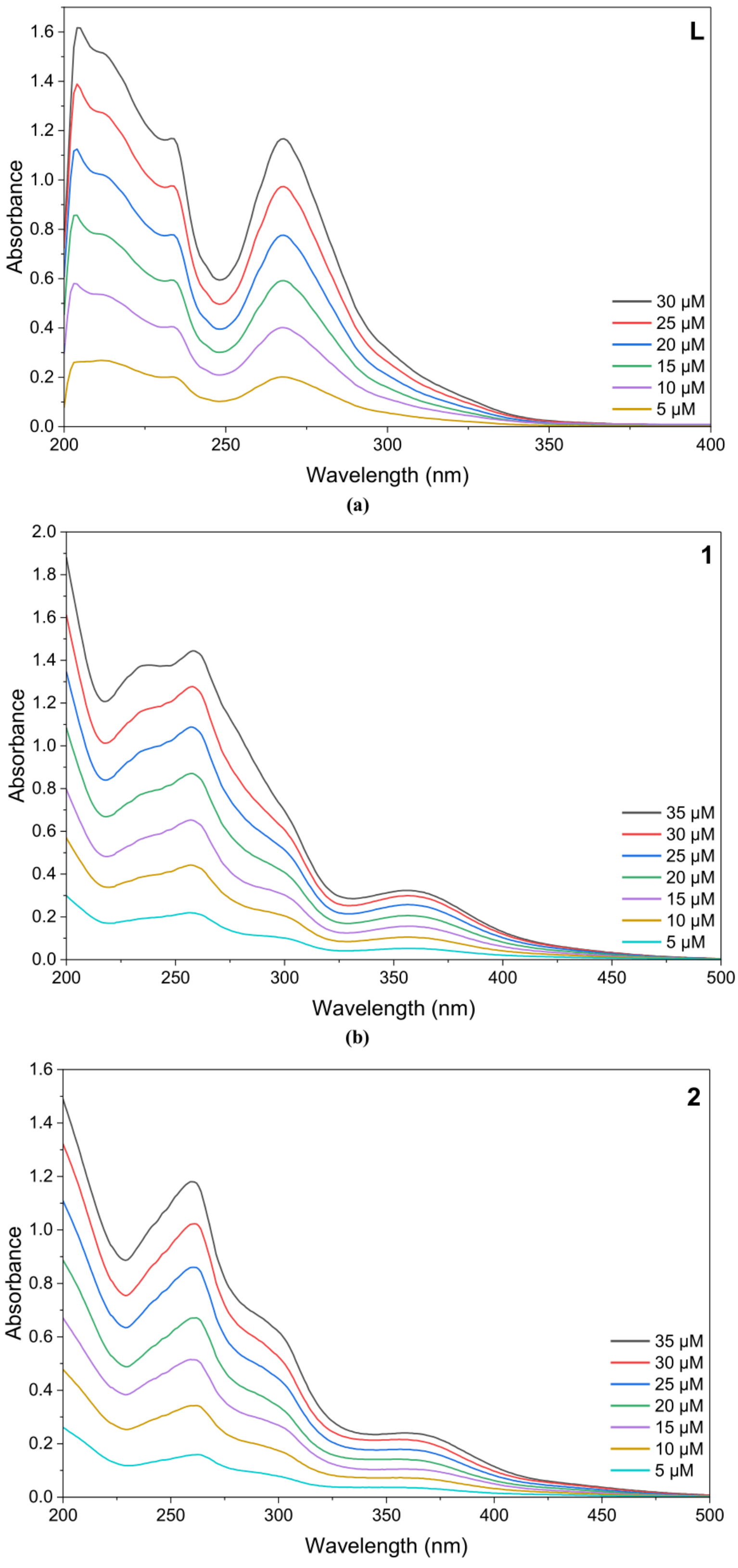

(a) (b) (c)

Figure S27. Calibration curve for **L** in octanol (**a**) for 6 concentration levels, complex **1** (**b**), and complex **2** (**c**) both in water for 7 concentration levels.

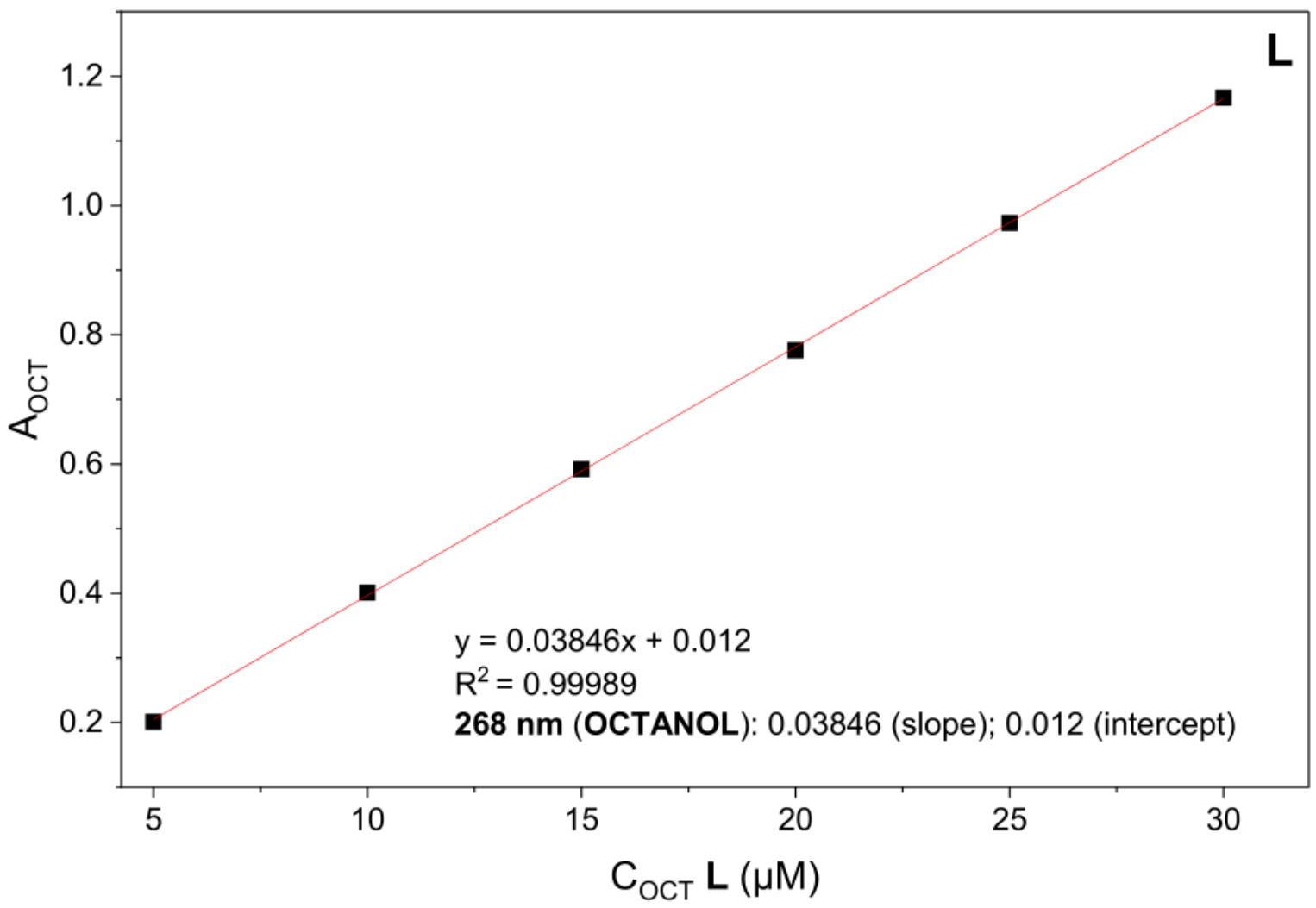

(a)

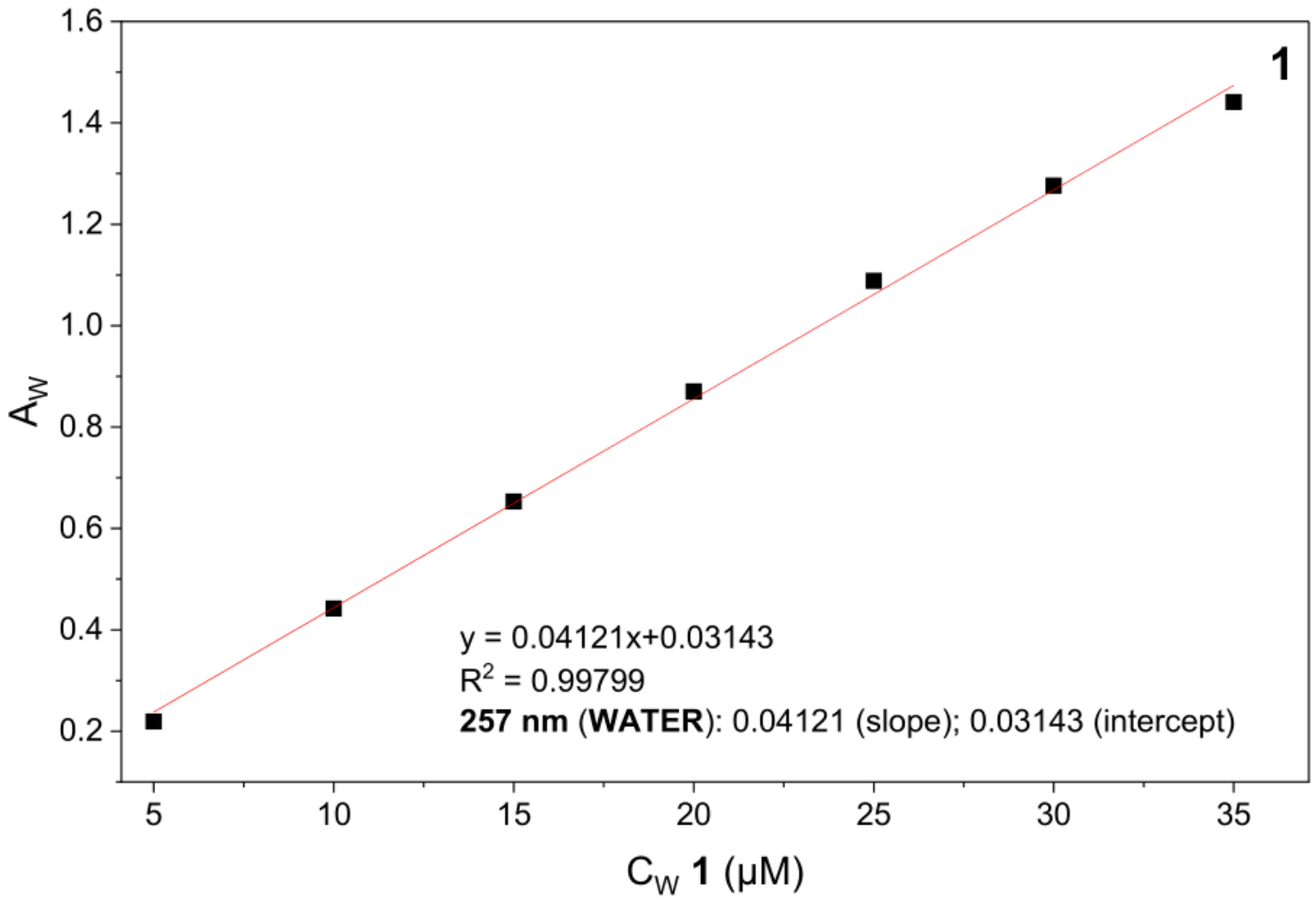

(b)

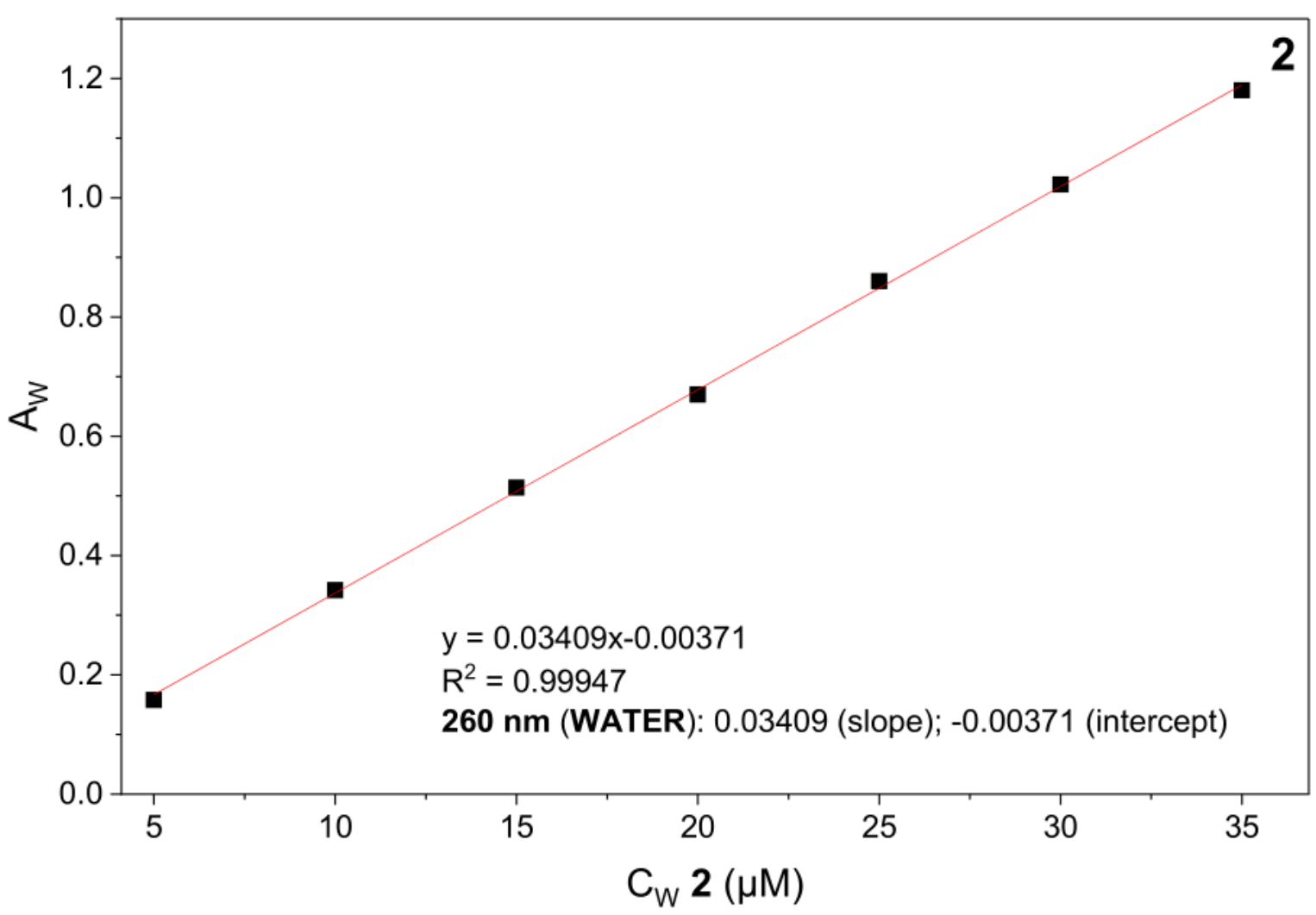

(c)

Figure S28. Linear relationships between absorbance and the concentration of (**a**) **L** in octanol at a wavelength of 268 nm, (**b**) **1** in water at a wavelength of 257 nm, and (**c**) **2** in water at a wavelength of 260 nm.

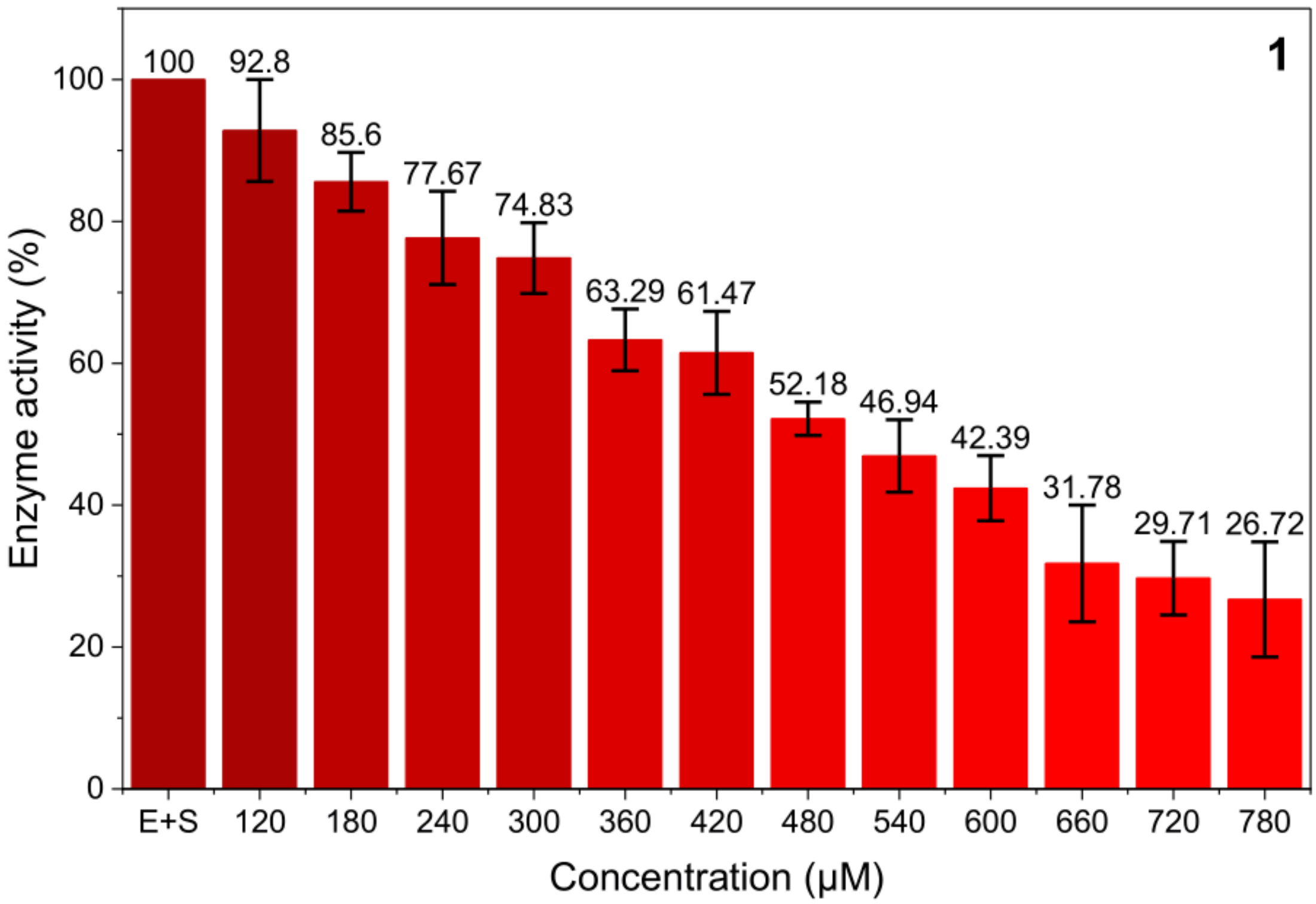

(a)

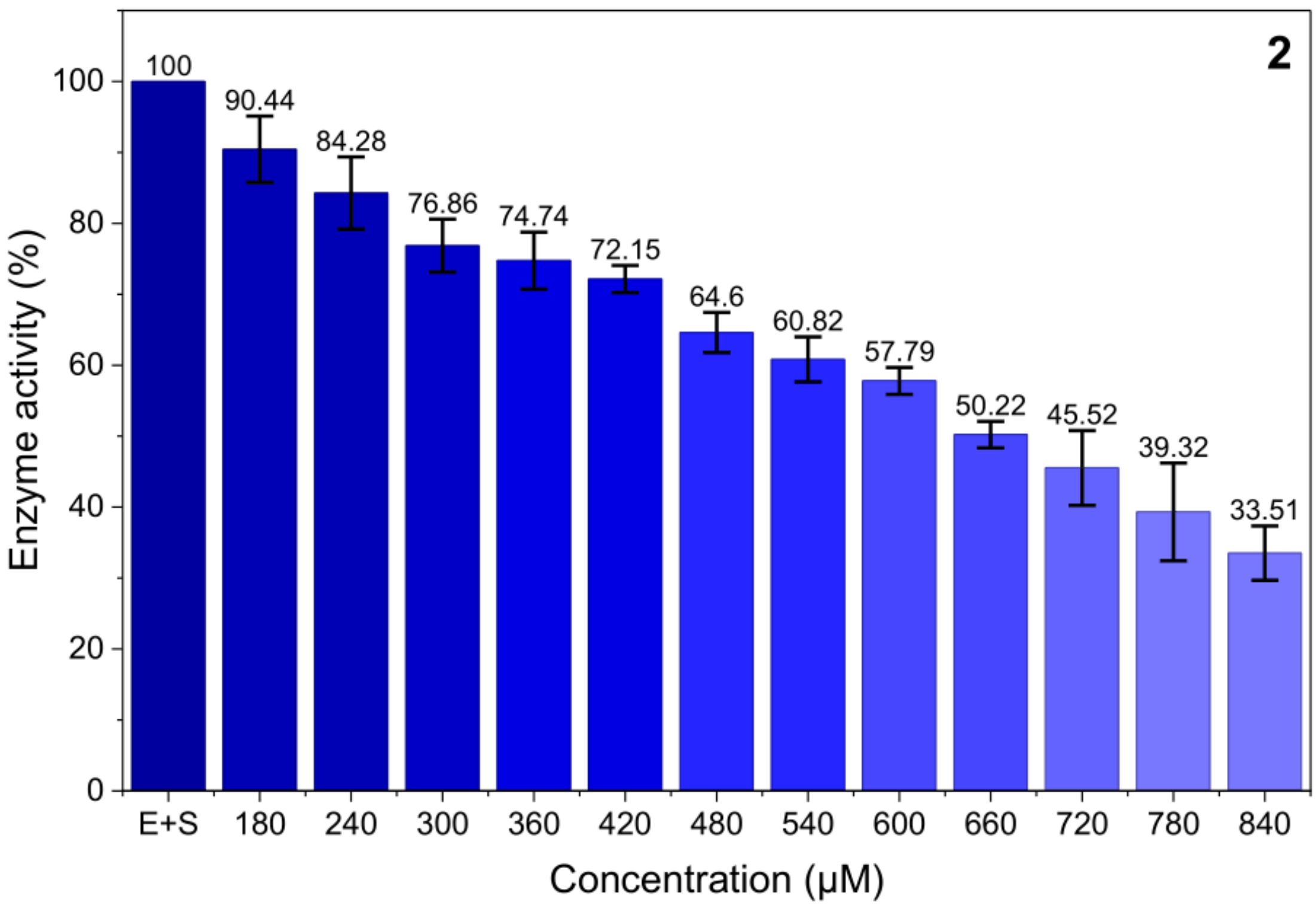

(b)

Figure S29. Effect of the concentration (μM) of test complexes **1** (**a**) and **2** (**b**) on enzyme activity (%) (CA). The first column (E+S) represents 100% enzyme activity, as no inhibitor was used there. Data are presented as mean ± SD (n = 6).

Table S4. The IC_{50} value, which indicates the concentration of a compound required to inhibit CA by 50%.

	L	1	2	Acetazolamide
**IC_{50} (μM)**	>1000	489.3<br>(469.5 to 510.1)*	649.7<br>(619.0 to 685.7)*	18.2[1]

*95% Confidence Intervals (CI)

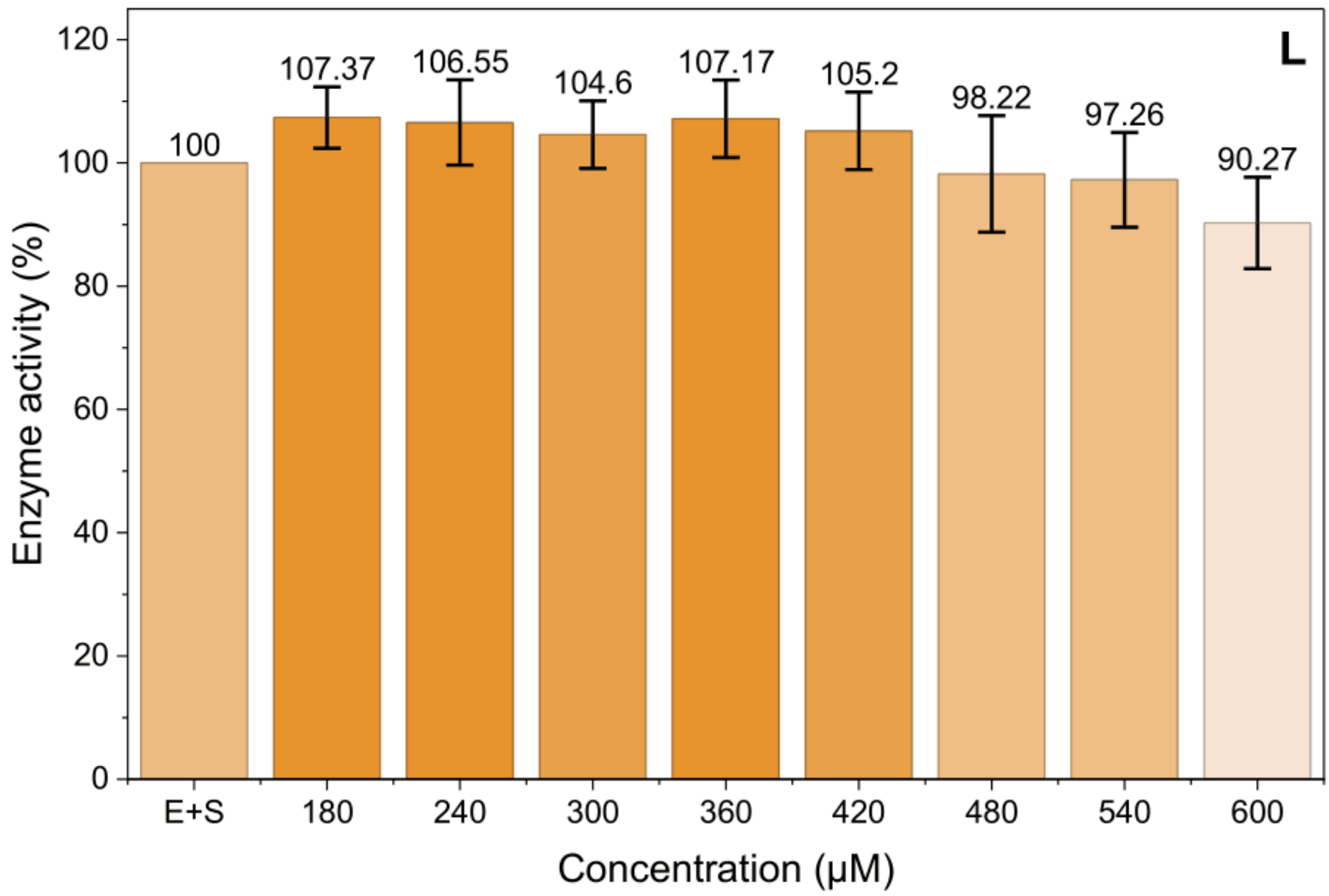

Figure S30. Preliminary results showing the effect of **L** concentration (μM) on enzyme activity (%) (CA). The first column (E+S) corresponds to 100% enzyme activity, as no inhibitor was used in this column. Data are presented as mean ± SD (n = 6).

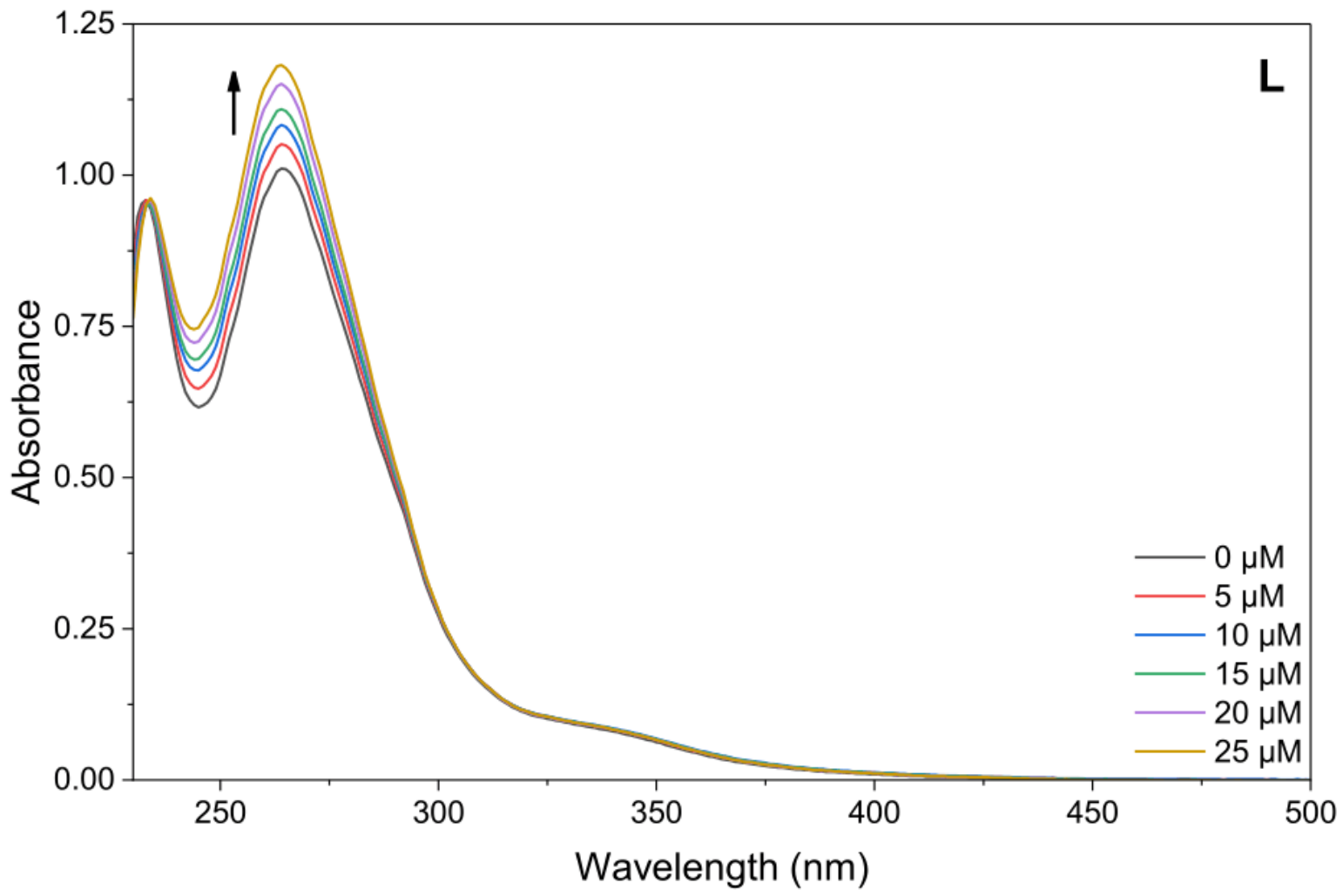

(a)

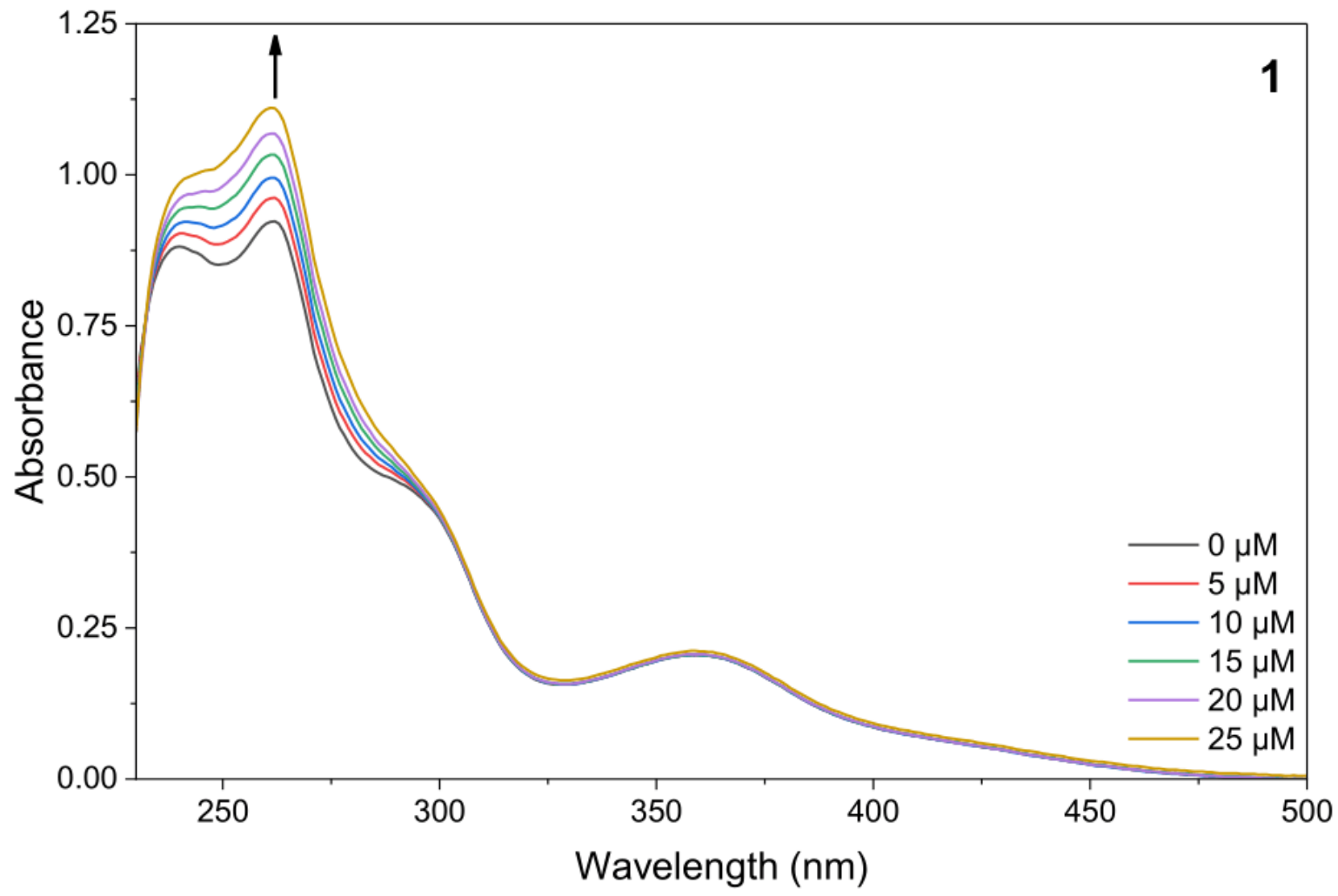

(b)

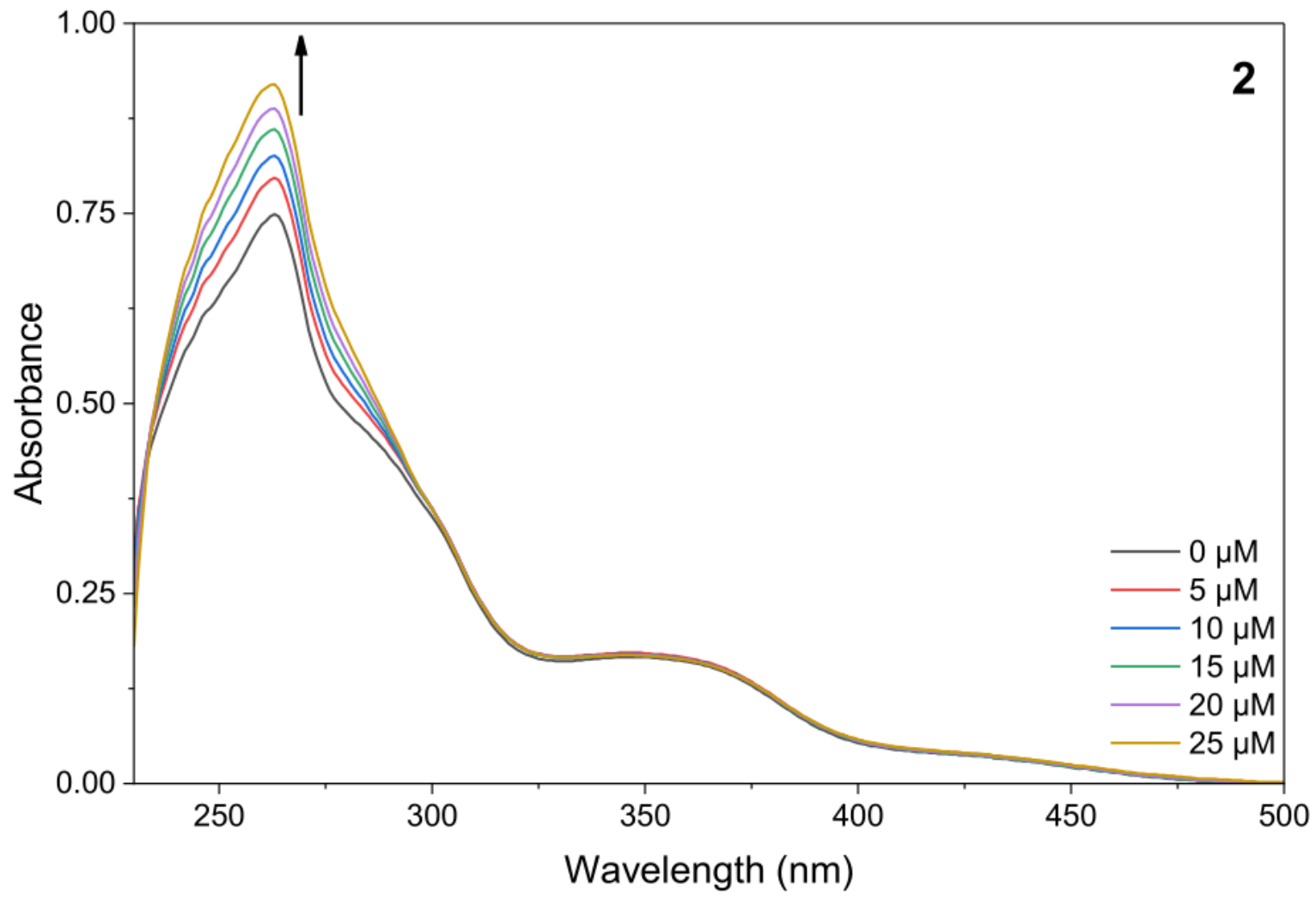

(c)

Figure S31. Changes in the UV–Vis spectra of CT-DNA induced by increasing concentrations of CT-DNA for **L (a)**, complex **1 (b)**, and complex **2 (c)**.

REFERENCES

(1) Rasool, A.; Batool, Z.; Khan, M.; Halim, S. A.; Shafiq, Z.; Temirak, A.; Salem, M. A.; Ali, T. E.; Khan, A.; Al-Harrasi, A. Bis-Pharmacophore of Cinnamaldehyde-Clubbed Thiosemicarbazones as Potent Carbonic Anhydrase-II Inhibitors. *Sci. Rep.* **2022**, *12* (1), 16095. https://doi.org/10.1038/s41598-022-19975-y.
